# Reward Pays the Cost of Noise Reduction in Motor and Cognitive Control

**DOI:** 10.1016/j.cub.2015.05.038

**Published:** 2015-06-29

**Authors:** Sanjay G. Manohar, Trevor T.-J. Chong, Matthew A.J. Apps, Amit Batla, Maria Stamelou, Paul R. Jarman, Kailash P. Bhatia, Masud Husain

**Affiliations:** 1 Nuffield Department of Clinical Neurosciences, John Radcliffe Hospital, Oxford OX3 9DU, UK; 2 Department of Experimental Psychology, University of Oxford, Oxford OX1 3UD, UK; 3 Institute of Neurology, University College London, London WC1N 3BG, UK; 4 Institute of Cognitive Neuroscience, University College London, London WC1N 3AR, UK; 5 National Hospital for Neurology and Neurosurgery, Queen Square, London WC1N 3BG, UK

**Keywords:** motivation, speed-accuracy trade-off, decision-making, dopamine, drift-diffusion model

## Abstract

Speed-accuracy trade-off is an intensively studied law governing almost all behavioral tasks across species. Here we show that motivation by reward breaks this law, by simultaneously invigorating movement and improving response precision. We devised a model to explain this paradoxical effect of reward by considering a new factor: the *cost of control*. Exerting control to improve response precision might itself come at a cost—a cost to attenuate a proportion of intrinsic neural noise. Applying a noise-reduction cost to optimal motor control predicted that reward can increase both velocity and accuracy. Similarly, application to decision-making predicted that reward reduces reaction times and errors in cognitive control. We used a novel saccadic distraction task to quantify the speed and accuracy of both movements and decisions under varying reward. Both faster speeds and smaller errors were observed with higher incentives, with the results best fitted by a model including a precision cost. Recent theories consider dopamine to be a key neuromodulator in mediating motivational effects of reward. We therefore examined how Parkinson’s disease (PD), a condition associated with dopamine depletion, alters the effects of reward. Individuals with PD showed reduced reward sensitivity in their speed and accuracy, consistent in our model with higher noise-control costs. Including a cost of control over noise explains how reward may allow apparent performance limits to be surpassed. On this view, the pattern of reduced reward sensitivity in PD patients can specifically be accounted for by a higher cost for controlling noise.

## Highlights

• The speed-accuracy trade-off in motor and cognitive control can be broken by reward
• Apparent limits of performance can be overcome by motivation
• A cost for reducing intrinsic neural noise quantitatively explains such improvements
• Reduced reward effects in Parkinson’s disease suggest an increased cost of control

## Graphical Abstract

**Figure:**
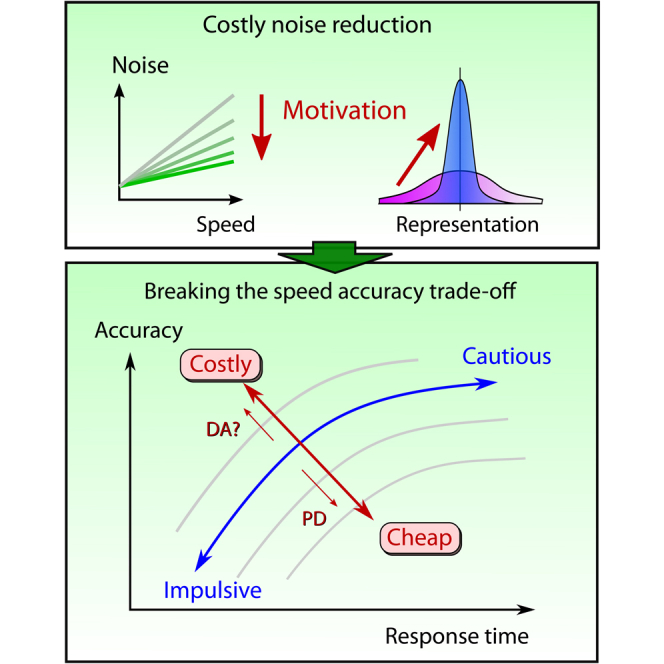

## Teaser

Manohar et al. investigate how motivation by reward can improve both speed and accuracy, apparently exceeding the limits of the speed-accuracy trade-off. They propose a cost for reducing intrinsic neural noise. Optimizing this cost predicts both motor and cognitive performance. The cost of control may be increased in Parkinson’s disease.

## Introduction

**Figure 1:**
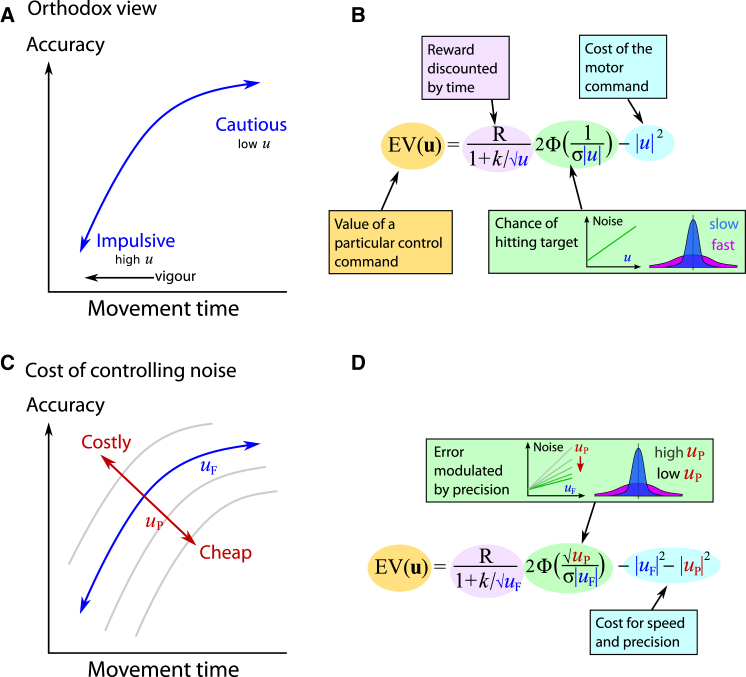
Breaking the Speed-Accuracy Trade-Off A conventional account of the speed-accuracy trade-off is shown on top (A and B). Lower panels illustrate the inclusion of a precision cost (C and D). (A) As movement time decreases (and speed increases), accuracy declines. This is because faster movements require larger forces, which are susceptible to proportionally higher noise. Because noise is taken to be rigidly proportional to motor command size, behavior is constrained such that accuracy depends on speed. This is captured by a speed-accuracy trade-off contour (illustrated in blue in the schematic). (B) Standard motor control models determine the optimal movement as the one that gives the highest average payoff. The expected value (EV) of a movement depends on the motor command **u**. EV can be expressed in terms of the reward, discounted by the movement time (pink). Larger motor commands give faster movements, leading to earlier reward, which is more valuable (here we use hyperbolic temporal discounting). The reward is further reduced by movement error, which reduces the probability of success (green). Under the orthodox view, this error is determined by noise proportional to the motor command. This means that faster movements will have less accurate endpoints and thus have a lower chance of winning a reward. The final term is the energetic cost of the motor command itself (blue). The balance between time and accuracy is governed only by the movement speed, which guarantees that speed and accuracy trade off with one another. (C) We suggest that reward has a motivating effect that permits both faster and more precise behavior. Incentivization by reward thus produces a change in a direction perpendicular to the blue line (red arrows), contrary to the speed-accuracy trade-off. By allowing both force and movement precision to be varied, our model allows two degrees of freedom over speed-accuracy space. (D) In order to explain violations of the speed-accuracy constraint, we introduced an additional precision command. The precision command reduces noise (*u*_P_, shown in red), complementing the usual force command (*u*_F_, blue). We propose that this command is itself costly, in the same way as the force command *u*_F_, leading to a cost term |*u*_P_|^2^ + |*u*_F_|^2^. Optimizing EV by selecting both the precision and force would allow accuracy to improve independently of speed, but constrained by this cost. Higher incentives allow a greater investment in precision, rather than a trade-off with speed, so genuine performance improvements are possible.

A fundamental and long-established finding in human and animal behavior is the phenomenon of speed-accuracy trade-off: when actions are performed faster, they are less accurate [1]. This principle applies widely across both motor and cognitive performance [2, 3]. Current theoretical approaches suggest that reward may increase the speed of actions, but at the cost of their accuracy. Recently however, some studies have reported that reward *simultaneously* increases both velocity and precision of motor control [4] and can reduce reaction times and error rates in decisions involving cognitive control [5, 6]. Here we provide a unified quantitative framework for how and why motivation by reward in fact contravenes the speed-accuracy trade-off, simultaneously improving both speed and accuracy in these diverse domains. According to our model, the speed-accuracy trade-off is not a hard barrier but rather a gray zone where the apparent limit of performance can be determined by reward (Figure 1). We apply the theory to both movements and decisions. We test our framework in healthy participants and also compare patients with Parkinson’s disease to a control group to demonstrate the role of reward and dopamine in accounting for the cost of control.

According to motor control theory, the speed-accuracy trade-off arises because larger or faster movements are subject to greater motor noise [7, 8]. Similarly, in the domain of cognitive control, models of decision-making predict a speed-accuracy trade-off, on the assumption that faster responding implies less time to weigh up evidence and thus more error-prone choices [9]. Since organisms prefer to obtain reward sooner [10, 11, 12, 13], high reward results in greater speed, or vigor, as measured by either movement time or reaction time [14, 15, 16]. Crucially, however, if noise ultimately limits performance, then the effect of reward on invigorating actions should lead to fast but inaccurate responding—in conflict with observed behavior [4, 5].

Current accounts, therefore, do not explain why we can perform well (i.e., be both fast and accurate) when motivated by reward but at other times are seemingly suboptimal [5, 17]. We propose a quantitative account of the effects of reward in terms of paying the *cost of control.* Such a factor has been invoked recently to explain qualitatively how incentive might increase “cognitive control” by overcoming a cost [18, 19, 20]. Here, we consider a hidden *precision cost,* analogous to the cost of motor commands in optimal control theory.

Numerically, we propose that the brain might put a fixed price on attenuating noise by a certain proportion. Noise-reduction mechanisms might include corrective feedback signals [21], allocating more resources to representing that signal [22], or attenuating currently irrelevant information [23]. Each of these mechanisms may incur a cost to the organism in terms of opportunity cost, neuronal resources, and/or energetic cost. However, regardless of the ultimate nature of the cost, optimizing the level of precision provides a unified mathematical way of describing the deployment of resources such as effort, attention, and executive control [24].

Our framework makes several key predictions. First, when applied to optimal motor control, the precision cost leads to the prediction that when incentives are high, movements can become both fast *and* precise. Second, when applied to decision processes, in the form of a rise-to-threshold model of reaction time (RT), a noise-reduction cost can also quantitatively explain motivational effects on RTs and error rates. From a cognitive neuroscience perspective, this as approach allows quantification of “effort costs” of deploying increased attention—effectively amplifying relevant sensory signals or suppressing irrelevant ones [25]—when the reward are high.

To test our theory and quantify how reward can make us apparently “more optimal,” we devised a novel saccadic task in which participants have to look toward a target quickly, while avoiding a salient early-onset distractor (Figure 5). The velocity of a saccade has often been regarded as rigidly determined by its amplitude [26, 27, 28], until recent studies demonstrated modulation by reward [4, 14, 16]. In our experiment, by manipulating incentives on each trial, we measured exactly how reward increases saccade velocity (speed) and endpoint accuracy. Furthermore, we were able to separately index cognitive control by measuring errors to the distractor and their relation to RT. Our model accounted well for the observed behavior.

Next, we investigated whether patients with Parkinson’s disease (PD) show altered motivational effects of reward. In both animals and humans, dopaminergic stimulation increases willingness to exert an effortful force for reward [29, 30] without trading speed for accuracy [31, 32]. In PD, dopamine depletion leads to slow, small movements. An attractive explanation for this is that PD patients experience greater costs for their movements [33, 34]. Indeed, it has been proposed that reward might potentially exert its effects on vigor of response via dopamine [11, 32]. Applying our cost-of-control framework provides a parsimonious explanation for how this might arise. Patients with PD might be impaired in reducing internal noise in response to reward, manifested as a reduced ability to increase movement speed in response to incentive. This would explain why PD patients are sometimes less precise in motor tasks [15] yet can still generate a range of movement speeds [15]. To examine the proposition that dopamine depletion might increase the cost of attenuating noise, we tested patients with PD and fitted the model to healthy participants’ and patients’ data.

## Results

### Modeling Reward Incentives in an Optimal Control Framework

#### Current Conceptual Frameworks Cannot Explain Behavior

When reward is available, we react faster [13, 32]. To explain how reward induces urgency, or time pressure in responding, it has been suggested that a high ongoing rate of reward may encourage fast frequent responding, minimizing opportunity cost [11]. Time pressure can be expressed in terms of temporal discounting [10, 12], in which a delayed reward is worth less. A commonly observed pattern of devaluation over time is “hyperbolic discounting” [10, 13], in which rewards delayed by time *T* are worth less by a factor of 1 + *kT*, where *k* is known as the discount rate [35].

Conversely, at least two factors favor *slower* movements. First, fast movements require more energy. Within the framework of motor control, a fast movement results from a larger “control command”—e.g., the firing rate of a motor neuron. The energy expenditure corresponds to a cost, which is presumed to be related to size of the control command [36, 37]. Second, motor noise has been assumed to increase proportionally to the size of the control command [26], such that faster actions are less likely to be successful (e.g., arrive on a target).

These constraints lead to an optimum speed, since faster movements have higher energetic costs and error rates, which must be “paid” by gaining more reward sooner. To quantify this, we consider a motor command **u**(*t*), representing a set of instructions varying over time, for example, the neural output to muscles. We may then weigh up the expected value (EV) of an action, which depends on **u** [36] (Figure 1B). The optimal movement speed can be determined by finding **u** that maximizes EV. The utility of the reward *R* is scaled by the probability of the movement arriving on-target *P*_*win*_ and must balance the energetic cost |**u**|^2^:

Equation (1) $$\begin{array}{c} EV\left(u\right)=R\times D\left(u\right)\times P_{win}\left(u\right)-{\left|u\right|}^{2}\\ \text{Expected}\;\text{value}\;\text{of}\;\text{action}=\text{Reward}\times \text{temporal}\;\text{discount}\times \text{probability}\;\text{of}\;\text{reward}\;\text{given}\;\text{a}\;\text{motor}\;\text{command}-{\left(\text{size}\;\text{of}\;\text{motor}\;\text{command}\right)}^{2} \end{array}$$

In the orthodox model, higher reward increases the relative importance of time costs *D*, relative to energetic costs |**u**|^2^. High reward thus favors fast movements (larger **u**) [12, 13], which are, however, subject to greater neural noise [7, 23]. Thus, according to previous accounts, increasing the reward shifts behavior from cautious, accurate responding to impulsive, inaccurate responding [37] (Figure 1A, blue line). Crucially, this form of cost function does not permit both accuracy *and* movement speed to increase simultaneously without compromising movement amplitude. To account for such effects, it is necessary to invoke a second dimension of control: a cost for attenuating motor noise, or equivalently, increasing signal-to-noise ratios. Put simply, we might choose to invest in noise reduction, if it were advantageous (Figure 1C). To make the cost explicit, we can include in **u** an additional control signal that reduces noise.

#### Applying a Noise-Reduction Cost to Motor Execution

We now consider a simple one-dimensional movement and split the command **u** into two components: a standard motor command *u*_F_ signaling force, and also a novel precision control signal *u*_P_. For the optimal movement, a force/precision pair **u** = [*u*_F_, *u*_P_] must be chosen that maximizes value (Figure 1D). The larger the precision command *u*_P_, the lower the resultant noise in the force generated by *u*_F_. The actual mechanism that cancels noise might be complex, e.g., involving numerous internal signals, but our notion of a precision signal abstracts away the actual signals that correct for noise and retains only their cost and efficacy. (See Supplemental Experimental Procedures for a general form and discussion on how noise might be attenuated in the brain.) The probability of obtaining the reward *P*_*win*_(**u**) will increase with precision *u*_P_ and decrease with force *u*_F_. But because we treat *u*_P_ in the same way as a control signal, it incurs a cost |*u*_P_|^2^.

**Figure 2:**
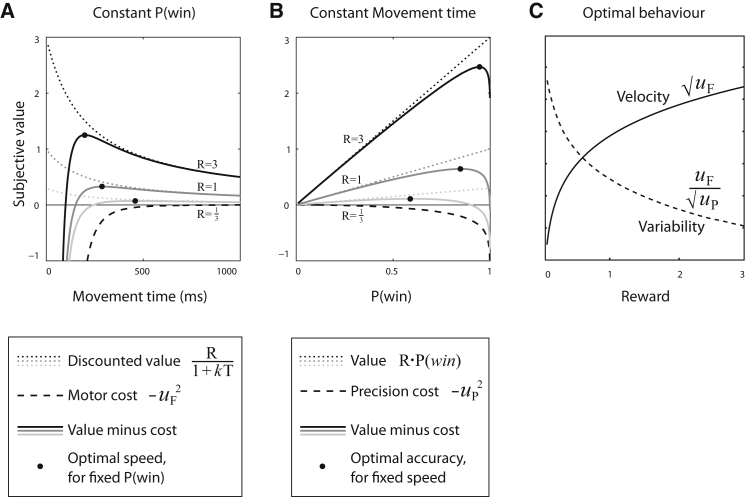
The Costs of Inaccuracy, Slowness, and Control Our model incorporates three costs: inaccuracy is expensive because errors are not rewarded, slowness is expensive because a reward is less valuable when delayed (temporal discounting), and we further suggest that control over errors is itself expensive. Therefore, a three-way balance obtains. (A) The vertical axis represents the subjective value of a given movement. For a given reward *R*, temporal discounting causes the reward’s subjective value to fall as movement times get longer (dotted lines). However, moving faster entails greater energy expenditure (dashed line; negative value implies a cost). The net value (solid lines) is the sum of these two components, showing that the optimal movement is faster with higher reward [36]. (B) The probability of winning a reward, *P*_*win*_, could depend on the endpoint of the movement being accurate. The cost of precision allows the endpoint variability to be reduced at a cost. The probability of landing on a fixed-size target can be increased if a “precision cost” is paid (dashed line). Precision increases the average gain from winning (dotted line), as shown for three different reward levels. The net value (solid lines) illustrates that the optimal movement is more precise with increasing reward. (C) If both speed and accuracy are both free to vary, the optimum pair can be determined as a function of reward. Reward increases the optimal movement speed and, when temporal discounting is not too large, reduces the optimal endpoint variability.

**Figure 3:**
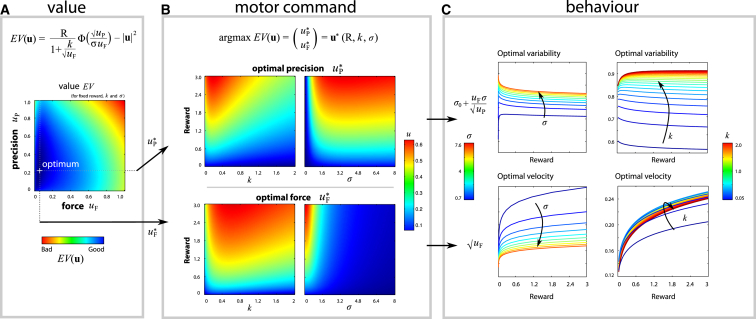
Optimal Control Model to Explain the Effect of Reward Incentives In order to account for the ability of reward to improve both speed and accuracy, we hypothesized that in addition to a “vigor” or force signal (*u*_F_) that determines a movement’s speed, individuals are also able to select a “precision” signal (*u*_P_) that determines the amount of variability in a movement. Crucially, this precision signal is also costly. (A) Each given motor command, i.e., a pair of force and precision **u** = (*u*_F_, *u*_P_), has an EV. The image shows EV as a function of **u**, with the best combination as blue and worst as red. The value depends on three effects. First, the reward available is temporally discounted by the time taken by the movement, e.g., by hyperbolic discounting $1/\left(1+k/\sqrt{u_{F}}\right)$. Second, this reward is only obtained if the movement is on target. We assume a Gaussian variation Φ of the endpoint proportional to the size of the motor command. Third, although we can go faster to reduce temporal discounting (increasing *u*_F_) and be more precise to reduce error (increasing *u*_P_), both of these incur a cost proportional to the squared control signal, u^2^. This leads to an optimal combination of force and precision for each movement, **u**^∗^. (B) The optimal motor command for a situation depends on the reward level *R* and on two subject-specific parameters: the discount rate *k* and the noise-control cost σ. The optimal precision (upper panels) and force (lower panels) both increase with increasing reward (y axis), indicating that reward induces greater “spending” on both speed and accuracy. However, precision and force are differentially influenced by reward, and the balance depends on the urgency (temporal discount, *k*, left panels) and error constraints (encapsulated by σ, right panels). (C) The optimal commands determine the velocity and duration of each movement and the amount of variability for a desired movement amplitude. Reward always increases velocity (lower panels). However, variability may increase or decrease with reward (upper panels), depending on σ and *k*. A subject with minimal discounting (e.g., *k* < 0.5) becomes less variable with higher reward, whereas a subject with high discount rates (e.g., *k* > 1) in fact tends to become *more* variable with higher reward (upper panels) as they are under greater time pressure, i.e., trading speed for accuracy. These effects are re-plotted on different axes in Figure S5.

When motor noise and accuracy are made irrelevant (e.g., for very large targets), then increasing reward simply increases the cost of time relative to energetic costs. Subjects are consequently more willing to exert more effort to move faster, so higher reward increases optimal speed (Figure 2A), as in the orthodox view [16, 36]. Conversely, if speed is ignored and only accuracy and precision are considered, then a new trade-off occurs between the cost of precision and the cost of errors. Since precision improves the probability of success but is expensive, there is an optimal level of accuracy which increases when more reward is on offer (Figure 2B). Crucially, when both precision and force are allowed to vary simultaneously, reward has the effect of increasing the optimal velocity while also reducing motor variability (Figure 2C). For each reward level *R*, there is a particular combination of force *u*_F_ and precision *u*_P_ that maximizes EV (Figures 3A and 3B), corresponding to an optimal saccade velocity and endpoint variability (Figures 3C and S5). The optimum will depend on an individual’s temporal discount rate *k* and the noise parameter σ. To account for the possibility that not all noise may be controllable by a system (e.g., noise in the effector itself), an additive baseline noise term σ_0_ can be included. In this case, σ_0_ represents a participant’s fixed motor noise, whereas σ represents the relative cost of precision, compared to energetic (force) cost.

#### Application of Precision Control Costs to Rise-to-Threshold Models

**Figure 4:**
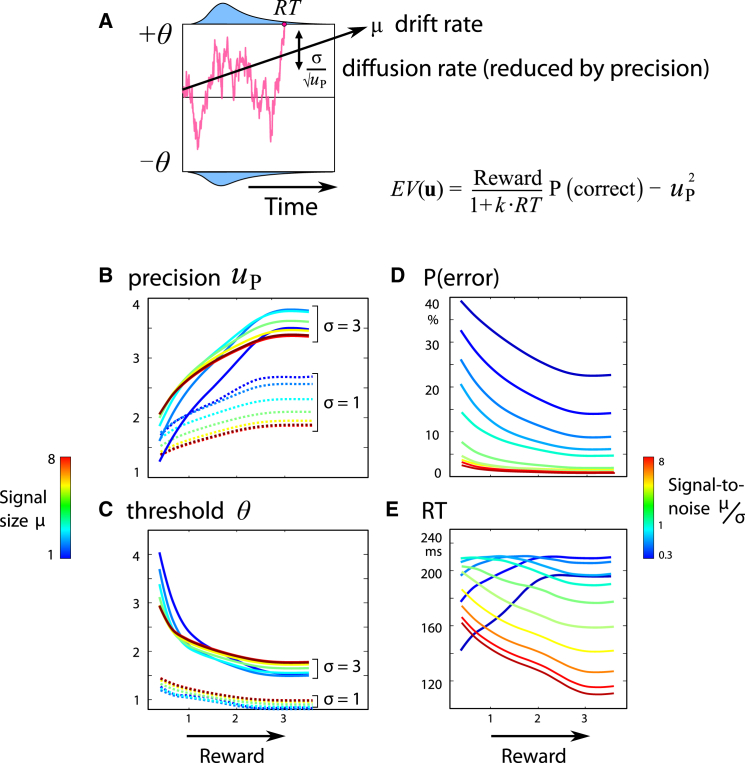
Cost of Control Applied to Drift Diffusion (A) The drift-diffusion model assumes an accumulator integrating incoming information at a fixed drift rate (μ), subject to noise (σ), until it reaches a threshold (θ). The red line illustrates the trajectory in an example trial. Blue histograms indicate the distribution of response times for correct and incorrect responses. Increasing the threshold leads to more accurate decisions, at the cost of slower responses. In order to account for violations of the speed-accuracy trade-off, we introduced a costly noise-reduction parameter (*u*_P_), similar to our extended motor control model. This permits the optimal combination of threshold and precision to be chosen. (B–E) Simulations provide reaction times and accuracy (i.e., when the decision terminates, and whether it is at the positive or negative boundary) for a variety of signal sizes (μ), noise (σ), and reward levels (*R*). For each condition, the optimal pairing of threshold (θ) and precision (*u*_P_) is selected to maximize value (EV). The value of a pair was calculated as accuracy multiplied by reward, temporally discounted by the reaction time. (B and C) As reward increases, it is optimal to increase the precision and lower the decision threshold. (D) This leads to improved accuracy with reward. (E) When the signal-to-noise ratio is high, reward encourages faster responding; however, when the decision is noisy, reaction times actually increase with reward, despite falling thresholds—producing a speed-accuracy trade-off.

Controlling noise might be relevant not only for online motor control but also for deciding which action to take, and when. For decisions, standard speed-accuracy trade-offs are accurately predicted by rise-to-threshold models such as the drift-diffusion model (Figure 4A). In this model, a decision variable accumulates information over time about which action to select. When the evidence reaches a threshold, an action is triggered. Lowering or raising the decision threshold θ gives rise to fast, error-prone choices or slow, accurate responses, respectively [9, 38]—trading speed for accuracy. By default, the signal-to-noise ratio μ/σ is assumed to remain constant. Attention or alertness might augment the gain of signal over noise, but this is often postulated to be “effortful,” currently without a quantitative prediction [25]. We suggest that these factors might be described in terms of a top-down control signal *u*_P_ that improves the signal-to-noise ratio in the accumulator [39]. Crucially, this noise-reduction signal may carry costs, which increase with *u*_P_. The threshold/precision pair **u** = [θ, *u*_P_] may then be optimized to maximize value (see Supplemental Experimental Procedures). The model predicts that reward could improve the signal-to-noise ratio of decisions when it is economically feasible. The control cost determines, for the first time quantitatively, how motivation leads to fast, accurate responses—i.e., “true improvement” in performance. Simulations of drift diffusion were run to obtain the optimum threshold and precision for various reward levels and signal-to-noise ratios. These simulations showed that reward increased accuracy (Figures 4B and 4D), but also shortened RTs, under conditions when signal-to-noise μ/σ was high (Figure 4E, red lines).

### Testing the Effects of Reward Using Saccades

**Figure 5:**
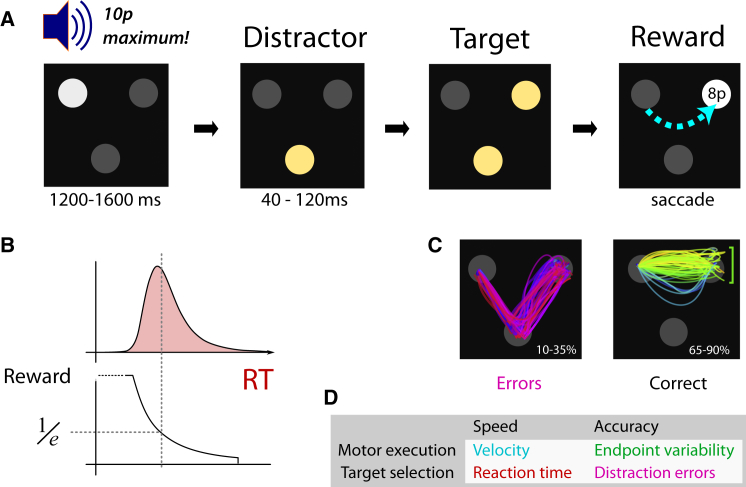
Oculomotor Capture Task with Trial-wise Incentives (A) Three equidistant discs were dimly illuminated. At the start of each trial, participants had to fixate one disc, which was brightened. A recorded voice gave an auditory reward cue of “0p maximum,” “10p maximum,” or “50p maximum,” which indicated the maximum amount of money that could be won if participants were fast to look at the target on that trial. After a variable foreperiod, the other two discs were illuminated asynchronously, with a delay of 40 to 120 ms. Participants were instructed to look as fast as possible to the second disc. Thus, the first onset acted as an early onset distractor, and the second disc indicated the target. (B) After gaze arrived at the target, participants were rewarded according to reaction time. Reward was calculated as a fraction of the maximum available, using an exponential falloff. The falloff was determined adaptively using quantiles of the last 20 trials, in order to maintain the difficulty level over the course of the experiment. (C) On approximately 30% of trials, gaze was “captured” by the distractor (errors), resulting in a brief saccade to the first disc, followed by a corrective saccade to the target. The trajectory of gaze was classified according to whether the first saccade terminated on the target or on the distractor. Correct trials exhibited a variety of curvatures; each trial is colored according to the initial direction of the eye velocity. (D) The task provided four measures of performance. Both speed and accuracy could be examined for motor execution of the saccade and for selection of the correct target.

We devised a novel saccadic task to measure how reward impacts upon both speed and error in movements and decisions. Trials started with participants fixating one of three gray discs arranged in a triangle (Figure 5A). They were instructed to move their eyes as fast as possible to the disc that lit up second. Participants were told that the first disc that was illuminated would be a distractor and the second would be the target. The faster they arrived at the target, the more money they won. Critically, during the 1.2 s foreperiod, a recorded voice was played back, speaking the maximum reward available on this trial. Three reward levels were used: 0 pence (p), 10p, or 50p (1p ≈ 1.5 US cents). This indicated the amount that could be won if a saccade was made rapidly to the target. Next, the fixation disc was dimmed while one of the other discs was brightened (the distractor). After 80 ms, the remaining disc (the target) brightened also. The display remained until gaze arrived at the target. The task is a variant of the double-step paradigm [40] and aimed to maximize oculomotor capture by the salient distractor [41].

Reward was calculated adaptively on each trial dependent on when gaze arrived at the target and was displayed numerically (Figure 5B). The target location was then used as the starting point for the next trial. Participants performed 72 trials of each of the three reward levels, intermixed. The task yielded four performance measures: oculomotor capture errors (classified offline according to whether the first saccade endpoint was closer to the distractor than the target; Figure 5C), RT measured as time from distractor onset until initiation of saccade, peak velocity of correct saccades, and the variability in amplitudes of this first saccade. This gave two measures of speed, and two measures of accuracy, for the motoric and target-selection aspects of the task (Figure 5D).

#### Reward Breaks through the Speed-Accuracy Trade-Off in Healthy People

**Figure 6:**
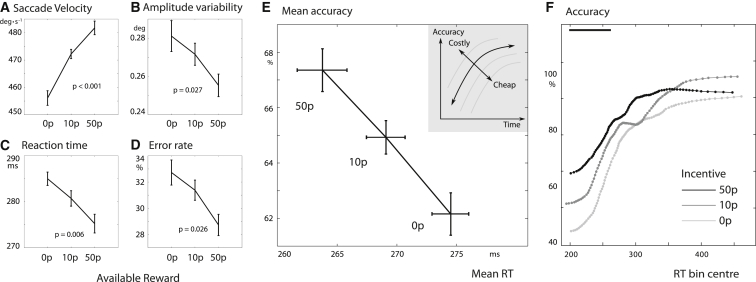
Effects of Reward on Saccades in Healthy Participants (A) For correct trials, the mean peak velocity of saccades increased with higher incentives, demonstrating invigoration by reward. Error bars indicate within-subject standard error. (B) Endpoint variability (standard deviation of the saccade amplitudes) in each condition became less variable with increasing incentives, indicating that reward can improve motor precision. (C) For correct trials, RTs were faster for higher incentives. (D) The rate of oculomotor capture (proportion of trials on which the first saccade after the onset was directed to the distractor, i.e., error trials) was reduced with increased incentives, indexing improved accuracy. Error timings are shown in Figure S2 and times to correct errors in Figure S3. (E) Plotting the data from Figure 5 as accuracy versus RT (where accuracy is defined as percentage of responses that were directed to the target and not to the distractor) demonstrates how, with increasing incentives, reaction time decreased and accuracy simultaneously improved. The inset shows how this relates to Figure 1C: the speed-accuracy trade-off is broken. (F) Conditional accuracy plot shows how, for a fixed reward level, accuracy improved with increasing RT, but this relationship was shifted by incentives, with the greatest differences evident at short RTs. The gradient of each curve is always positive, indicating that for a trials within a single reward level (i.e., constant incentive), the speed-accuracy trade-off held. The plot shows the proportion of saccades that went to the target, in a sliding window along the RT distribution, width 20% quantiles. Patient data and model are shown in Figure S1.

In the first experiment, we studied the effects of reward in 39 healthy participants. Reward significantly increased speed, in terms of both faster saccade velocities and shorter RTs. In addition, it also improved accuracy, with reduced oculomotor capture rates and lower endpoint variability. With high incentives (50p), the average peak saccade velocity was of 474°s^−1^ ± 13°s^−1^ (SEM) compared to 452°s^−1^ ± 11°s^−1^ with no incentive (repeated-measures ANOVA, main effect of reward F(2,76) = 20.8, p < 0.001; Figure 6A). RTs were also significantly shorter with high incentives (271 ± 11 ms) compared to no incentives (281 ± 11 ms) (main effect of reward F(2,76) = 5.30, p = 0.007; Figure 6B). In addition, reward reduced saccadic endpoint variability (F(1,77) = 5.02, p = 0.027; Figure 6C). It also improved accuracy by reducing oculomotor (distractor) capture rate (arcsine-transformed F(2,76) = 3.8; p = 0.026; Figure 6D). The results for accuracy and RT are re-plotted in Figure 6E, showing clearly that reward pushes performance beyond the speed-accuracy trade-off, consistent with our model predictions. Further analysis revealed that the velocity increase could not be explained by larger amplitudes or reduced curvature (see Supplemental Experimental Procedures). A conditional accuracy function plot demonstrated that the earliest responses (around 200 ms) were prone to distraction (50%), whereas later responses were more accurate, as predicted by standard speed-accuracy trade-off (Figure 6F). However, reward shifted the curve upward and leftward, as predicted by applying a control cost to simulations of the drift-diffusion model (Figure S1B; Supplemental Experimental Procedures).

Across healthy participants, those who had the greatest increase in velocity also had the greatest decrease in motor variability, indicating stronger motivational effects (r^2^ = 0.23, p = 0.001; Figure S4A). Faster individuals were also more precise (r^2^ = 0.094, p = 0.045; Figure S4B). This is predicted by the model (Figure 4C, left panels), in that a participant with low control cost σ will be both fast and precise. Participants with faster velocities were also more sensitive to reward (r^2^ = 0.12, p = 0.021; Figure S4C), which is also predicted by the model (Figure 4C, lower panels): an individual with higher temporal discount rate *k* or lower noise σ would have both a higher overall velocity and a steeper slope of velocity with reward. Interestingly, there was no correlation between reward’s effects on velocity and RT, or between reward effects on motor endpoint variability and distraction error rates (Figures S4D and S4E), suggesting that cognitive and motor control costs might be optimized independently, in keeping with our two separate model formulations.

#### Cost of Control in Parkinson’s Disease

**Figure 7:**
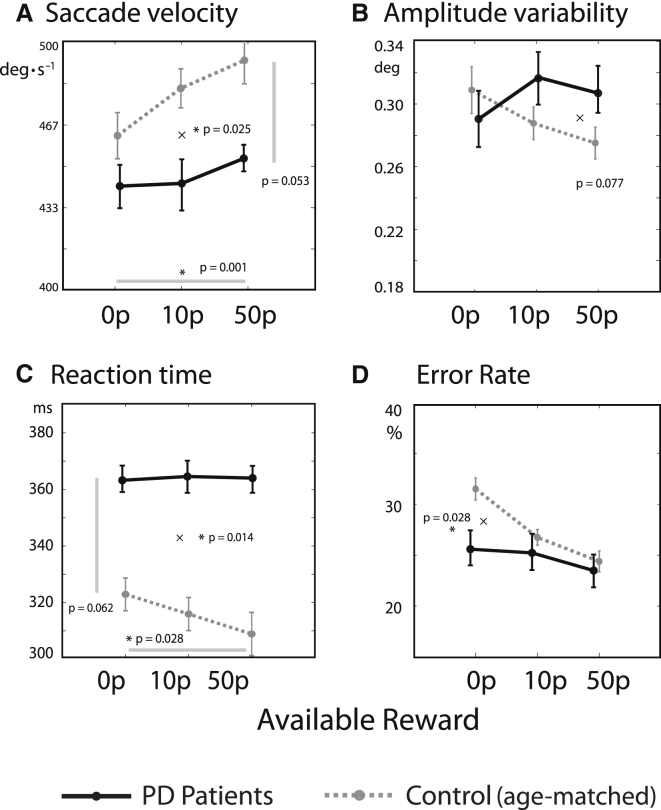
Reduced Reward Sensitivity in Patients with Parkinson’s Disease (A) PD patients had decreased reward sensitivity, as demonstrated by a shallower slope. This is consistent with impaired invigoration by reward. Overall velocities were also marginally slower. (C) Saccadic amplitude variability was not significantly abnormal in PD. (B) Reaction times were slower in PD and showed reduced reward sensitivity. (D) Patients showed weaker effects of reward on improving distractibility, as measured by oculomotor capture (i.e., they did not reduce their error rate in response to incentive), compared to controls. Between-subject correlations are shown in Figure S4.

To study the effect of dopaminergic dysfunction on motivation by reward, we compared 19 PD patients with 22 age-matched controls (Table S2), performing the same task as above (Figure 5). Patients had mild to moderate PD with no or minimal cognitive impairment. To compare patients and controls, we used a mixed-effects linear model, with factors disease and reward. There were no significant main effects of PD: patients had saccade velocities comparable to those of healthy age-matched control participants (Figure 7; PD versus control, F(1,80) = 1.18, p > 0.05) and did not make more oculomotor capture errors than controls (mean 24.8% errors in PD compared to 27.7% in controls, F(1,80) = 0.29, p > 0.05). There was a trend toward longer RTs than controls (364 ± 98 ms [SD], compared to 315 ± 66 ms for controls, F(1,80) = 3.67, p = 0.063). Critically, patients had shallower reward sensitivity slopes for velocity, RT, and error rate (interaction of disease × reward: F(1,80) = 5.19, p = 0.025 for velocity; F(1,80) = 6.32, p = 0.014 for RT; F(1,80) = 4.98, p = 0.028 for error rate), with a similar trend for endpoint variability (F(1,80) = 0.32, p = 0.077). These latter findings are consistent with reduced reward sensitivity in PD, as predicted by the precision-cost model, if the precision cost σ were increased (Figure 4E, compare red and blue lines). An analysis of just the PD group showed that patients increased their velocity significantly in response to reward (significant proportional change in velocity with reward, F(1,37) = 5.39, p = 0.026) and thus did modulate their behavior to some extent, although not to the degree of healthy controls. PD patients were not significantly influenced by reward, however, in terms of endpoint variability, RT, and oculomotor capture (all p > 0.05).

Because the model predicts that reward can either increase or decrease endpoint variability according to the individual, we performed a supplementary analysis of per-subject effects of reward (Figure S4A). Individual patients showed significant reward effects in different directions, and that effect of endpoint variability was correlated with baseline velocity, in line with the model (Figure S4B). Conditional accuracy functions also demonstrated absent reward effects in PD patients (p > 0.05) (Figure S1A). The effect of reward on RT was examined at different time points during the RT distribution (Figure S1C). In controls, responses occurring later in the RT distribution were the ones whose speed was increased the most by reward, as predicted by the simulation (Figure S1D), effects that were absent in PD. Fatigue over time could not explain the reduced reward sensitivity in PD (see Supplemental Experimental Procedures).

#### Cost of Control Explains Movement Velocity and Endpoint Variability

For each participant, velocity and variability as a function of reward were fitted to the motor control model, giving three free parameters for each subject: the temporal discount rate *k*, the noise-control cost σ, and baseline noise σ_0_. These three parameters determine the optimum velocity $\left(\sqrt{u_{F}}\right)$ and variability $\left(\sigma_{0}+\sigma u_{F}/\sqrt{u_{P}}\right)$ as a function of reward (Figure 3).

Compared to controls, PD patients had significantly increased noise-control costs σ (two-tailed unpaired t test, t(36) = 2.21, p = 0.034; Table S1). Neither their temporal discount rate nor their baseline variability was significantly different from healthy people (p > 0.05). One interpretation of the data is that PD patients go slower in order to reduce their motor variability in the face of an increased cost for controlling internal noise. The cost-of-control model fitted the data better than simpler models in which only the force or precision were allowed to vary with reward (ΔAIC = 6.5; Table S3; “Model Comparison” in Supplemental Experimental Procedures).

## Discussion

Standard optimal control theory constrains human performance to be bounded by an upper limit. Motivation by reward is remarkable for improving performance beyond its normal bounds. To account for this, we devised a variant of optimal control theory that incorporates a precision signal that allows noise to be attenuated. But importantly, precision comes at a cost—the cost of control (Figure 1). In the motor domain, our model predicts that reward may improve both velocity and precision (Figure 3). In the decision domain, it predicts faster and more accurate choices with higher reward (Figure 4).

We tested this using a novel incentivized saccadic task (Figure 5). In accordance with our model, reward increased saccadic velocity and endpoint accuracy, and reduced RTs and oculomotor distractibility (Figure 6). By allowing each participant to optimize behavior according to their own noise and temporal discounting, the model was able to accommodate individual differences in responses to reward across the populations, better than simpler models.

Applying optimality to reward incentivization unites recent conceptions of motivation [20, 24] with existing mathematical frameworks of optimal action [8, 11, 42]. If reward is held fixed, our model reduces to previous accounts [10, 36], but if reward is altered, parallel shifts can occur that violate the classical speed-accuracy trade-off (Figure 1C), at least when signal-to-noise ratios are high and temporal discounting is small (Figures 3C and 4E). Such effects are often reported as attentional improvements in cognitive control tasks [5, 6] but have not previously been quantified in terms of cost-benefit analysis.

Previous presentations of the drift-diffusion model have incorporated speeding up of decisions by reward [42], but our addition of a control cost makes new predictions for the drift rate. Neuronal ramping activity preceding a decision has been interpreted in terms of drift diffusion, but existing models fail to capture how emphasizing speed over accuracy may increase the peak firing rates at the moment of decision [43]. Unlike previous attempts, our model does predict faster RTs accompanied by higher thresholds, under specific circumstances (Figures 4C and 4E).

In both animals and humans, dopamine is considered to have a crucial role in mediating response vigor [11, 32] and in overcoming internal costs associated with particular behaviors [29]. Individuals with PD, a condition associated with dopamine depletion, had reduced sensitivity to reward on speed measures compared to age-matched controls (Figure 7), yet they maintained similar overall levels of accuracy. In the model, this corresponded to a greater cost of controlling noise. The results are in line with previous evidence that, for a matched speed, PD patients’ movements are less accurate [44] and cognitive control errors more frequent [45].

Could the loss of reward sensitivity in PD be explained simply by patients performing at their ceiling? This seems unlikely. First, the PD patients were not entirely unresponsive to reward. Second, at fast RTs PD patients are in fact *more* accurate than controls; however, at slower RTs the accuracy plateaus lower (Figure S1A). This suggests that instead of being uniformly slow, patients maintain a stable accuracy level at the cost of speed [46]. Finally, in PD, reward speeded up slow responses similarly to fast responses (Figure S1C), whereas with ceiling effects, slow saccades might be expected to show greater motivational improvement.

Although dopaminergic reward signals are well characterized, their role in weighing costs against benefits remains obscure. Our results are suggestive, but not conclusive, that dopamine depletion may lead to a higher cost of control. Dopamine might facilitate motivational performance adjustments due to its neuromodulatory effects on synaptic noise or gain [47], potentially reducing the cost of control. However, from this study alone, it is not possible to determine for certain which specific mechanisms mediated the effects we observed. Although our patients had mild to moderate PD without dementia, we cannot rule out pathology in non-dopaminergic systems.

### What Is the Real Cost of Reducing Noise?

If control signals *can* truly attenuate noise, then why are we not built to exercise maximal control at all times? There are at least three possible reasons why control should be expensive: opportunity costs, neural resources, and entropy.

First, the “noise” that needs to be attenuated in the brain might in fact be constituted by *potentially* relevant but currently irrelevant signals. Distraction confers ecological advantages, and ignoring distractors could be costly or dangerous. For the motor system, analogously, producing precise movements entails isolating the motor system from competing affordances. Selective attention and precision thus carry danger or opportunity costs. Second, controlling noise might require allocation of more “neural resources,” for example more neurons in population codes [23], higher firing rates (Figure S6), or the reduction of motor error by co-contraction of antagonistic muscles, which increases effector stiffness but incurs an energetic cost. Finally, any feedback-control signal that maintains stability in the face of thermal noise will inherently increase the entropy of a system [48], which must be dissipated as heat [49]. Consequently, minimizing control signals may be a central principle of brain design.

Whatever the real cost of control, its estimation and optimization by the brain can be summarized by the equations presented here. Cost-benefit optimization then directly predicts the observed effects of reward on speed and accuracy. We suggest that the mathematical formulations of optimal control theory, complemented by our costly noise-reduction signal, would be broadly applicable to any domain in which behavioral performance is limited by neuronal noise or resources. If combined with an appropriate model of how noise degrades performance, our formulation might also predict motivation’s effects on more complex aspects of behavior, such as attentional selection, working memory, and inhibitory control.

## Experimental Procedures

### Application of Precision Cost to Motor Commands

Equation 1 indicates the considerations in evaluating an action. To express *P*_*win*_ and temporal discounting *D* as a function of the control command **u**, we first assume hyperbolic temporal discounting, *D*(**u**) = 1/(1 + *kT*(**u**)) [12]. Second, accuracy *P*_*win*_(**u**) depends on the amount of motor noise, which is usually assumed to be Gaussian, and proportional to |**u**| [26]. Reward will be missed if noise exceeds some threshold. The probability of landing within a unit radius is given by the cumulative normal error function (denoted Φ). This gives an equation for the “orthodox view” (Figure 1B),

$$EV\left(u\right)\text{∝}\frac{R}{1+k\cdot T\left(u\right)}2\text{Φ}\left(\frac{1}{\sigma \cdot \left|u\right|}\right)-{\left|u\right|}^{2},$$

where the parameter *k* indicates a subject’s temporal discount rate and σ denotes their motor noise.

For a simple one-dimensional movement, we find the optimal force/precision pair **u** = [*u*_F_, *u*_P_]. We assume that noise is scaled down by precision, and for our specific motor task, we write the noise as $\sigma u_{F}/\sqrt{u_{P}}$ (see Supplemental Experimental Procedures). Furthermore, movement time depends on the force component of the command, with $T\left(u\right)\text{∝}1/\sqrt{u_{\text{F}}}$. Finally, since we treat *u*_P_ as a control signal, it contributes to the cost |**u**|^2^, alongside the force. This gives the expected value (EV) of a command (Figure 1D):

$$EV\left(u_{F},u_{P}\right)\text{∝}\frac{R}{\mathrm{1+k}/\sqrt{u_{F}}}2\text{Φ}\left(\frac{\sqrt{u_{P}}}{\sigma \cdot u_{F}}\right)-{\left|u_{F}\right|}^{2}-{\left|u_{P}\right|}^{2}.$$

### Application of Noise-Reduction Cost to Cognitive Control: Drift-Diffusion Model

The drift-diffusion model allows us to predict the RT distribution and error rate of a two-alternative choice. The outcome of the decision depends on the average rate of accumulating information μ, the threshold θ at which enough information is available to make a decision, and σ, the amount of noise in the accumulator (Figure 4A). We suggest that an organism can control not only the threshold, but also decision noise, to optimize EV. Noise can be reduced by a precision signal to give an effective noise level $\sigma /\sqrt{u_{P}}$. This precision entails a cost |**u**|^2^ = *u*^2^_*P*_. In an alternative race model framework, the rate of rise might be increased (Figures S2 and S3).

The time taken (*T* = RT) and accuracy *P*_*win*_ are calculated by simulating the diffusion process. We assume hyperbolic temporal discounting of reward, with *D*(θ, *u*_P_) = 1/(1 + *kT*). These values are substituted into Equation 1. The optimum threshold and precision [θ, *u*_P_] can then be found by simulation, which in turn determine speed and accuracy (Figures 4B–4E). Performance therefore depends on the reward on offer, the individual’s baseline signal-to-noise ratio σ, and their temporal discount rate. High reward emphasizes time pressure but also encourages investment in precision—enabling the classical speed-accuracy trade-off to be broken by motivation.

## Author Contributions

S.G.M. designed, ran, and analyzed the model and experiments. T.T.-J.C., A.B., M.S., K.P.B., P.R.J., and M.H. recruited patients for the study. S.G.M, M.H., and M.A.J.A. wrote the manuscript.

## Supplemental Information

Document S1. Figures S1–S6, Tables S1–S3, and Supplemental Experimental Procedures

Document S2. Article plus Supplemental Information
