# Supplementary material for: Reward Pays the Cost of Noise Reduction in Motor and Cognitive Control
Source: Curr Biol. 2015 Jun 29;25(13):1707–16. doi: 10.1016/j.cub.2015.05.038 (PMC4557747; doi:10.1016/j.cub.2015.05.038)
Supplement: Document S1. Figures S1–S6, Tables S1–S3, and Supplemental Experimental Procedures [file mmc1.pdf]

Current Biology

Supplemental Information

Reward Pays the Cost of Noise Reduction in Motor and Cognitive Control

Sanjay G. Manohar, Trevor T.-J. Chong, Matthew A.J. Apps, Amit Batla, Maria Stamelou, Paul R. Jarman, Kailash P. Bhatia, and Masud Husain,

Supplemental Figures and Tables

Figure S1. The time course of reward's effects (related to Fig.6F)
Figure S2. Race model interpretation of data (related to Fig.6D)
Figure S3. Analysis of time to correct an error (related to Fig.6C)
Figure S4. Correlation between reward sensitivity measures in healthy participants (related to Fig.6A)
Figure S5. Effects of internal noise and temporal discount rate on velocity and motor precision (related to Fig.3C)
Figure S6. How much negative feedback signal is required to attenuate noise? (related to Fig.1D)

Table S1. Fitted model parameters for healthy participants and patients
Table S2. Demographics of participants
Table S3. Model comparison for the drift-diffusion model fit

Supplemental Experimental Procedures

Modeling the cost of control

1 .Previous approaches predict invigoration by reward
2. Feedback control signals could attenuate internal noise
3. Optimisation with a novel precision signal
4. How much does noise reduction cost?
5. Applying the cost of control to saccades
6. Drift diffusion simulation

Empirical quantification of reward's effect on speed and accuracy

1. Task Instructions
2. Task
3. Materials
4. Saccade analysis
5. PD Patients
6. Statistics
7. Velocity effects were not attributable to amplitude or curvature
8. Conditional accuracy functions and Delta plots
9. Fatigue could not explain reduced reward sensitivity in PD
10. Analysis of distractor-target delay and error trials

Model fit of reward's effects on motor control

1. Model comparison
2. Patient vs. control comparison

Supplemental References

Figure S1

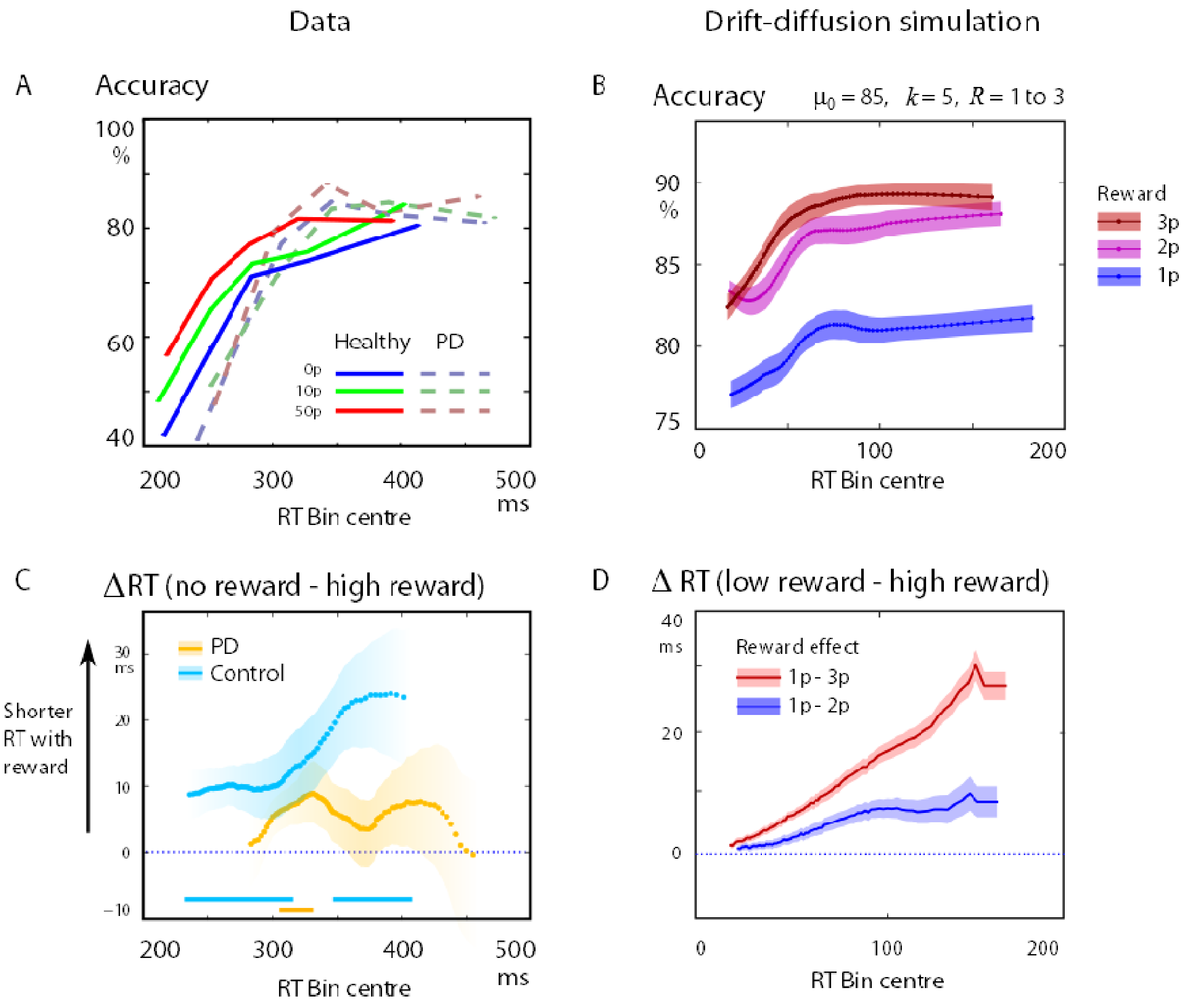

Figure S1: The timecourse of reward's effects (related to Fig.6F)

A) Conditional accuracy functions were constructed by binning reaction time into quintiles, and calculating the proportion of non-captured saccades in that bin, for each participant, and averaging across individuals. PD patients show a reduced reward effect, compared to age-matched controls, despite being more accurate overall. Shaded areas represent the standard error across individuals.

B) A drift-diffusion model was simulated to generate RT distributions for erroneous and correct responses. Those RTs can be plotted in the same way as data in panel **A**. Incorporating a cost for controlling noise in the accumulator allows the effect of reward to be predicted. Higher incentives lead to higher thresholds, but also higher signal-to-noise ratios. This results in a conditional accuracy function that shows an upward-and-leftward shift with incentive, similar to the data. Simulated subject parameters: signal = 85, baseline noise = 0.1, starting value variability 0.3, timestep 1 ms, discount rate 5 s^{-1}, reward 1 to 3 units, 500 trials. Threshold and precision chosen to maximise value, according to optimal control theory as described in **Supplementary methods**. Shaded area indicates standard deviation across 20 runs.

C) The effect of reward on RT was analysed for different RT bins, using a "delta plot". The plot was constructed by comparing each RT quantile in the high and low rewarded conditions, using a sliding window (See **Supplementary methods**). The curves shown are the average of (50p minus 10p) and (10p minus 0p), for each group. Healthy participants (blue) had a consistent effect of reward throughout the RT distribution, indicating that reward shortened reaction times by shifting the whole RT distribution. The effect of reward is weakest for early movements, and strongest for late movements. PD patients showed minimal effect of reward over the whole RT distribution. Bars below indicate $p<0.05$ by permutation test.

D) Simulated reaction times can be plotted to show the effect of reward as a function of RT, in the same way as data in panel **C**. The plots show that the effect of reward on RT is predicted to be largest on later responses, similar to the data.

Figure S2

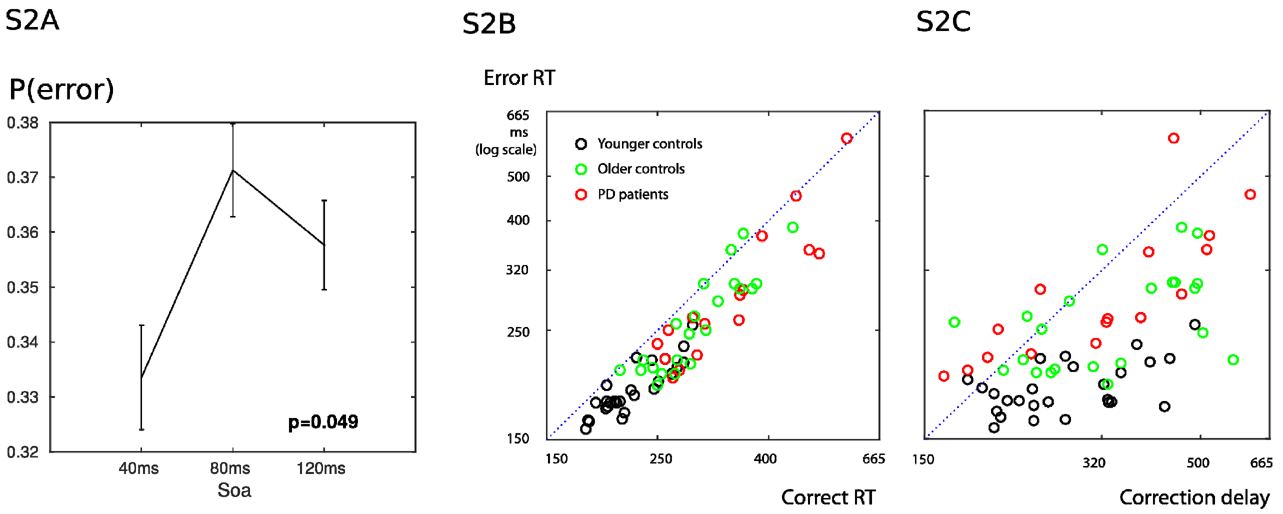

Figure S2: Race model interpretation of data (related to Fig.6D)

A) The interval between the distractor onset and the target onset (the SOA) was 40, 80 or 120 ms. The chance of a saccade to the distractor varied with SOA, compared to 40 ms (repeated measures 1-way ANOVA, main effect of SOA, $F(2,52)=3.20$, $p=0.049$; pairwise comparison 40 ms *vs*. 80 ms, $t=2.34$, $p=0.027$)

B) Error saccades to the distractor had a shorter latency than saccades that went directly to the target. For each participant we plotted the mean RT on error trials, against the mean RT on correct trials. On average, error RTs were shorter in all three groups (all $p<0.001$).

C) Error corrections occurred after each error saccade, as participants were required to look at the target before progressing to the next trial. The "correction delay" was taken to be the interval between the initiation of the error and the initiation of the first subsequent corrective saccade which landed on the target. Participants with longer error RTs tended to take longer to correct their errors (younger: $r^2=0.29$, $p=0.004$; older $r^2=0.64$, $p<0.001$; PD $r^2=0.22$, $p=0.024$). Although error RTs were much faster for younger controls compared to older controls and patients, there were no differences between groups in the correction delay.

Figure S3

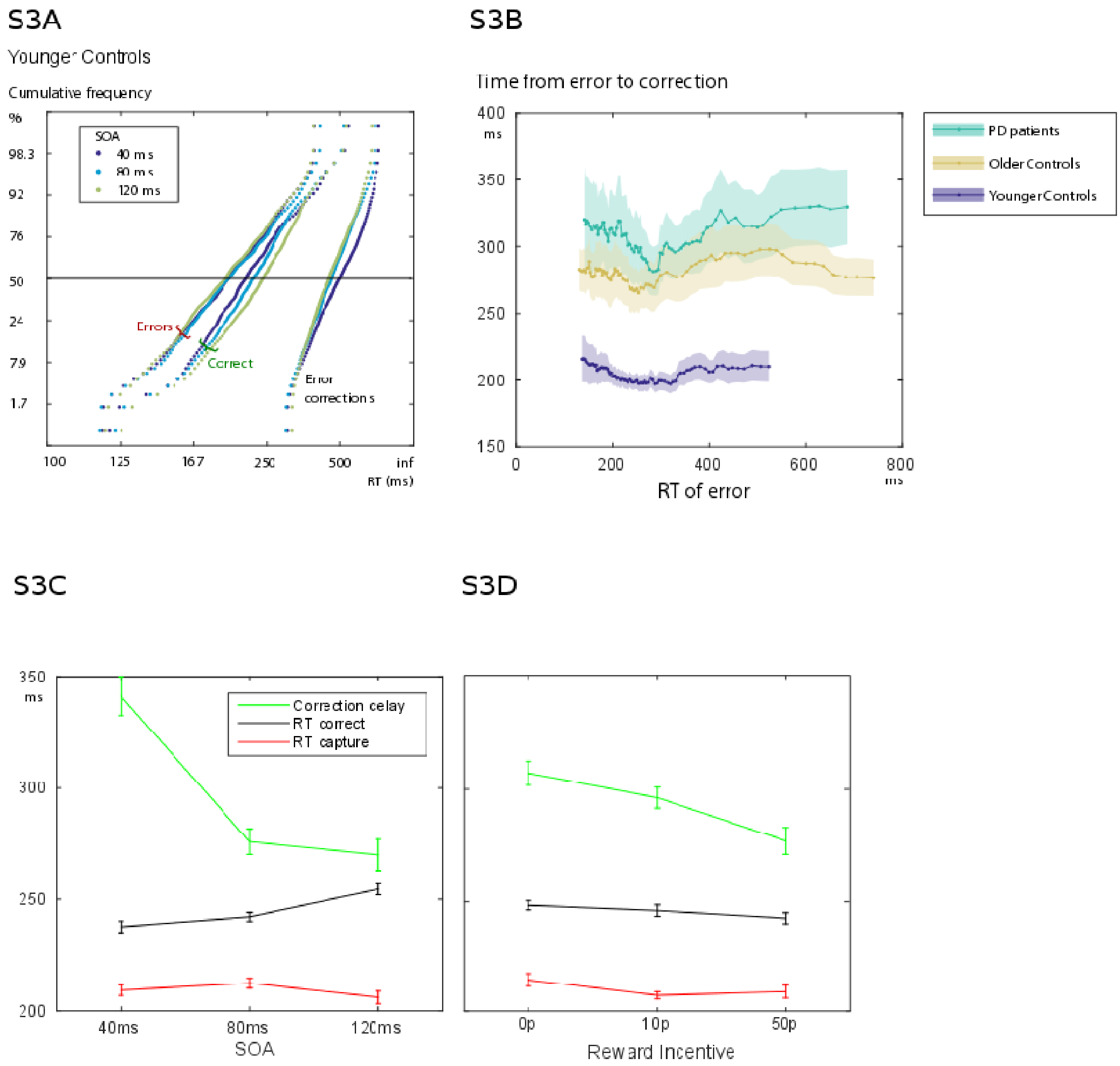

Figure S3: Analysis of time to correct an error (related to Fig.6C)

A) Cumulative histogram of reaction times for the younger 17 control participants. All times are relative to the onset of the distractor. For error trials, both the error RTs (far left) and times of the corrective saccade (far right) are plotted. For each participant, the RTs were quantiled, and proportion of saccades exceeding each percentile was plotted. The grand average across participants is plotted with one point per percentile bin; the axes are "reciprobit transformed" (Carpenter and Williams 1995) such that if 1/RT is normally distributed, the cumulative distribution appears as a straight line.

B) Relationship between time of error and time to correct the error. Previous studies have found that faster errors take longer to correct, and this has been taken to be evidence for a "race" between competing motor plans toward the distractor and target. To test this, for each subject, the joint distribution of error responses and corresponding error-corrections was examined using a sliding window. For errors that fell within each 20% quantile window, the mean of the corresponding error-correction delays were plotted. The error correction delay is the time interval from the initiation of the first saccade that landed on the distractor, to initiation of the first saccade that landed on the correct target. Within each group, there was no significant

effect of error RT on correction time. Error bars indicate standard error of the mean correction delay across individuals. N=25 for the younger controls, N=22 for older controls, and N=16 for PD patients

C) There was no effect of the distractor-target interval (SOA) on capture RTs (red). However, longer SOAs resulted in later correct responses (black), but faster error corrections (green). Error bars indicate standard error of the main effect of SOA.

D) Reward shortened RTs on correct trials, and also speeded up error correction RTs. Error bars indicate standard error of the main effect of reward.

Figure S4

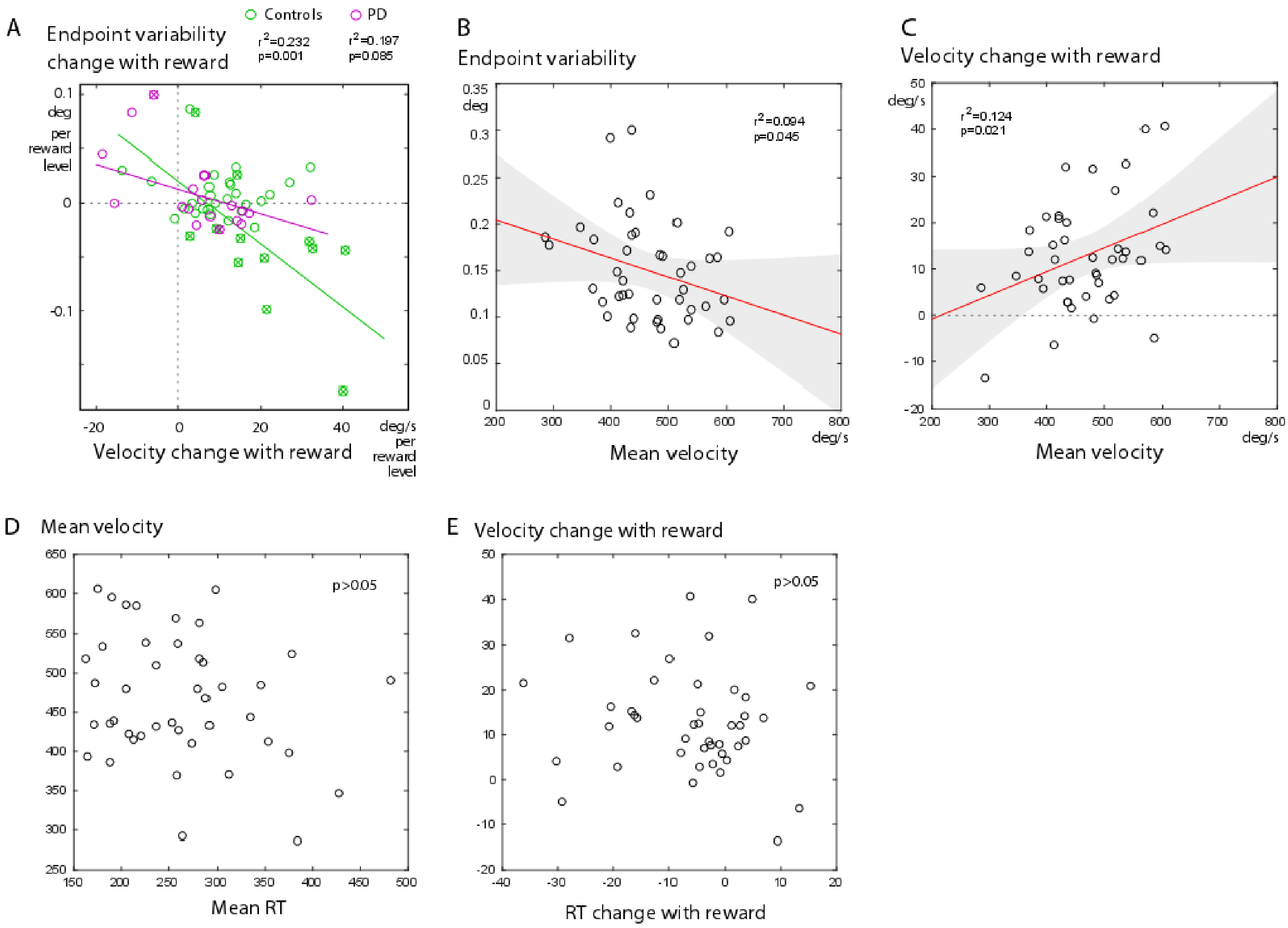

Figure S4: Correlation between reward sensitivity measures in healthy participants (related to Fig.6A)

In these plots, each subject is represented by a single point.

A) For each participant, sensitivity to reward was measured firstly for peak velocity, and secondly for amplitude variability, as a function of reward incentive. The values plotted on each axis correspond to the slope per unit reward, i.e. the degree of modulation by incentive. There was a significant negative correlation both in 39 healthy controls and in the PD patients, indicating that individuals who *increased their velocity* for high incentives also *decreased their endpoint variability*.

Our model predicts that endpoint variability may either increase or decrease with reward. Since there was no significant interaction between groups in the amount they modulated endpoint variability with varying reward, we asked whether individual participants had significant reward effects (as described in **Supplementary Methods**, section titled Statistics). Symbols filled with an 'x' indicate individuals who showed reward effects.

B) The overall endpoint variability for each individual was plotted against their overall mean velocity. There was a significant correlation indicating that participants who had faster saccade velocities also had more precise endpoint distributions. This effect is predicted from the model (Fig. **2C** left panels), where a participant with lower noise would be faster and less variable.

C) The size of the velocity change induced by reward was plotted against overall velocity for each participant. There was a significant positive correlation, indicating that faster individuals were able to increase their speed more. This is also predicted from the model (**Fig. 2C** lower panels), in which either a lower noise level or steeper temporal discounting would both lead to higher speeds but also stronger effects of reward on speed.

D) There was no correlation between a participant's mean RT and their mean peak saccade velocity ($r^2<0.1$, $p>0.05$).

E) There was also no correlation between the degree to which reward's influence on RT, and its influence on saccade velocity ($p>0.05$). Taken within the correlations between velocity and variability, and the correlation between reward sensitivity of velocity and variability (panels **A-C**), the lack of correlation between decision speed and movement speed suggests that decision and movement parameters may be optimised independently.

Figure S5

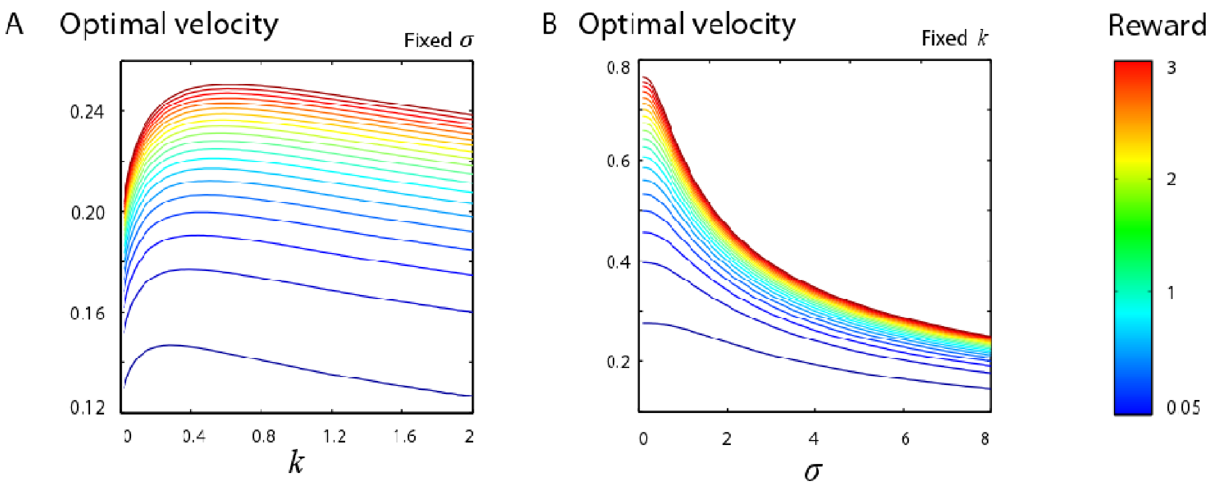

Figure S5: Effects of internal noise and temporal discount rate on velocity and motor precision (related to Fig.3C)

These figures show the same effects as in **Fig. 2C**, but with the model parameters on the x-axis. The values k and σ are fixed for an individual subject, but these plots allow us to examine how they influence behaviour. This provides a better visualisation of how temporal discount rate k and the difficulty of a task for an individual would alter their optimal movement velocity, and endpoint variability.

A) The optimal velocity of a movement initially increases with steeper temporal discounting. This is because if the value of a reward increases a lot with a small reduction in movement speed (i.e. very negative $\frac{d(EV)}{dT}$, for a given movement time T), then it pays to invest in speed. If the temporal discount rate becomes very high, then normal movement times fall in a flatter portion of the temporal discount curve. In other words, since reward has been discounted very heavily by time, a small change in speed will not affect the value much. Note that reward always increases the optimal velocity.

B) As the noise parameter σ increases, speeds are reduced. The level of noise may be due to individual differences, as well as task difficulty.

Figure S6

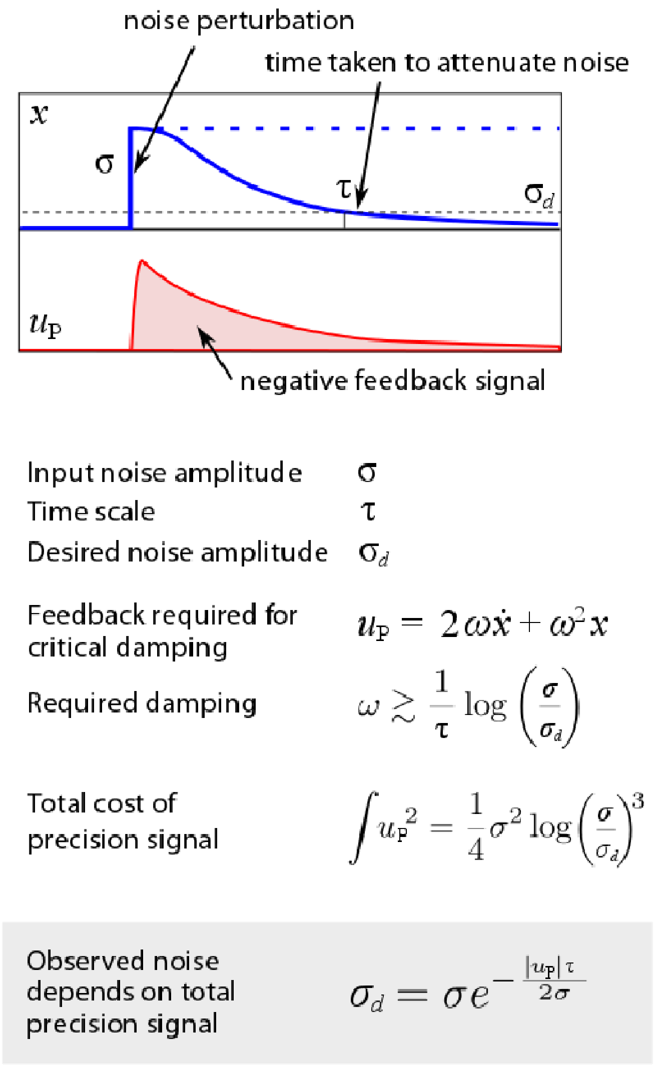

Figure S6: How much negative feedback signal is required to attenuate noise? (related to Fig.1D)

Here we illustrate that it is possible to find a mathematical relationship between the size of feedback signals, and the reduction of noise.

We consider a second-order dynamic system in which control signals can be used to restore a system to its baseline state. The fastest way to accomplish this is to use critical damping. The "restoring force" depends on the size of the signal and its current rate of change, according to the equation $\ddot{x} + 2\omega\dot{x} + \omega^2 x = 0$. If the system deviates from its stable state, for example by an amount σ, then the restoring force will gradually return it to that state. After a time τ, the disturbance will have been attenuated to a lower amplitude. Let the desired level be σ_d after time τ. We calculate the total amount of negative feedback signal that will need to be expended, in such a system, to reduce the disturbance to a desired level. The final equation shows with a higher size of feedback signal u_P , we obtain a more quickly responsive system, and for a given time scale, the resultant noise σ_d is reduced. The equation also shows that if $\sigma \ll |u_P|\tau$ the observed noise is close to zero, but when $\sigma \gg |u_P|\tau$, there is a constant subtractive attenuation of observed noise, with $\sigma_d \approx \sigma - \frac{1}{1}|u_P|\,\tau$. This final result is reported in the **Supplementary experimental procedures** section, "The cost of noise reduction".

The purpose of this derivation is to demonstrate that if signals are costly, for example in terms of neuronal firing rates, then we can put a price on cancelling out noise.

Table S1

	**Temporal discount rate** k	**Motor noise** σ	**Baseline variability** σ_0
**Healthy controls**	0.22 (0.03)	0.68 (0.08)	0.058 (0.028)
**Parkinson's disease**	0.17 (0.29)	0.94 (0.11) *	0.102 (0.032)

Table S1: Fitted model parameters for healthy participants and patients

Estimates for the three fitted parameters shown as medians across individuals (with standard error). PD patients had significantly higher motor noise σ compared to age-matched healthy control participants. Other parameters did not differ significantly. This is consistent, in our model, with an increased cost for controlling motor noise in PD.

Table S2

Healthy participants (n=39)		
	Mean	SD
Age	46.2	19.7
Sex	28 M	12 F
**PD Patients (n=19)**		
Age	65.3	9.0
UPDRS	23.1	10.1
Hoehn and Yahr Stage	1.8	0.86
Schwab and England daily living scale	86%	14%
Levodopa equivalent dose	507	240
HADS Depression score	3.6	3.0
Sex	9 M	10 F
**Matched Controls (subset of healthy participants, n=22)**		
Age	62.5	8.9
Sex	10 M	12 F

Table S2: Demographics of participants

Forty healthy volunteers were recruited from a public advert and UCL psychology subject pool. All participants had normal or corrected-to-normal vision. Data from one participant was corrupted on disk and was not analysable.

Nineteen patients with mild-to-moderate Parkinson's disease were recruited from the neurology clinic at the National Hospital for Neurology and Neurosurgery at Queen Square, UCL London. Ten patients were taking dopamine agonists (6 ropinirole, 3 pramipexole, 1 rotigotine), and 15 were taking levodopa. The levodopa equivalent dose was calculated from standard conversions [S1]. One patient was taking amitriptyline at night for sleep problems. There was no significant difference in age between patients and controls ($t(38)=1.01$, $p>0.05$).

Table S3

Model	1: Full model	2: Speed-accuracy trade-off only	3: Motivation only
**Parameters**	u_F and u_P vary with reward.<br>k, σ, σ_0 fixed for each subject	u_F varies with reward.<br>k, σ, σ_0 and u_P fixed for each subject	u_P varies with reward.<br>k, σ, σ_0 and u_F fixed for each subject
**AIC**	-206.6	-200.1	-154.6

Table S3: Model comparison for the drift-diffusion model fit

Three models were fitted to the data. The full model optimises both the force and precision for each reward level, to optimise expected value, predicting the velocity and endpoint variability. Model 2 keeps precision fixed and allows force to vary with reward, as in the standard speed-accuracy trade-off model—and thus predicts greater variability with reward. Model 3 keeps force fixed whereas precision can vary with reward. Model 1 fits significantly better than both of the simpler models.

Supplemental Experimental Procedures

Modelling the cost of control

1. Previous approaches predict invigoration by reward

Two previous approaches both predict that reward increases urgency. On one account, the organism expects an average ongoing reward rate. Movement duration and reaction time (RT) therefore count as a costly wasted time. If the ongoing expected reward rate is denoted by $\bar{R}^*$, then waiting a time T wastes reward $\bar{R}^* \cdot T$. Niv et al. (2005) showed that, if fast actions carry a cost proportional to $1/T$, then expected value (EV) of an action is given by

$$EV = \text{constant} - \bar{R}^* \cdot T - \frac{\epsilon}{T} \tag{3}$$

where ϵ is an effort cost. High rewards therefore lead to faster optimal movement times, given by

$$T^* \propto \frac{1}{\sqrt{\bar{R}^*}} \tag{4}$$

Optimal movement time = 1 / √(expected optimal ongoing reward rate)

An alternative formulation of time pressure is that delayed rewards are worth less. *Temporal discounting* may be modelled as an exponential or hyperbolic decay, such that the subjective value of a reward is higher if it is obtained earlier. A highly simplified form of the cost functions used by Rigoux and Guigon [S2] and Shadmehr et al. [S3] gives the value of an action as

$$EV = \frac{R}{1+kT} - \frac{\epsilon}{T} \tag{5}$$

where k denotes temporal discount rate. This results in an optimal movement time T^* (**Fig.2A**):

$$T^* \propto \frac{\sqrt{R} + \sqrt{\epsilon k}}{R + \epsilon k} \tag{6}$$

These two formulations are in fact equivalent if $\epsilon k \ll R$, both predicting invigoration by incentives, with movement speed approximately proportional to $1/\sqrt{R}$.

On the other hand, there may be two reasons to favour *slower* movements. To see this, we denote the motor command by **u.** The vector **u** is a set of instructions varying over time, that represents, for example, the neural output to different muscles. Faster movements require larger motor commands **u**. First, speed carries an energetic cost, often taken to depend on the squared magnitude of **u**, that is, the sum of squares of all individual control commands. Second, motor commands are subject to signal-dependent noise, so faster movements suffer from greater variability. So to weigh up speed advantages with speed costs, we include the probability of landing on the target P_{win}, which falls with increasing motor noise. We obtain:

$$EV(\mathbf{u}) = R \times D(\mathbf{u}) \times P_{win}(\mathbf{u}) - |\mathbf{u}|^2 \tag{7}$$

Expected value of action = Reward × temporal discount × probability of reward given a motor command – (size of motor command)^2

where D is the temporal discount factor. There is an optimal value of **u** that balances the need to go

fast (maximise *D*) and accurately (maximise P) at low expense (minimise $|\mathbf{u}|^2$).

2. Feedback control signals could attenuate internal noise

Optimal motor control describes how control signals $\mathbf{u}(t)$ should be chosen in order to maximise a value function. The control signals alter the state of the body/world $\mathbf{x}$. However, the system's actual output is stochastic[S4], so $\mathbf{x}$ does not always track its desired trajectory[S5, 6]. Control signals can use sensory feedback to correct for perturbations in $\mathbf{x}$ due to noise. The optimal way of doing this is by computing an *internal estimate* of the world's state, $\hat{\mathbf{x}}$, and applying a Kalman gain to the difference between the observed and expected states of the system. [S7]. In effect, motor noise can be counteracted by feedback signals [S6].

Interestingly, this error-correcting feedback can be applied not only to motor noise, but also to *internal* noise [S8, 9]. The command $\mathbf{u}$ may include "internal" signals, which influence the internal state of the organism, rather than immediate motor output [S10, 11, 6, 12]. In this case $\hat{\mathbf{x}}$ contains not only representations of the world's state, but also internal state. The internal state estimates, corrupted by noise, can be steered back towards their desired states using the internal components of control signals. In general, the transformations at one hierarchical level of the motor system can generate error signals for a higher level [S13]. By including higher-level, non-motor commands or "predictions" in $\mathbf{u}$, in addition to muscle-level output commands[S14], internal noise-compensation could account for precision improvements in ballistic movements, when no external feedback is available.

To make this transparent, we allow the command to have two components—one for the standard motor command, $\mathbf{u}_F$, and another that represents the *precision* signals, i.e. the degree to which motor noise is attenuated, $\mathbf{u}_P$. The notion of a precision signal allows us to remain agnostic about the actual signals that attenuate noise, but asserts that they are costly. The larger the precision command $\mathbf{u}_P$, the lower the effective amount of noise in the force generated by $\mathbf{u}_F$, but the higher the cost. A particular choice of control command may therefore encapsulate not only the force of the movement, but also the precision.

3. Optimisation with a novel precision signal

We consider a simplified, single-command model where both force u_F and the novel precision control signal u_P, are just single scalar numbers. Optimal choice of action involves selecting a precision/force pair, $\mathbf{u}=[u_P, u_F]$, which minimises $|\mathbf{u}|^2$ and maximises expected reward value:

$$EV(u_P, u_F) = R \times D(u_F) \times P_{win}(u_F, u_P) - u_P^2 - u_F^2 \tag{8}$$

where the discount rate *D* is a decelerating positive monotonic increasing function of the force u_F, and the chance of success P_{win} decreases with force but increases with precision. The general optimal solution for $\mathbf{u}$ is given by a pair of differential equations:

$$u_F^* = \frac{1}{2}R\frac{\partial}{\partial u_F}(P_{win}(u_F, u_P)D(u_f)) \tag{9}$$

$$u_P^* = \frac{1}{2}R \cdot D(u_F)\frac{\partial P_{win}(u_F, u_P)}{\partial u_P}. \tag{10}$$

Given expressions for $P_{win}(u_F, u_P)$ and $D(u_F)$, equations (9) and (10) can be numerically solved for the optimal motor command.

4. How much does noise reduction cost?

It may be possible to relate the size of a precision command to reductions in noise amplitude. We

consider two possible mechanisms: firstly, a *negative feedback* signal to attenuate noise, and secondly, duplication of a signal over *many independent channels*, to average out the noise.

In the first case, consider a signal that is perturbed by noise (or deflected by an irrelevant input) of a given size. We can ask: how large a control signal is required to annul this perturbation? For a second-order control system with critical damping, we can calculate the total amount of control signal (the "restoring force") required to return the system to its desired state. After the perturbation, a control signal of amplitude u_P can lead to a disturbance σ being reduced to $\sigma e^{-\frac{u_P \tau}{2\sigma}} \approx \sigma - \frac{1}{2} u_P \tau$ over a given timescale τ (**Fig. S6**). If control signals u_P are costly in virtue of their amplitude, then we have a relationship between the size of the noise perturbation σ, and the cost for attenuating it within a given time.

In the second case, consider a signal that is duplicated in a number of channels, and that each channel is independently corrupted by noise. Averaging the noisy channels yields an estimate of the original signal. If u_P independent channels are used, noise is reduced by a factor of $\sqrt{u_P}$[S15]. There are two costs in duplicating the signal: first there is a higher total amount of signal, proportional to u_P (e.g. more neuronal action potentials are used), and second, there is an opportunity cost (e.g. from representing a signal more widely in the brain, at the expense of other signals)[S16].

For further calculations we use the second formulation. However both accounts provide an *estimate of the cost for reducing noise*, in terms of the energy expended in the neural precision signal. In principle, any relationship in which cost increases with increasing precision, could be incorporated into our framework.

5. Applying the cost of control to saccades

Here we derive more formally the equation in **Fig.1**. To estimate the EV for a saccadic task, we must find expressions for delay discounting D, and the chance of success P_{win}, in equation (8). We employ a hyperbolic temporal discount function of the action duration[S17], $1/(1+kT)$. For P_{win}, we must estimate the probability of obtaining the reward. We assume that movements end in a Gaussian distribution around an intended target, and are only rewarded if they end within a fixed radius of a target[S2]. For instance, the distribution of endpoints may have a width proportional to the movement force signal, but inversely proportional to the precision signal. The value function has two free parameters: temporal discount rate k, and intrinsic motor noise level σ, and is proportional to:

$$EV\left(\mathbf{u}_F, \mathbf{u}_P\right) \propto \frac{R}{1+k\cdot T(\mathbf{u}_F)} 2\Phi\left(\frac{\text{precision}(\mathbf{u}_P)}{\sigma \cdot \text{force}(\mathbf{u}_F)}\right) - \left|\mathbf{u}_F\right|^2 - \left|\mathbf{u}_P\right|^2 \tag{11}$$

Expected value of action = Reward / (1+ discount rate × movement time) × probability of landing on target – cost of control signal

where Φ denotes the cumulative Gaussian error function.

To compute the time for a movement $\mathbf{u}$, we make the simplifying assumption that $\mathbf{u}(t)$ respects a constant profile scaled over time and amplitude. For constant movement amplitude, the movement time T is inversely proportional to $\sqrt{u_F}$, since the distance travelled is proportional to $\iint_0^T \mathbf{u}_\mathbf{F}\, dt\, dt$. We assume a specific movement amplitude is desired—consideration of variable amplitudes has been explored by other authors [S18–20]. Next, we take the noise to be signal-dependent, i.e. proportional to force u_F[S21], but reduced by our new precision signal by a factor $\sqrt{u_P}$ (see previous section). This gives an effective noise amplitude = $\sigma\, u_F / \sqrt{u_P}$. Substituting these into the EV equation gives:

$$EV(\mathbf{u}) \propto \frac{R}{1+k/\sqrt{u_F}} 2\Phi\left(\frac{\sqrt{u_P}}{\sigma u_F}\right) - u_F^2 - u_P^2 \tag{12}$$

where σ is a subject's baseline noise, and k their temporal discount rate. The cost of a command $|\mathbf{u}|^2 = u_F^2 + u_P^2$ represents the integral over the duration of the movement, and is scaled to match the reward units. This total $\int |\mathbf{u}|^2$ may represent the *overall effort* invested in a movement, including both physical energy, and the cost of precision control signals, i.e. the effort of overcoming noise. This quantifies the intuition that effort is not only physical, but also incorporates being precise in the face of noise.

Maximising EV predicts how the optimal force u_F and precision u_P should vary as a function of the available reward R. Numerical solutions for optimal u_F and u_P are portrayed in **Fig.3B**, as a function of reward and subject parameters k and σ. Velocity increases with reward, similar to previous formulations of vigour [S3, 22]. But critically, reward can *simultaneously* decrease movement variability. Under specific conditions, reward may also induce classical speed-accuracy trade-offs. In particular, when the signal-to-noise ratio is low, reward leads to slow and accurate choices (**Figure 4E**)[S23], and when the temporal discount rate is high, reward will lead to fast and erroneous movements (**Figure 3C**).

What does the noise parameter σ represent? Since endpoint variability is $\frac{\sigma u_F}{\sqrt{u_P}}$, σ is simply the endpoint variability of a fixed-speed movement, under fixed motivational conditions, relative to the target size—i.e. an individual's task-relevant motor noise. However, to account for the possibility that not all noise may be controllable by a system (e.g. noise in the effector itself), an additive baseline noise term σ_0 can be included. This latter term therefore represents error that would be present even in the highest motivational state. In this case, σ expresses the amount of investment required to reduce noise to a given level. It is thus the relative *cost of a precision signal*, compared to energetic (force) cost. The value 1/σ therefore signifies the signal-to-noise gain per unit of investment of reward.

An alternative way of parameterising equation (12) emphasises the cost of control. If control signals are independently scaled, with coefficients to determine their costs, equation (8) becomes:

$$EV(u_P, u_F) = R \times D(u_F) \times P_{win}(u_F, u_P) - w_F u_F^2 - w_P u_P^2 \tag{13}$$

where the ratio of the weights w_F and w_P determines the relative costs of precision and physical energy. These re-arrangement shows explicitly that σ (in Eqn.12) indicates the cost of precision. It also includes an additional degree of freedom, in which the scaling of reward is also free to vary.

6. Drift diffusion simulation

The drift diffusion model allows us to predict the reaction time distribution and error rate of a two-alternative choice decision[S24, 25]. The outcome of a decision depends primarily upon three parameters, the average rate of accumulation of information μ, the threshold θ at which enough information is available to make a decision, and σ, the amount of noise in the accumulator (**Fig. 4A**).

Improvements in decision-making are often considered to be "top-down" or "attentional" effects. We suggest that in a hierarchical control system, the inputs from a higher level can be construed as reducing noise or unwanted perturbations in the lower level (see "The cost of noise reduction", above). To implement this, we take the *signal-to-noise ratio* of an accumulator to be increased by a single scalar precision signal, which carries a cost. An organism might therefore select not only the threshold, but also the noise in the decision, in order to optimise EV. For example a precision u_P might result in an effective noise level $\sigma/\sqrt{u_P}$, at a cost $|\mathbf{u}|^2 = u_P^2$. The time taken is now the RT,

and P_{win} is the error rate, both calculated by simulating the diffusion process. These values are substituted into equation (7), and the optimum threshold and precision can then be found, which in turn determine speed and accuracy (**Fig. 4B-E**). Performance depends on the reward on offer, the individual's baseline signal-to-noise ratio μ/σ, and their temporal discount rate. High reward adds time pressure but also encourages *investment in precision*—enabling the classical speed-accuracy trade-off to be broken by motivation.

Simulations of decisions were run, finding the optimal θ and u_P to maximise EV. Each run simulated 2000 trials, and results were averaged over 10 runs. Parameters that were varied included 12 levels of reward ranging (0.25–3), 8 levels of the signal μ (1–8), and 3 levels of accumulator noise σ = 1, 2 and 3. Diffusion proceeded according to

$$\delta A = \left(\mu + \frac{\sigma}{\sqrt{u_P}}\mathcal{N}\right)\delta t; A(0) = 0 \tag{14}$$

such that the accumulator A increases or decreases with mean rate μ, perturbed by a Gaussian random variable N which is scaled by the internal noise σ, divided by the precision signal u_P. A time step of 1 ms was used, with noise amplitude σ/10 per timestep, and temporal discount rate of 0.1 s^{-1}.The RT for each trial was the first time step at which either A > θ or A < $-\theta$. For each condition, the EV was calculated (using Eqn. 7), assuming that reward that falls off over time as e^{-T^2} (similar results were obtained with e^{-kT} and $1/(1+kT)$). The precision u_P and threshold θ maximising EV was found for each condition (combinations of R and μ/σ) using a pattern search with 10 random starting points for each condition. This method takes into account the shape of the RT distribution. The cost of one unit of precision was scaled to 70 reward units, to produce baseline RTs of the order 200 ms—times which are typical for the saccadic system. Trials on which the RT was greater than 1 second were considered as errors (i.e. zero reward). Finally, the mean optimum RT and accuracy was calculated for each condition. Results are shown in **Fig. 4.**

These simulations showed that reward increased accuracy (**Fig. 4B** and **4D**). When signal-to-noise μ/σ was *high*, reward also shortened the optimal RT. In contrast, with very low signal-to-noise ratios (**Fig. 4E,** blue lines), increasing reward actually prolonged RT, leading to a classical speed accuracy trade-off. This reflected a greater investment in precision with reward, despite a lower threshold, in situations where a task was difficult. This task-dependence might explain why reward's effects on speed and accuracy vary from study to study [S26, 27].

Some drift diffusion models have also included stochastic starting points of the accumulator, such that $A(0)$ is chosen from a uniform distribution in a range ±α [S28–30]. This allows higher error rates for early responses, as seen in our data. Qualitatively similar results were obtained using a starting-point variability of α=0.1 and 0.6, rather than zero.

We examined the predicted conditional accuracy function and delta plot (effect of reward on shortening RT as a function of RT bin). For this distributional analysis, starting point variability was fixed at ±0.3, and temporal discount rate was 5 s^{-1}, to generate fast errors. Predictions were generated for three levels of reward, and 500 responses were simulated 20 times. The effect of reward was plotted using the same techniques as for the experimental data, using a moving window over RT quantiles to calculate reward effect on accuracy and RT (**Fig. S1B** and **D**). These plots illustrate the qualitative match between the empirical effects of reward, and those predicted by the model.

The methods above allow motivational effects to be predicted. Such effects have previously been summarised in terms of attention, alertness or arousal, but have not been quantified before in terms

of cost-benefit analysis. In its basic form, the ability to attenuate noise has the effect of increasing the signal-to-noise ratio when weighing up uncertain evidence. The cost could equally be applied to race models of decisions[S31], for example by amplifying the difference in build-up rate of competing processes.

Empirical quantification of reward's effect on speed and accuracy

1. Task Instructions

Forty healthy volunteers participated, mean age 46 (Supplemental Data **Table S2**). Participants were informed that they had to keep their eyes on the illuminated disc, while they listened to how much money was available on the upcoming trial. When the display changed, the remaining two discs would light up, one slightly later than the other. The aim was to look towards the disc that illuminated second. They were told that they had to move as quickly as possible, and that the time they took to reach the target would be used to calculate the *proportion of the stake* they actually won. The total winnings would be added up and paid in cash after the experiment.

2. Task

The task is a variant of the double step paradigm [S32, 33] and aimed to maximise oculomotor capture by the salient distractor [S34]. Three screen locations were indicated by dim grey discs, each 4° diameter, arranged in an equilateral triangle 11.4° apart (**Fig. 5A**). One disc was illuminated in yellow colour at the start of the trial, and participants were required to fixate this for 500 ms to start the trial. Participants heard a recording of a voice speaking "0p maximum", "10p maximum" or "50p maximum" lasting 1200 ms, followed by a variable foreperiod of 400-600 ms. Then the target was dimmed and simultaneously one of the two remaining discs brightened (the distractor). After a delay, the third disc brightened also (the target). The delay was one of 40 ms, 80 ms or 120 ms for the younger participants (n=18), and was fixed at 80 ms for the older control participants (n=22) and PD patients. The target display remained visible until a saccade terminated at the target disc.

The time taken to reach the target (from distractor onset until gaze arrived at the target) was used to calculate reward (**Fig. 5B**) as follows:

$$R\left(t\right) = R_{\max} \cdot \min\left(1 - e^{\frac{t-\tau_2}{\tau_1}}\right) \tag{15}$$

where R is reward for the current trial, t is the time taken to reach the target, $R_{\max}$ is the maximum reward that could be won on a given trial, and τ_1 and τ_2 are adaptive reward criteria (see below).

Reward was displayed as a red integer in the target disc as soon as the target was reached, for 800 ms. This was accompanied by a bell sound when the reward was 10p or greater, or a 'cash register' sound when 30p or greater was won. The target location was then used as the starting point for the next trial.

Unknown to participants, the RT criteria τ_1 and τ_2 were adaptively adjusted using the last 20 trials. The criteria tracked quantiles of the RT distribution, keeping 10% of trials faster than τ_1 and 30% of trials slower than τ_2. This ensured that participants experienced the full range of outcomes irrespective of their baseline reaction speed.

Participants performed 4 blocks of 54 trials each, with a 2 minute break between blocks, with drift-correction before each block. There were three reward cues of 0p, 10p, 50p, three possible starting

locations, and two possible target locations relative to this starting location. Trials were intermixed and balanced between blocks.

3. Materials

Stimuli were presented on a CRT resolution 1280 x 1024 pixels at 100 Hz, at a distance of 60 cm from the eye, controlled by MATLAB and PsychToolbox. Eye movements and pupil size were recorded by a desktop-mounted Eyelink 1000 Hz infra-red eye tracker with head rest, with online parsing of saccade endpoints for reward determination. Velocity and endpoints were calculated offline (described below). Auditory reward cues were presented through a loudspeaker beneath the desk.

4. Saccade analysis

Saccades were parsed using criteria on velocity of $30°s^{-1}$, acceleration > $8000°s^{-2}$ and amplitude > 1°. Saccadic RTs were calculated as the time from distractor onset until this threshold was exceeded. Responses were classified according to the endpoint of the first saccade (**Fig. 5C**).

The trial was classified according to the first saccade made after the onset of the distractor. Correct trials were those in which the saccade's amplitude was greater than 5°, and its endpoint was closer to the target than the distractor. The trial was classed as an oculomotor capture error if the first saccade's amplitude was greater than 5°, and its endpoint was closer to the distractor than to the target. Other trials (average 4.8%) were discarded. The lenient accuracy criteria, and large diameter of target discs, aimed to minimise the need for endpoint precision in this task.

For correct trials, the peak velocity of the first saccade was calculated using 4 ms windows from saccade onset to termination, discarding any speeds greater than $3000°s^{-1}$, and any saccades during which tracking was lost. Saccade amplitude was defined as the distance from fixation to the first saccade's endpoint. Amplitude variability was calculated as the standard deviation of saccade amplitudes for each reward condition for each subject.

5. PD Patients

Nineteen patients with mild or moderate PD who fulfilled the criteria for the Queen Square Brain Bank for PD (Gibb and Lees 1988) were recruited from the neurology clinic at the National Hospital for Neurology and Neurosurgery. The mean UPDRS was 23.1 (s.d. 10.1). All patients were on medication; 15 were taking levodopa, and 10 were taking a dopamine agonist. The mean levodopa equivalent dose was 507 mg (**Table S2**).

The mean age of the patients was 65.3 yrs (s.d. 9.0) compared to 62.5 (s.d. 8.9) for the older control participants. Cognitive impairment was screened for using either Montreal cognitive assessment (MoCA) ≥ 26 or mini-mental state examination (MMSE) score ≥ 26; two patients had mild cognitive impairment with MoCA of 25. Two patients did not have cognitive tests but were still in full-time work. Depression was excluded using the Hospital Anxiety and Depression scale [S35]. All patients had normal or corrected-to-normal colour vision. Symptomatically, 2 patients had significant functional impairment as determined by Schwab and England ADL score (one patient 50%, one patient 60%, all other patients 80% or above).

Sixteen patients were tested at 9 am, and three at 2 pm. Eight patients had previously performed the task 2 weeks earlier. Before undertaking the rewarded oculomotor capture task, patients also completed 96 trials of a simple prosaccade task and 96 trials of a simple antisaccade task, as a baseline measure (not reported here). Then the oculomotor task was performed as described above. One patient completed only 192 trials out of 216, and the analysis was conducted on the reduced

number of trials.

6. Statistics

The effect of reward in healthy participants was assessed by calculating the mean RT, mean peak saccade velocity, proportion of oculomotor capture (errors), and variability in saccade amplitude for each condition (**Fig. 6**). The proportion of capture was arcsine transformed. A repeated measures general linear model yielded the effect of reward on each of the four measures. To compare patients with PD with controls (**Fig.7**), a mixed-effects model was used with Group as a between-subjects factor. This yielded main effects of reward and group, plus an interaction term for group x reward.

The cost-of-control model predicted that with certain combinations of noise and temporal discount (low σ and high k), motor variability might *increase* with reward rather than decrease (**Fig. 3C**, upper graphs). Since the group-level effects of reward and PD on variability were not significant (trend to interaction p=0.077), we examined whether individuals had effects of reward in either direction. A significant effect of reward (for one participant) was defined as the variability difference between the lowest reward and the highest reward level lying outside the confidence intervals for this difference when the trials were permuted over the reward levels (5000 permutations, threshold p=0.05). Of the 39 control participants, 12 had significant effects of reward: 10 negative (reduction of variability with reward) and 2 positive in direction, after correction for 39 multiple comparisons. Of the 19 PD patients, 2 had significant effects, one positive and one negative. A scatter plot of the effects (**Fig. S4A**) shows the significant individuals with crosses. The reward effects on variability were found to correlate strongly with effects on velocity in the control group (r^2=0.232, p<0.001), with a similar trend in the PD group (r^2=0.20, p=0.085).

7. Velocity effects were not attributable to amplitude or curvature

Saccade velocity is known to be relatively rigidly determined by saccade amplitude [S36, 37], according to a law known as the “main sequence”[S38]. Can the invigorating effect of reward be attributed simply to increased saccade amplitude when higher incentives were available? Saccade amplitudes did increase with larger rewards, although the absolute effect was small: mean hypometria of 0.04° with high reward compared to 0.26° for low reward (F(2,76)=18.4, p<0.001). Using stepwise regression to subtract out the effect of amplitude, velocity remained significantly increased by incentives over and above what was predictable by amplitude changes (F(2,76)=9.38, p=0.002). This indicates that rewards invigorate movement speed *independently* of movement size, transgressing the “main sequence” relationship between velocity and amplitude[S38].

Saccades were sometimes curved, either towards or away from the distractor (**Fig. 5C**). Could the slower velocities observed with low incentives simply be due to increased trajectory curvature? To factor out curvature, the maximal deviation of the saccade trajectory away from a straight line was used as a covariate in stepwise regression against saccade velocity, to obtain residuals that did not depend on curvature. There was no effect of curvature on velocity (t=0.45; p>0.05), and reward significantly increased velocity even when curvature was regressed out (t(38)=6.64, p<0.001).

8. Conditional accuracy functions and Delta plots

Reward may have differential effects on slower and faster responses. The distribution of RTs was analysed by novel sliding-window analyses (**Figs. 6F** and **S1**), which improve upon previous binned (Vincentised) methods [S39, 40]. Although quintile binning improves significantly on a simple mean or median [S41], firstly it assumes that 5 bins is the appropriate number, and secondly, the bin edges are chosen are essentially arbitrary [S42]. Furthermore, parametric statistical approaches rely heavily on selecting appropriate hypotheses about the different RT bins. To overcome these limitations, we devised continuous sliding window versions of the conditional accuracy function,

examining accuracy as a function of RT, and a continuous "delta plot", which examines the effect of reward on RT as a function of RT.

Firstly, we asked how reward influenced *accuracy* at different reaction times. To do this, we constructed a conditional accuracy function [S43] (**Figs. S1A** and **S1B**), in which a sliding bin of width 20% quantile was moved smoothly, in one-percentile steps, along the RT distribution. The proportion of correct saccades was calculated in each window. To test for significant effects of reward, correcting for multiple comparisons over multiple windows, a permutation test was performed by randomly re-ordering the reward conditions within each subject's data, and computing across the whole time series the maximum value of the t-statistic [S44]. The resulting null distribution of maximum *t* over all the permutations can be thresholded at a given alpha-level to control the family-wise error rate (the probability that at any of the many t-tests across the timepoints will be significant, over all permutations). Comparing the t-statistic of the actual data to the null distribution yielded a corrected p-value for time window.
Early responses were more likely to be oculomotor capture errors, as shown in the conditional accuracy plot (i.e. accuracy in each RT time bin, **Fig. 6F**). For a given reward level, a speed-accuracy trade-off holds. When reward is increased, the conditional accuracy function undergoes a parallel shift, contravening this trade-off. Healthy controls showed clear effects of reward ($p<0.05$ for RT bins < 284 ms), but crucially, the reward effect was absent in PD patients ($p>0.05$, **Fig. S1A**). Individuals with PD were generally slower than controls, and more accurate at later time-bins, yet *less* accurate at early time bins.

Secondly, we examined the effect of reward on RT at different time-points during the RT distribution (**Fig. S1C**). A similar analysis was conducted to obtain "delta plots" which compared the RT distributions of different reward levels[S45]. A 20% quantile bin was moved smoothly, in one-percentile steps, over the two RT distributions, and the means are subtracted, to give a point-wise estimate of the effect of reward. The analysis was performed for both 10p–0p and 50p–10p, and the average of these two was plotted. Significance of reward effects was calculated using a permutation test. The effect of reward on accuracy at a given bin was compared to the null distribution (i.e. reward levels permuted) of the maximum *t* statistic over all bins.

Reward significantly shortened RT in controls (blue) through most of the RT distribution ($p<0.05$), unlike in PD. Later responses showed the greatest speeding by reward. This effect of RT was also predicted by the simulation (**Fig. S1D**). Thus the moment-to-moment effects of incentive are consistent with the control cost model.

9. Fatigue could not explain reduced reward sensitivity in PD

Fatigue is known to be more prevalent in PD, and might impact reward sensitivity. To exclude the possibility that motivational effects were related to fatigue, trends over time-on-task for velocity, RT, variability and accuracy were measured. There were no significant effects on velocity, endpoint variability, or oculomotor capture. RT did decrease with time on task ($F(1,37)=10.4$, $p=0.003$), so we compared the effects of reward on early (first half) and late (second half) trials, and found no interaction with reward, and no interaction with group ($F(1,120)=2.3$, $p=0.13$). Thus none of the reported effects were attributable to time-on-task.

10. Analysis of distractor-target delay and error trials

In order to look for evidence that race processes generated the observed behaviour, the probability of oculomotor capture errors was broken down by the distractor-target delay in the young healthy control group (n=17). Longer delays led to more errors (**Fig. S2A**, repeated-measures 1-way ANOVA $F(2,32)=3.77$; $p=0.034$).

The reaction time on error trials was measured as the time from the distractor onset until the first saccade greater than 5 degrees that ended closer to the distractor than the target. The error RTs were consistently shorter than correct RTs (**Figure S2B**), consistent with a race model interpretation, or a drift-diffusion model with variable initial state. The time to correct each error was determined as the time from the error RT until the eyes landed on the correct target. Across subjects, these times correlated strongly with the error RT (**Figure S2C**, r^2=0.52, p<0.001). These times were significantly longer than both correct and error RTs (mean 305 ms, s.e.m. 18). The mean cumulative distributions of error RT, correct RT and correction RT were plotted on reciprocal-x probit-y axes, on which a normally distributed rate (1/RT) would appear as a straight line [S31]. Quantiles of the cumulative RT distribution were averaged across participants (**Fig. S3A**), showing that correct responses were later than errors, with a narrower variability, and stronger effects of delay.

To examine whether, on a trial-by-trial basis, the time of an error determined the time of its corresponding correction, the mean time to correct an error was plotted as a function of the error RT bin (**Fig. S3B**). A sliding window of width 20 percentiles was moved along the error RTs in 1 percentile intervals. Fast errors took relatively longer to correct than later errors, consistent with previous reports[S32, 46]. However for the slowest error responses the reverse effect was seen. We suggest that those trials may represent "lapses", on which both the error and the correction were slow. The correction time was much shorter when the distractor-target delay was longer ($F(2,32)=20$, p<0.001), suggesting parallel preparation of movements to the distractor and target (**Fig. S3C**). Reward markedly reduced the RT of error-corrections (**Fig. S3D**).

Model fit of reward's effects on motor control

We fitted the cost of control model to velocity and endpoint error data from each participant. First, the optimal command [u_F,u_P] was found (**Fig. 3B, C**) for each point in a 50 x 50 x 50 volume (logarithmic ranges, reward = 0.1 to 10, σ = 0.1 to 100 and k = 0.01 to 10). This predicts, for each combination of subject-level parameters σ, k, and σ_0, how a subject's variability and velocity would vary with reward. Each participant's actual mean peak velocity and saccade amplitude variability were measured at each reward level. These data were then fitted to the parameters k, σ and σ_0, by minimising the squared error of the model predictions to the data (across velocity and variability for each of the 3 reward levels). Thus 3 parameters were obtained for each participant. Since the units in the model were arbitrary, four more constants were fitted *across all* participants. These were: the unit of variability= 0.61°, unit of velocity =103°/s, reward unit = 7 pence, minimum reward level = 19% of maximum reward.

1 .Model comparison

We compared our precision-and-force model ("Model 1") with two simpler models. Model 2 allowed each participant to 'choose' only the force u_F for each reward level, with the precision u_P fixed across reward levels. This model therefore only allows for a standard speed-accuracy trade-off, and is equivalent to the "orthodox" model. Model 3 held the force constant, but allowed individuals to 'choose' the precision for each reward level. This model thus includes *only* effects that would be traditionally regarded as "attentional" or motivational. All models had three parameters that characterised each subject: k, σ and σ_0.

To determine if both of these dimensions of variability are required to explain the data, the Akaike information (AIC) was calculated for each of the 3 models, across all participants, using least squares log likelihood[S47]. AIC measures a model's fit, accounting for its complexity. The model with the lowest AIC is strongly supported if the AIC difference is larger than 4 (corresponding to a probability ratio of 0.02). According to AIC, our new force-and-precision model fits the data best (**Table S3**).

2. Patient vs control comparison

Which model parameter best accounted for the difference between patients with PD and controls? To answer this, the best fitting parameters (σ, σ_0, k) for each participant were found, using the best model, i.e. the model that allowed both u_F and u_P to vary. Across participants, both σ and σ_0 followed a Gaussian distribution (according to the Kolmogorov-Smirnoff test), but k was skewed, and was therefore log-transformed, restoring normality, before comparison. PD patients were compared with an age-matched subset of healthy controls, n=22. Patients had significantly increased noise control costs σ (two-tailed unpaired t-test, $t(36)=2.21$, $p=0.034$, **Table S1**). Neither their temporal discount rate nor their baseline variability were significantly different from healthy people ($p>0.05$). One interpretation of these data is that PD patients go slower in order to reduce their motor variability in the face of an increased cost for controlling internal noise.

Our results are suggestive, but not conclusive, that dopamine depletion may lead to a higher cost of control. Although our patients had mild-to-moderate PD without dementia, we cannot rule out pathology in non-dopaminergic systems, e.g. cholinergic and serotonergic dysfunction[S48]. On physiological grounds, however, it has been suggested that dopamine itself may have direct effects of increasing synaptic gain[S49], or suppressing membrane noise in sensory or motor neurones to improve signal-to-noise ratios[S50]. Thus it could be suggested that dopamine, by enhancing signal-to-noise, may reduce the cost of control. Future work could unite the role of dopamine in reward and effort by considering how it generates performance improvements at the neural level.

Supplementary References

S1. Tomlinson, C. L., Stowe, R., Patel, S., Rick, C., Gray, R., and Clarke, C. E. (2010). Systematic review of levodopa dose equivalency reporting in Parkinson's disease. Mov. Disord. *25*, 2649–2653.

S2. Rigoux, L., and Guigon, E. (2012). A Model of Reward- and Effort-Based Optimal Decision Making and Motor Control. PLoS Comput Biol *8*, e1002716.

S3. Shadmehr, R., Xivry, J. J. O. de, Xu-Wilson, M., and Shih, T.-Y. (2010). Temporal Discounting of Reward and the Cost of Time in Motor Control. J. Neurosci. *30*, 10507–10516.

S4. Shadmehr, R., and Krakauer, J. W. (2008). A computational neuroanatomy for motor control. Exp. Brain Res. *185*, 359–381.

S5. Davis, M. H. A., and Vinter, R. B. (1985). Stochastic Modelling and Control (Dordrecht: Springer Netherlands) Available at: http://cds.cern.ch/record/1619764 [Accessed August 11, 2014].

S6. Todorov, E. (2005). Stochastic Optimal Control and Estimation Methods Adapted to the Noise Characteristics of the Sensorimotor System. Neural Comput. *17*, 1084–1108.

S7. Qian, N., Jiang, Y., Jiang, Z.-P., and Mazzoni, P. (2012). Movement Duration, Fitts's Law, and an Infinite-Horizon Optimal Feedback Control Model for Biological Motor Systems. Neural Comput. *25*, 697–724.

S8. Chen-Harris, H., Joiner, W. M., Ethier, V., Zee, D. S., and Shadmehr, R. (2008). Adaptive Control of Saccades via Internal Feedback. J. Neurosci. *28*, 2804–2813.

S9. Joiner, W. M., FitzGibbon, E. J., and Wurtz, R. H. (2010). Amplitudes and directions of individual saccades can be adjusted by corollary discharge. J. Vis. *10*, 22.

S10. Haruno, M., Wolpert, D., and Kawato, M. (2001). MOSAIC Model for Sensorimotor Learning and Control. Neural Comput. *13*, 2201–2220.

S11. Todorov, E. (2004). Optimality principles in sensorimotor control. Nat. Neurosci. *7*, 907–915.

S12. Adams, R. A., Shipp, S., and Friston, K. J. (2013). Predictions not commands: active inference in the motor system. Brain Struct. Funct. *218*, 611–643.

S13. Krakauer, J. W., and Mazzoni, P. (2011). Human sensorimotor learning: adaptation, skill, and beyond. Curr. Opin. Neurobiol. *21*, 636–644.

S14. Jiang, J., Heller, K., and Egner, T. (2014). Bayesian modeling of flexible cognitive control. Neurosci. Biobehav. Rev. Available at: http://www.sciencedirect.com/science/article/pii/S0149763414001390.

S15. Seung, H. S., and Sompolinsky, H. (1993). Simple models for reading neuronal population codes. Proc. Natl. Acad. Sci. *90*, 10749–10753.

S16. Johnston, W. A., and Strayer, D. L. (2001). 15 A dynamic, evolutionary perspective on attention capture. In Advances in Psychology, Charles L. Folk and Bradley S. Gibson, ed. (North-Holland), pp. 375–397. Available at: http://www.sciencedirect.com/science/article/pii/S0166411501800170.

S17. Haith, A. M., Reppert, T. R., and Shadmehr, R. (2012). Evidence for Hyperbolic Temporal Discounting of Reward in Control of Movements. J. Neurosci. *32*, 11727–11736.

S18. Trommershäuser, J., Maloney, L. T., and Landy, M. S. (2003). Statistical decision theory and trade-offs in the control of motor response. Spat. Vis. *16*, 255–275.

S19. Trommershäuser, J., Gepshtein, S., Maloney, L. T., Landy, M. S., and Banks, M. S. (2005). Optimal Compensation for Changes in Task-Relevant Movement Variability. J. Neurosci. *25*, 7169–7178.

S20. Schütz, A. C., Trommershäuser, J., and Gegenfurtner, K. R. (2012). Dynamic integration of information about salience and value for saccadic eye movements. Proc. Natl. Acad. Sci. *109*, 7547–7552.

S21. Harris, C. M., and Wolpert, D. M. (1998). Signal-dependent noise determines motor planning.

Nature *394*, 780–784.
S22. Niv, Y., Daw, N. D., Joel, D., and Dayan, P. (2007). Tonic dopamine: opportunity costs and the control of response vigor. Psychopharmacology (Berl.) *191*, 507–520.
S23. Bijleveld, E., Custers, R., and Aarts, H. (2009). The Unconscious Eye Opener Pupil Dilation Reveals Strategic Recruitment of Resources Upon Presentation of Subliminal Reward Cues. Psychol. Sci. *20*, 1313–1315.
S24. Ratcliff, R. (1979). Group reaction time distributions and an analysis of distribution statistics. Psychol. Bull. *86*, 446–461.
S25. Ratcliff, R., and Frank, M. J. (2012). Reinforcement-Based Decision Making in Corticostriatal Circuits: Mutual Constraints by Neurocomputational and Diffusion Models. Neural Comput. *24*, 1186–1229.
S26. Rorie, A. E., Gao, J., McClelland, J. L., and Newsome, W. T. (2010). Integration of Sensory and Reward Information during Perceptual Decision-Making in Lateral Intraparietal Cortex (LIP) of the Macaque Monkey. PLoS ONE *5*, e9308.
S27. Wang, L., Yu, H., and Zhou, X. (2013). Interaction between value and perceptual salience in value-driven attentional capture. J. Vis. *13*. Available at: http://www.journalofvision.org/content/13/3/5.
S28. Ratcliff, R. (1981). A theory of order relations in perceptual matching. Psychol. Rev. *88*, 552–572.
S29. Ratcliff, R., and Rouder, J. N. (1998). Modeling Response Times for Two-Choice Decisions. Psychol. Sci. *9*, 347–356.
S30. Ratcliff, R., and McKoon, G. (2007). The Diffusion Decision Model: Theory and Data for Two-Choice Decision Tasks. Neural Comput. *20*, 873–922.
S31. Carpenter, R. H. S., and Williams, M. L. L. (1995). Neural computation of log likelihood in control of saccadic eye movements. Nature *377*, 59–62.
S32. Camalier, C. R., Gotler, A., Murthy, A., Thompson, K. G., Logan, G. D., Palmeri, T. J., and Schall, J. D. (2007). Dynamics of saccade target selection: Race model analysis of double step and search step saccade production in human and macaque. Vision Res. *47*, 2187–2211.
S33. Ramakrishnan, A., Sureshbabu, R., and Murthy, A. (2012). Understanding How the Brain Changes Its Mind: Microstimulation in the Macaque Frontal Eye Field Reveals How Saccade Plans Are Changed. J. Neurosci. *32*, 4457–4472.
S34. Theeuwes, J., Kramer, A. F., Hahn, S., and Irwin, D. E. (1998). Our Eyes do Not Always Go Where we Want Them to Go: Capture of the Eyes by New Objects. Psychol. Sci. *9*, 379–385.
S35. Zigmond, A. S., and Snaith, R. P. (1983). The Hospital Anxiety and Depression Scale. Acta Psychiatr. Scand. *67*, 361–370.
S36. Bahill, A. T., Clark, M. R., and Stark, L. (1975). The main sequence, a tool for studying human eye movements. Math. Biosci. *24*, 191–204.
S37. Harris, C. M., and Wolpert, D. M. (2006). The Main Sequence of Saccades Optimizes Speed-accuracy Trade-off. Biol. Cybern. *95*, 21–29.
S38. Chen, L. L., Hung, L. Y., Quinet, J., and Kosek, K. (2013). Cognitive regulation of saccadic velocity by reward prospect. Eur. J. Neurosci. *38*, 2434–2444.
S39. Vincent, S. B. (1912). The functions of the vibrissae in the behavior of the white rat ... (University of Chicago).
S40. Ratcliff, R. (1979). Group reaction time distributions and an analysis of distribution statistics. Psychol. Bull. *86*, 446–461.
S41. Dawson, M. R. W. (1988). Fitting the ex-Gaussian equation to reaction time distributions. Behav. Res. Methods Instrum. Comput. *20*, 54–57.
S42. Rouder, J. N., and Speckman, P. L. (2004). An evaluation of the Vincentizing method of forming group-level response time distributions. Psychon. Bull. Rev. *11*, 419–427.
S43. Wood, C. C., and Jennings, J. R. (1976). Speed-accuracy tradeoff functions in choice reaction time: Experimental designs and computational procedures. Percept. Psychophys. *19*, 92–102.

S44. Nichols, T. E., and Holmes, A. P. (2002). Nonparametric permutation tests for functional neuroimaging: A primer with examples. Hum. Brain Mapp. *15*, 1–25.
S45. Ridderinkhof, K. R., W.P.M. van den Wildenberg, J. Wijnen, and B. Burle (2004). Response Inhibition in Conflict Tasks Is Revealed in Delta Plots. In Cognitive neuroscience of attention (Guilford Press), pp. 369–377. Available at: http://dare.uva.nl/record/158289 [Accessed April 2, 2013].
S46. Becker, W., and Jürgens, R. (1979). An analysis of the saccadic system by means of double step stimuli. Vision Res. *19*, 967–983.
S47. Akaike, H. (1980). Likelihood and the Bayes procedure. Trab. Estad. Investig. Oper. *31*, 143–166.
S48. Francis, P. T., and Perry, E. K. (2007). Cholinergic and other neurotransmitter mechanisms in Parkinson's disease, Parkinson's disease dementia, and dementia with Lewy bodies. Mov. Disord. *22*, S351–S357.
S49. Ashby, F. G., and Casale, M. B. (2003). A model of dopamine modulated cortical activation. Neural Netw. *16*, 973–984.
S50. Kroener, S., Chandler, L. J., Phillips, P. E. M., and Seamans, J. K. (2009). Dopamine Modulates Persistent Synaptic Activity and Enhances the Signal-to-Noise Ratio in the Prefrontal Cortex. PLoS ONE *4*, e6507.
