# Supplementary material for: Reward Pays the Cost of Noise Reduction in Motor and Cognitive Control
Source: Curr Biol. 2015 Jun 29;25(13):1707–16. doi: 10.1016/j.cub.2015.05.038 (PMC4557747; doi:10.1016/j.cub.2015.05.038)
Supplement: Document S2. Article plus Supplemental Information [file mmc2.pdf]

Article

Current Biology

Reward Pays the Cost of Noise Reduction in Motor and Cognitive Control

Graphical Abstract

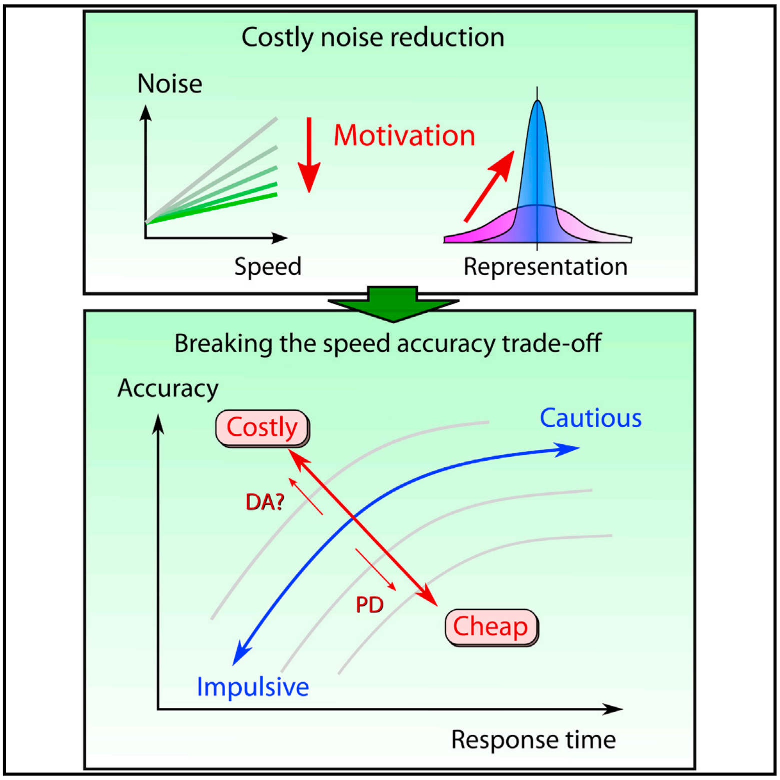

Authors

Sanjay G. Manohar, Trevor T.-J. Chong, Matthew A.J. Apps, ..., Paul R. Jarman, Kailash P. Bhatia, Masud Husain

Correspondence

sanjay.manohar@ndcn.ox.ac.uk

In Brief

Manohar et al. investigate how motivation by reward can improve both speed and accuracy, apparently exceeding the limits of the speed-accuracy trade-off. They propose a cost for reducing intrinsic neural noise. Optimizing this cost predicts both motor and cognitive performance. The cost of control may be increased in Parkinson's disease.

Highlights

- The speed-accuracy trade-off in motor and cognitive control can be broken by reward
- Apparent limits of performance can be overcome by motivation
- A cost for reducing intrinsic neural noise quantitatively explains such improvements
- Reduced reward effects in Parkinson's disease suggest an increased cost of control

Manohar et al., 2015, Current Biology *25*, 1707–1716
June 29, 2015 ©2015 The Authors
http://dx.doi.org/10.1016/j.cub.2015.05.038

CellPress

Current Biology
Article

CellPress

Reward Pays the Cost of Noise Reduction in Motor and Cognitive Control

Sanjay G. Manohar,[1,2,3,4,5,*] Trevor T.-J. Chong,[1,2] Matthew A.J. Apps,[1,2] Amit Batla,[3,5] Maria Stamelou,[3,5] Paul R. Jarman,[5] Kailash P. Bhatia,[3,5] and Masud Husain[1,2,3,4,5]
[1]Nuffield Department of Clinical Neurosciences, John Radcliffe Hospital, Oxford OX3 9DU, UK
[2]Department of Experimental Psychology, University of Oxford, Oxford OX1 3UD, UK
[3]Institute of Neurology, University College London, London WC1N 3BG, UK
[4]Institute of Cognitive Neuroscience, University College London, London WC1N 3AR, UK
[5]National Hospital for Neurology and Neurosurgery, Queen Square, London WC1N 3BG, UK
*Correspondence: sanjay.manohar@ndcn.ox.ac.uk
http://dx.doi.org/10.1016/j.cub.2015.05.038
This is an open access article under the CC BY license (http://creativecommons.org/licenses/by/4.0/).

SUMMARY

Speed-accuracy trade-off is an intensively studied law governing almost all behavioral tasks across species. Here we show that motivation by reward breaks this law, by simultaneously invigorating movement and improving response precision. We devised a model to explain this paradoxical effect of reward by considering a new factor: the *cost of control*. Exerting control to improve response precision might itself come at a cost—a cost to attenuate a proportion of intrinsic neural noise. Applying a noise-reduction cost to optimal motor control predicted that reward can increase both velocity and accuracy. Similarly, application to decision-making predicted that reward reduces reaction times and errors in cognitive control. We used a novel saccadic distraction task to quantify the speed and accuracy of both movements and decisions under varying reward. Both faster speeds and smaller errors were observed with higher incentives, with the results best fitted by a model including a precision cost. Recent theories consider dopamine to be a key neuromodulator in mediating motivational effects of reward. We therefore examined how Parkinson's disease (PD), a condition associated with dopamine depletion, alters the effects of reward. Individuals with PD showed reduced reward sensitivity in their speed and accuracy, consistent in our model with higher noise-control costs. Including a cost of control over noise explains how reward may allow apparent performance limits to be surpassed. On this view, the pattern of reduced reward sensitivity in PD patients can specifically be accounted for by a higher cost for controlling noise.

INTRODUCTION

A fundamental and long-established finding in human and animal behavior is the phenomenon of speed-accuracy trade-off: when actions are performed faster, they are less accurate [1]. This principle applies widely across both motor and cognitive performance [2, 3]. Current theoretical approaches suggest that reward may increase the speed of actions, but at the cost of their accuracy. Recently however, some studies have reported that reward *simultaneously* increases both velocity and precision of motor control [4] and can reduce reaction times and error rates in decisions involving cognitive control [5, 6]. Here we provide a unified quantitative framework for how and why motivation by reward in fact contravenes the speed-accuracy trade-off, simultaneously improving both speed and accuracy in these diverse domains. According to our model, the speed-accuracy trade-off is not a hard barrier but rather a gray zone where the apparent limit of performance can be determined by reward (Figure 1). We apply the theory to both movements and decisions. We test our framework in healthy participants and also compare patients with Parkinson's disease to a control group to demonstrate the role of reward and dopamine in accounting for the cost of control.

According to motor control theory, the speed-accuracy trade-off arises because larger or faster movements are subject to greater motor noise [7, 8]. Similarly, in the domain of cognitive control, models of decision-making predict a speed-accuracy trade-off, on the assumption that faster responding implies less time to weigh up evidence and thus more error-prone choices [9]. Since organisms prefer to obtain reward sooner [10–13], high reward results in greater speed, or vigor, as measured by either movement time or reaction time [14–16]. Crucially, however, if noise ultimately limits performance, then the effect of reward on invigorating actions should lead to fast but inaccurate responding—in conflict with observed behavior [4, 5].

Current accounts, therefore, do not explain why we can perform well (i.e., be both fast and accurate) when motivated by reward but at other times are seemingly suboptimal [5, 17]. We propose a quantitative account of the effects of reward in terms of paying the *cost of control.* Such a factor has been invoked recently to explain qualitatively how incentive might increase "cognitive control" by overcoming a cost [18–20]. Here, we consider a hidden *precision cost,* analogous to the cost of motor commands in optimal control theory.

Numerically, we propose that the brain might put a fixed price on attenuating noise by a certain proportion. Noise-reduction

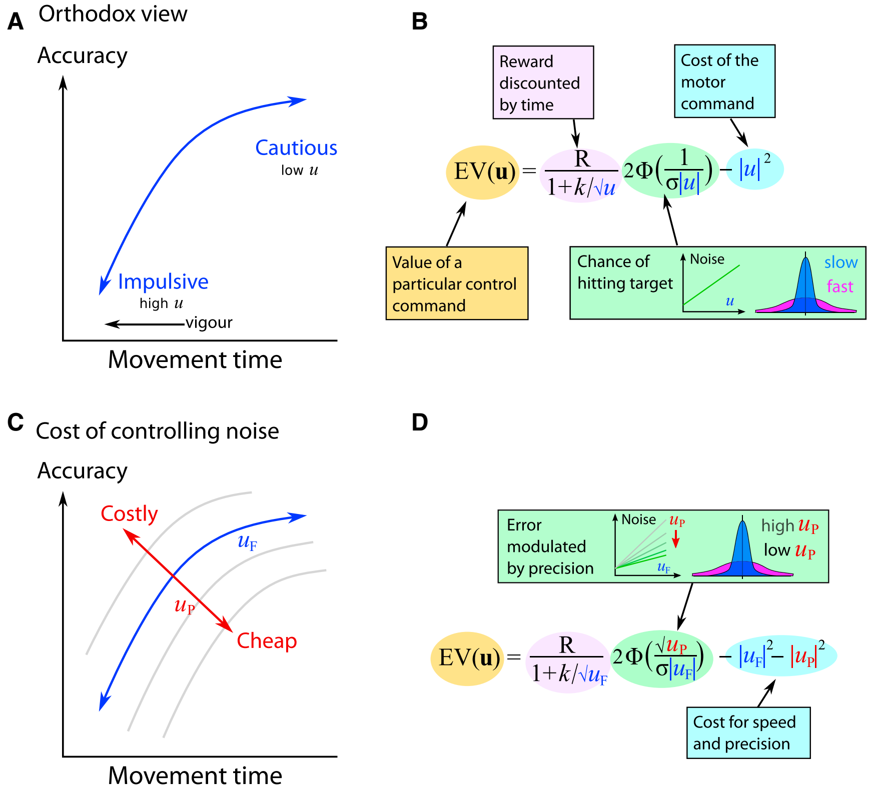

Figure 1. Breaking the Speed-Accuracy Trade-Off

A conventional account of the speed-accuracy trade-off is shown on top (A and B). Lower panels illustrate the inclusion of a precision cost (C and D).

(A) As movement time decreases (and speed increases), accuracy declines. This is because faster movements require larger forces, which are susceptible to proportionally higher noise. Because noise is taken to be rigidly proportional to motor command size, behavior is constrained such that accuracy depends on speed. This is captured by a speed-accuracy trade-off contour (illustrated in blue in the schematic).

(B) Standard motor control models determine the optimal movement as the one that gives the highest average payoff. The expected value (EV) of a movement depends on the motor command **u**. EV can be expressed in terms of the reward, discounted by the movement time (pink). Larger motor commands give faster movements, leading to earlier reward, which is more valuable (here we use hyperbolic temporal discounting). The reward is further reduced by movement error, which reduces the probability of success (green). Under the orthodox view, this error is determined by noise proportional to the motor command. This means that faster movements will have less accurate endpoints and thus have a lower chance of winning a reward. The final term is the energetic cost of the motor command itself (blue). The balance between time and accuracy is governed only by the movement speed, which guarantees that speed and accuracy trade off with one another.

(C) We suggest that reward has a motivating effect that permits both faster and more precise behavior. Incentivization by reward thus produces a change in a direction perpendicular to the blue line (red arrows), contrary to the speed-accuracy trade-off. By allowing both force and movement precision to be varied, our model allows two degrees of freedom over speed-accuracy space.

(D) In order to explain violations of the speed-accuracy constraint, we introduced an additional precision command. The precision command reduces noise (u_P, shown in red), complementing the usual force command (u_F, blue). We propose that this command is itself costly, in the same way as the force command u_F, leading to a cost term $|u_P|^2 + |u_F|^2$. Optimizing EV by selecting both the precision and force would allow accuracy to improve independently of speed, but constrained by this cost. Higher incentives allow a greater investment in precision, rather than a trade-off with speed, so genuine performance improvements are possible.

mechanisms might include corrective feedback signals [21], allocating more resources to representing that signal [22], or attenuating currently irrelevant information [23]. Each of these mechanisms may incur a cost to the organism in terms of opportunity cost, neuronal resources, and/or energetic cost. However, regardless of the ultimate nature of the cost, optimizing the level of precision provides a unified mathematical way of describing the deployment of resources such as effort, attention, and executive control [24].

Our framework makes several key predictions. First, when applied to optimal motor control, the precision cost leads to the prediction that when incentives are high, movements can become both fast *and* precise. Second, when applied to decision processes, in the form of a rise-to-threshold model of reaction time (RT), a noise-reduction cost can also quantitatively explain motivational effects on RTs and error rates. From a cognitive neuroscience perspective, this as approach allows quantification of "effort costs" of deploying increased attention—effectively amplifying relevant sensory signals or suppressing irrelevant ones [25]—when the reward are high.

To test our theory and quantify how reward can make us apparently "more optimal," we devised a novel saccadic task in which participants have to look toward a target quickly, while avoiding a salient early-onset distractor (Figure 5). The velocity of a saccade has often been regarded as rigidly determined by its amplitude [26–28], until recent studies demonstrated modulation by reward [4, 14, 16]. In our experiment, by manipulating incentives on each trial, we measured exactly how reward increases saccade velocity (speed) and endpoint accuracy. Furthermore, we were able to separately index cognitive control by measuring errors to the distractor and their relation to RT. Our model accounted well for the observed behavior.

Next, we investigated whether patients with Parkinson's disease (PD) show altered motivational effects of reward. In both animals and humans, dopaminergic stimulation increases willingness to exert an effortful force for reward [29, 30] without trading speed for accuracy [31, 32]. In PD, dopamine depletion leads to slow, small movements. An attractive explanation for this is that PD patients experience greater costs for their movements [33, 34]. Indeed, it has been proposed that reward might potentially exert its effects on vigor of response via dopamine

[11, 32]. Applying our cost-of-control framework provides a parsimonious explanation for how this might arise. Patients with PD might be impaired in reducing internal noise in response to reward, manifested as a reduced ability to increase movement speed in response to incentive. This would explain why PD patients are sometimes less precise in motor tasks [15] yet can still generate a range of movement speeds [15]. To examine the proposition that dopamine depletion might increase the cost of attenuating noise, we tested patients with PD and fitted the model to healthy participants' and patients' data.

RESULTS

Modeling Reward Incentives in an Optimal Control Framework

Current Conceptual Frameworks Cannot Explain Behavior

When reward is available, we react faster [13, 32]. To explain how reward induces urgency, or time pressure in responding, it has been suggested that a high ongoing rate of reward may encourage fast frequent responding, minimizing opportunity cost [11]. Time pressure can be expressed in terms of temporal discounting [10, 12], in which a delayed reward is worth less. A commonly observed pattern of devaluation over time is "hyperbolic discounting" [10, 13], in which rewards delayed by time T are worth less by a factor of $1 + kT$, where k is known as the discount rate [35].

Conversely, at least two factors favor *slower* movements. First, fast movements require more energy. Within the framework of motor control, a fast movement results from a larger "control command"—e.g., the firing rate of a motor neuron. The energy expenditure corresponds to a cost, which is presumed to be related to size of the control command [36, 37]. Second, motor noise has been assumed to increase proportionally to the size of the control command [26], such that faster actions are less likely to be successful (e.g., arrive on a target).

These constraints lead to an optimum speed, since faster movements have higher energetic costs and error rates, which must be "paid" by gaining more reward sooner. To quantify this, we consider a motor command $\mathbf{u}(t)$, representing a set of instructions varying over time, for example, the neural output to muscles. We may then weigh up the expected value (EV) of an action, which depends on $\mathbf{u}$ [36] (Figure 1B). The optimal movement speed can be determined by finding $\mathbf{u}$ that maximizes EV. The utility of the reward R is scaled by the probability of the movement arriving on-target P_{win} and must balance the energetic cost $|\mathbf{u}|^2$:

are, however, subject to greater neural noise [7, 23]. Thus, according to previous accounts, increasing the reward shifts behavior from cautious, accurate responding to impulsive, inaccurate responding [37] (Figure 1A, blue line). Crucially, this form of cost function does not permit both accuracy *and* movement speed to increase simultaneously without compromising movement amplitude. To account for such effects, it is necessary to invoke a second dimension of control: a cost for attenuating motor noise, or equivalently, increasing signal-to-noise ratios. Put simply, we might choose to invest in noise reduction, if it were advantageous (Figure 1C). To make the cost explicit, we can include in $\mathbf{u}$ an additional control signal that reduces noise.

Applying a Noise-Reduction Cost to Motor Execution

We now consider a simple one-dimensional movement and split the command $\mathbf{u}$ into two components: a standard motor command u_F signaling force, and also a novel precision control signal u_P. For the optimal movement, a force/precision pair $\mathbf{u} = [u_F, u_P]$ must be chosen that maximizes value (Figure 1D). The larger the precision command u_P, the lower the resultant noise in the force generated by u_F. The actual mechanism that cancels noise might be complex, e.g., involving numerous internal signals, but our notion of a precision signal abstracts away the actual signals that correct for noise and retains only their cost and efficacy. (See Supplemental Experimental Procedures for a general form and discussion on how noise might be attenuated in the brain.) The probability of obtaining the reward $P_{win}(\mathbf{u})$ will increase with precision u_P and decrease with force u_F. But because we treat u_P in the same way as a control signal, it incurs a cost $|u_P|^2$.

When motor noise and accuracy are made irrelevant (e.g., for very large targets), then increasing reward simply increases the cost of time relative to energetic costs. Subjects are consequently more willing to exert more effort to move faster, so higher reward increases optimal speed (Figure 2A), as in the orthodox view [16, 36]. Conversely, if speed is ignored and only accuracy and precision are considered, then a new trade-off occurs between the cost of precision and the cost of errors. Since precision improves the probability of success but is expensive, there is an optimal level of accuracy which increases when more reward is on offer (Figure 2B). Crucially, when both precision and force are allowed to vary simultaneously, reward has the effect of increasing the optimal velocity while also reducing motor variability (Figure 2C). For each reward level R, there is a particular combination of force u_F and precision u_P that maximizes EV (Figures 3A and 3B), corresponding to an optimal saccade velocity and endpoint variability (Figures 3C and S5). The optimum will depend on an individual's temporal discount

$$EV(\mathbf{u}) = R \times D(\mathbf{u}) \times P_{win}(\mathbf{u}) - |\mathbf{u}|^2$$

$$\text{Expected value of action} = \text{Reward} \times \text{temporal discount} \times \text{probability of reward given a motor command} - (\text{size of motor command})^2 \qquad \text{Equation (1)}$$

In the orthodox model, higher reward increases the relative importance of time costs D, relative to energetic costs $|\mathbf{u}|^2$. High reward thus favors fast movements (larger $\mathbf{u}$) [12, 13], which

rate k and the noise parameter σ. To account for the possibility that not all noise may be controllable by a system (e.g., noise in the effector itself), an additive baseline noise term σ_0 can be

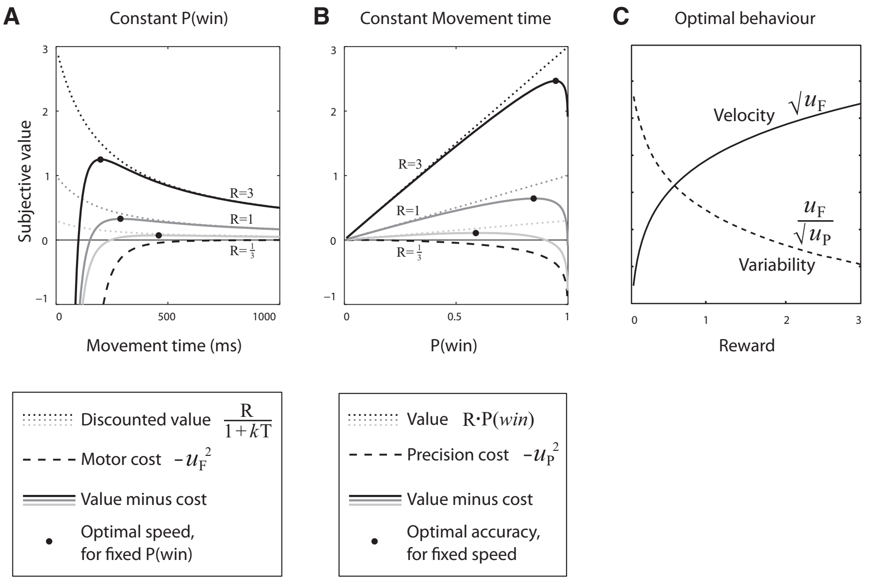

Figure 2. The Costs of Inaccuracy, Slowness, and Control

Our model incorporates three costs: inaccuracy is expensive because errors are not rewarded, slowness is expensive because a reward is less valuable when delayed (temporal discounting), and we further suggest that control over errors is itself expensive. Therefore, a three-way balance obtains.

(A) The vertical axis represents the subjective value of a given movement. For a given reward R, temporal discounting causes the reward's subjective value to fall as movement times get longer (dotted lines). However, moving faster entails greater energy expenditure (dashed line; negative value implies a cost). The net value (solid lines) is the sum of these two components, showing that the optimal movement is faster with higher reward [36].

(B) The probability of winning a reward, P_{win}, could depend on the endpoint of the movement being accurate. The cost of precision allows the endpoint variability to be reduced at a cost. The probability of landing on a fixed-size target can be increased if a "precision cost" is paid (dashed line). Precision increases the average gain from winning (dotted line), as shown for three different reward levels. The net value (solid lines) illustrates that the optimal movement is more precise with increasing reward.

(C) If both speed and accuracy are both free to vary, the optimum pair can be determined as a function of reward. Reward increases the optimal movement speed and, when temporal discounting is not too large, reduces the optimal endpoint variability.

included. In this case, σ_0 represents a participant's fixed motor noise, whereas σ represents the relative cost of precision, compared to energetic (force) cost.

Application of Precision Control Costs to Rise-to-Threshold Models

Controlling noise might be relevant not only for online motor control but also for deciding which action to take, and when. For decisions, standard speed-accuracy trade-offs are accurately predicted by rise-to-threshold models such as the drift-diffusion model (Figure 4A). In this model, a decision variable accumulates information over time about which action to select. When the evidence reaches a threshold, an action is triggered. Lowering or raising the decision threshold θ gives rise to fast, error-prone choices or slow, accurate responses, respectively [9, 38]—trading speed for accuracy. By default, the signal-to-noise ratio μ/σ is assumed to remain constant. Attention or alertness might augment the gain of signal over noise, but this is often postulated to be "effortful," currently without a quantitative prediction [25]. We suggest that these factors might be described in terms of a top-down control signal u_P that improves the signal-to-noise ratio in the accumulator [39]. Crucially, this noise-reduction signal may carry costs, which increase with u_P. The threshold/precision pair $\mathbf{u} = [\theta, u_P]$ may then be optimized to maximize value (see Supplemental Experimental Procedures). The model predicts that reward could improve the signal-to-noise ratio of decisions when it is economically feasible. The control cost determines, for the first time quantitatively, how motivation leads to fast, accurate responses—i.e., "true improvement" in performance. Simulations of drift diffusion were run to obtain the optimum threshold and precision for various reward levels and signal-to-noise ratios. These simulations showed that reward increased accuracy (Figures 4B and 4D), but also shortened RTs, under conditions when signal-to-noise μ/σ was high (Figure 4E, red lines).

Testing the Effects of Reward Using Saccades

We devised a novel saccadic task to measure how reward impacts upon both speed and error in movements and decisions. Trials started with participants fixating one of three gray discs arranged in a triangle (Figure 5A). They were instructed to move their eyes as fast as possible to the disc that lit up second. Participants were told that the first disc that was illuminated would be a distractor and the second would be the target. The faster they arrived at the target, the more money they won. Critically, during the 1.2 s foreperiod, a recorded voice was played back, speaking the maximum reward available on this trial. Three reward levels were used: 0 pence (p), 10p, or 50p (1p $\approx$ 1.5 US cents). This indicated the amount that could be won if a saccade was made rapidly to the target. Next, the fixation disc was dimmed while one of the other discs was brightened (the distractor). After 80 ms, the remaining disc (the target) brightened also. The display remained until gaze arrived at the target. The task is a variant of the double-step paradigm [40] and aimed to maximize oculomotor capture by the salient distractor [41].

Reward was calculated adaptively on each trial dependent on when gaze arrived at the target and was displayed numerically (Figure 5B). The target location was then used as the starting point for the next trial. Participants performed 72 trials of each of the three reward levels, intermixed. The task yielded four performance measures: oculomotor capture errors (classified offline according to whether the first saccade endpoint was closer to the distractor than the target; Figure 5C), RT measured as time from distractor onset until initiation of saccade, peak velocity of correct saccades, and the variability in amplitudes of this first saccade. This gave two measures of speed, and two measures of accuracy, for the motoric and target-selection aspects of the task (Figure 5D).

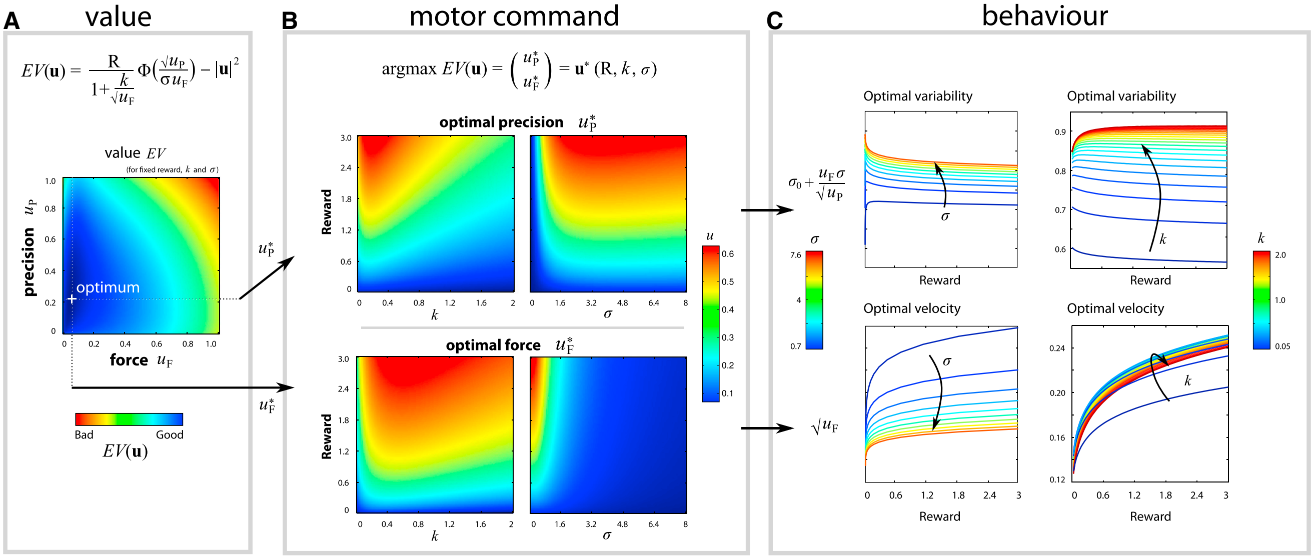

Figure 3. Optimal Control Model to Explain the Effect of Reward Incentives

In order to account for the ability of reward to improve both speed and accuracy, we hypothesized that in addition to a "vigor" or force signal (u_F) that determines a movement's speed, individuals are also able to select a "precision" signal (u_P) that determines the amount of variability in a movement. Crucially, this precision signal is also costly.

(A) Each given motor command, i.e., a pair of force and precision $\mathbf{u} = (u_F, u_P)$, has an EV. The image shows EV as a function of **u**, with the best combination as blue and worst as red. The value depends on three effects. First, the reward available is temporally discounted by the time taken by the movement, e.g., by hyperbolic discounting $1/(1+k/\sqrt{u_F})$. Second, this reward is only obtained if the movement is on target. We assume a Gaussian variation Φ of the endpoint proportional to the size of the motor command. Third, although we can go faster to reduce temporal discounting (increasing u_F) and be more precise to reduce error (increasing u_P), both of these incur a cost proportional to the squared control signal, u^2. This leads to an optimal combination of force and precision for each movement, $\mathbf{u}^*$.

(B) The optimal motor command for a situation depends on the reward level R and on two subject-specific parameters: the discount rate k and the noise-control cost σ. The optimal precision (upper panels) and force (lower panels) both increase with increasing reward (y axis), indicating that reward induces greater "spending" on both speed and accuracy. However, precision and force are differentially influenced by reward, and the balance depends on the urgency (temporal discount, k, left panels) and error constraints (encapsulated by σ, right panels).

(C) The optimal commands determine the velocity and duration of each movement and the amount of variability for a desired movement amplitude. Reward always increases velocity (lower panels). However, variability may increase or decrease with reward (upper panels), depending on σ and k. A subject with minimal discounting (e.g., $k < 0.5$) becomes less variable with higher reward, whereas a subject with high discount rates (e.g., $k > 1$) in fact tends to become *more* variable with higher reward (upper panels) as they are under greater time pressure, i.e., trading speed for accuracy. These effects are re-plotted on different axes in Figure S5.

Reward Breaks through the Speed-Accuracy Trade-Off in Healthy People

In the first experiment, we studied the effects of reward in 39 healthy participants. Reward significantly increased speed, in terms of both faster saccade velocities and shorter RTs. In addition, it also improved accuracy, with reduced oculomotor capture rates and lower endpoint variability. With high incentives (50p), the average peak saccade velocity was of $474^\circ s^{-1} \pm 13^\circ s^{-1}$ (SEM) compared to $452^\circ s^{-1} \pm 11^\circ s^{-1}$ with no incentive (repeated-measures ANOVA, main effect of reward $F(2,76) = 20.8$, $p < 0.001$; Figure 6A). RTs were also significantly shorter with high incentives (271 ± 11 ms) compared to no incentives (281 ± 11 ms) (main effect of reward $F(2,76) = 5.30$, $p = 0.007$; Figure 6B). In addition, reward reduced saccadic endpoint variability ($F(1,77) = 5.02$, $p = 0.027$; Figure 6C). It also improved accuracy by reducing oculomotor (distractor) capture rate (arcsine-transformed $F(2,76) = 3.8$; $p = 0.026$; Figure 6D). The results for accuracy and RT are re-plotted in Figure 6E, showing clearly that reward pushes performance beyond the speed-accuracy trade-off, consistent with our model predictions. Further analysis revealed that the velocity increase could not be explained by larger amplitudes or reduced curvature (see Supplemental Experimental Procedures). A conditional accuracy function plot demonstrated that the earliest responses (around 200 ms) were prone to distraction (50%), whereas later responses were more accurate, as predicted by standard speed-accuracy trade-off (Figure 6F). However, reward shifted the curve upward and leftward, as predicted by applying a control cost to simulations of the drift-diffusion model (Figure S1B; Supplemental Experimental Procedures).

Across healthy participants, those who had the greatest increase in velocity also had the greatest decrease in motor variability, indicating stronger motivational effects ($r^2 = 0.23$, $p = 0.001$; Figure S4A). Faster individuals were also more precise ($r^2 = 0.094$, $p = 0.045$; Figure S4B). This is predicted by the model (Figure 4C, left panels), in that a participant with low control cost σ will be both fast and precise. Participants with faster velocities were also more sensitive to reward ($r^2 = 0.12$, $p = 0.021$; Figure S4C), which is also predicted by the model (Figure 4C, lower panels): an individual with higher temporal discount rate k or lower noise σ would have both a higher overall velocity and a steeper slope of velocity with reward. Interestingly, there was no correlation between reward's effects on velocity and RT, or between reward effects on motor endpoint variability and

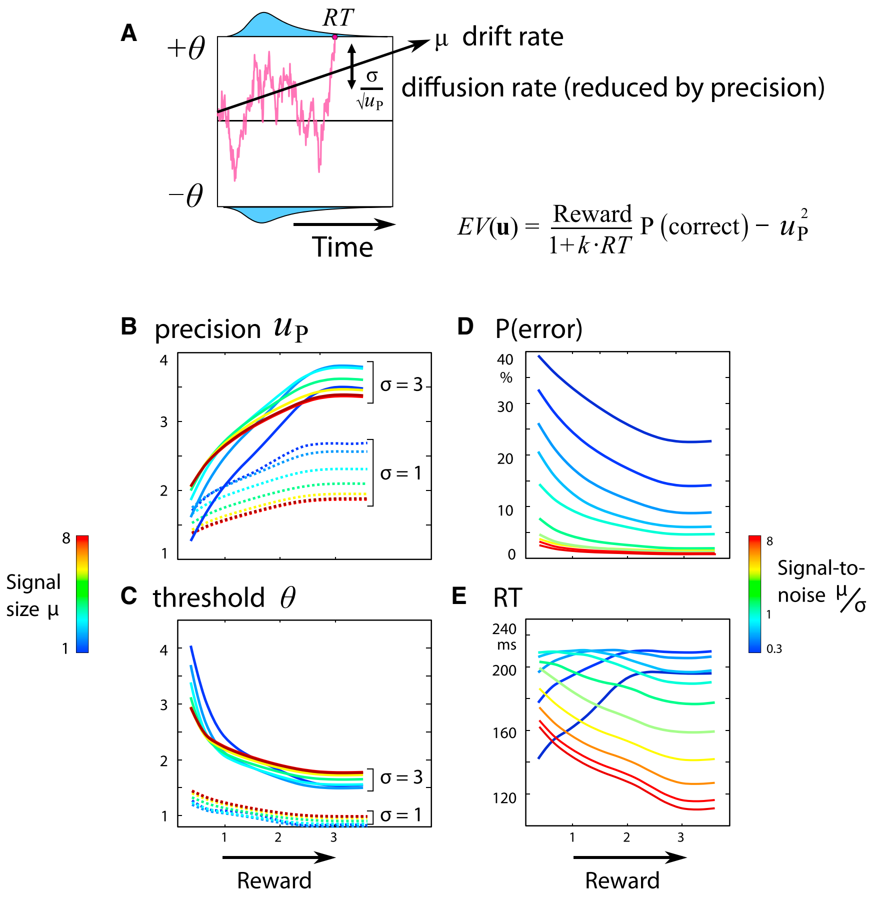

Figure 4. Cost of Control Applied to Drift Diffusion

(A) The drift-diffusion model assumes an accumulator integrating incoming information at a fixed drift rate (μ), subject to noise (σ), until it reaches a threshold (θ). The red line illustrates the trajectory in an example trial. Blue histograms indicate the distribution of response times for correct and incorrect responses. Increasing the threshold leads to more accurate decisions, at the cost of slower responses. In order to account for violations of the speed-accuracy trade-off, we introduced a costly noise-reduction parameter (u_P), similar to our extended motor control model. This permits the optimal combination of threshold and precision to be chosen.

(B–E) Simulations provide reaction times and accuracy (i.e., when the decision terminates, and whether it is at the positive or negative boundary) for a variety of signal sizes (μ), noise (σ), and reward levels (*R*). For each condition, the optimal pairing of threshold (θ) and precision (u_P) is selected to maximize value (EV). The value of a pair was calculated as accuracy multiplied by reward, temporally discounted by the reaction time.

(B and C) As reward increases, it is optimal to increase the precision and lower the decision threshold.

(D) This leads to improved accuracy with reward.

(E) When the signal-to-noise ratio is high, reward encourages faster responding; however, when the decision is noisy, reaction times actually increase with reward, despite falling thresholds—producing a speed-accuracy trade-off.

distraction error rates (Figures S4D and S4E), suggesting that cognitive and motor control costs might be optimized independently, in keeping with our two separate model formulations.

Cost of Control in Parkinson's Disease

To study the effect of dopaminergic dysfunction on motivation by reward, we compared 19 PD patients with 22 age-matched controls (Table S2), performing the same task as above (Figure 5). Patients had mild to moderate PD with no or minimal cognitive impairment. To compare patients and controls, we used a mixed-effects linear model, with factors disease and reward. There were no significant main effects of PD: patients had saccade velocities comparable to those of healthy age-matched control participants (Figure 7; PD versus control, $F(1,80) = 1.18$, $p > 0.05$) and did not make more oculomotor capture errors than controls (mean 24.8% errors in PD compared to 27.7% in controls, $F(1,80) = 0.29$, $p > 0.05$). There was a trend toward longer RTs than controls (364 ± 98 ms [SD], compared to 315 ± 66 ms for controls, $F(1,80) = 3.67$, $p = 0.063$). Critically, patients had shallower reward sensitivity slopes for velocity, RT, and error rate (interaction of disease × reward: $F(1,80) = 5.19$, $p = 0.025$ for velocity; $F(1,80) = 6.32$, $p = 0.014$ for RT; $F(1,80) = 4.98$, $p = 0.028$ for error rate), with a similar trend for endpoint variability ($F(1,80) = 0.32$, $p = 0.077$). These latter findings are consistent with reduced reward sensitivity in PD, as predicted by the precision-cost model, if the precision cost σ were increased (Figure 4E, compare red and blue lines). An analysis of just the PD group showed that patients increased their velocity significantly in response to reward (significant proportional change in velocity with reward, $F(1,37) = 5.39$, $p = 0.026$) and thus did modulate their behavior to some extent, although not to the degree of healthy controls. PD patients were not significantly influenced by reward, however, in terms of endpoint variability, RT, and oculomotor capture (all $p > 0.05$).

Because the model predicts that reward can either increase or decrease endpoint variability according to the individual, we performed a supplementary analysis of per-subject effects of reward (Figure S4A). Individual patients showed significant reward effects in different directions, and that effect of endpoint variability was correlated with baseline velocity, in line with the model (Figure S4B). Conditional accuracy functions also demonstrated absent reward effects in PD patients ($p > 0.05$) (Figure S1A). The effect of reward on RT was examined at different time points during the RT distribution (Figure S1C). In controls, responses occurring later in the RT distribution were the ones whose speed was increased the most by reward, as predicted by the simulation (Figure S1D), effects that were absent in PD. Fatigue over time could not explain the reduced reward sensitivity in PD (see Supplemental Experimental Procedures).

Cost of Control Explains Movement Velocity and Endpoint Variability

For each participant, velocity and variability as a function of reward were fitted to the motor control model, giving three free parameters for each subject: the temporal discount rate *k*,

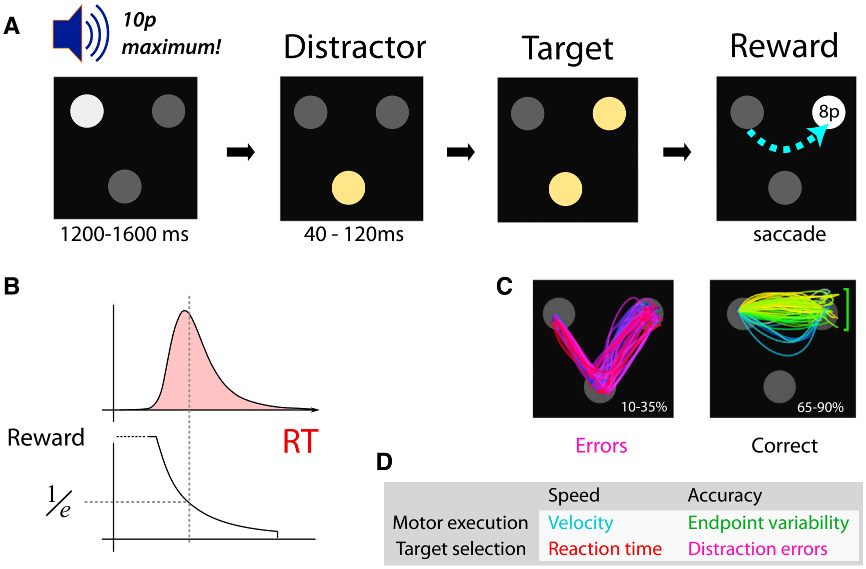

Figure 5. Oculomotor Capture Task with Trial-wise Incentives

(A) Three equidistant discs were dimly illuminated. At the start of each trial, participants had to fixate one disc, which was brightened. A recorded voice gave an auditory reward cue of "0p maximum," "10p maximum," or "50p maximum," which indicated the maximum amount of money that could be won if participants were fast to look at the target on that trial. After a variable foreperiod, the other two discs were illuminated asynchronously, with a delay of 40 to 120 ms. Participants were instructed to look as fast as possible to the second disc. Thus, the first onset acted as an early onset distractor, and the second disc indicated the target.

(B) After gaze arrived at the target, participants were rewarded according to reaction time. Reward was calculated as a fraction of the maximum available, using an exponential falloff. The falloff was determined adaptively using quantiles of the last 20 trials, in order to maintain the difficulty level over the course of the experiment.

(C) On approximately 30% of trials, gaze was "captured" by the distractor (errors), resulting in a brief saccade to the first disc, followed by a corrective saccade to the target. The trajectory of gaze was classified according to whether the first saccade terminated on the target or on the distractor. Correct trials exhibited a variety of curvatures; each trial is colored according to the initial direction of the eye velocity.

(D) The task provided four measures of performance. Both speed and accuracy could be examined for motor execution of the saccade and for selection of the correct target.

the noise-control cost σ, and baseline noise σ_0. These three parameters determine the optimum velocity $(\sqrt{u_F})$ and variability $(\sigma_0 + \sigma u_F/\sqrt{u_P})$ as a function of reward (Figure 3).

Compared to controls, PD patients had significantly increased noise-control costs σ (two-tailed unpaired t test, $t(36) = 2.21$, $p = 0.034$; Table S1). Neither their temporal discount rate nor their baseline variability was significantly different from healthy people ($p > 0.05$). One interpretation of the data is that PD patients go slower in order to reduce their motor variability in the face of an increased cost for controlling internal noise. The cost-of-control model fitted the data better than simpler models in which only the force or precision were allowed to vary with reward (ΔAIC = 6.5; Table S3; "Model Comparison" in Supplemental Experimental Procedures).

DISCUSSION

Standard optimal control theory constrains human performance to be bounded by an upper limit. Motivation by reward is remarkable for improving performance beyond its normal bounds. To account for this, we devised a variant of optimal control theory that incorporates a precision signal that allows noise to be attenuated. But importantly, precision comes at a cost—the cost of control (Figure 1). In the motor domain, our model predicts that reward may improve both velocity and precision (Figure 3). In the decision domain, it predicts faster and more accurate choices with higher reward (Figure 4).

We tested this using a novel incentivized saccadic task (Figure 5). In accordance with our model, reward increased saccadic velocity and endpoint accuracy, and reduced RTs and oculomotor distractibility (Figure 6). By allowing each participant to optimize behavior according to their own noise and temporal discounting, the model was able to accommodate individual differences in responses to reward across the populations, better than simpler models.

Applying optimality to reward incentivization unites recent conceptions of motivation [20, 24] with existing mathematical frameworks of optimal action [8, 11, 42]. If reward is held fixed, our model reduces to previous accounts [10, 36], but if reward is altered, parallel shifts can occur that violate the classical speed-accuracy trade-off (Figure 1C), at least when signal-to-noise ratios are high and temporal discounting is small (Figures 3C and 4E). Such effects are often reported as attentional improvements in cognitive control tasks [5, 6] but have not previously been quantified in terms of cost-benefit analysis.

Previous presentations of the drift-diffusion model have incorporated speeding up of decisions by reward [42], but our addition of a control cost makes new predictions for the drift rate. Neuronal ramping activity preceding a decision has been interpreted in terms of drift diffusion, but existing models fail to capture how emphasizing speed over accuracy may increase the peak firing rates at the moment of decision [43]. Unlike previous attempts, our model does predict faster RTs accompanied by higher thresholds, under specific circumstances (Figures 4C and 4E).

In both animals and humans, dopamine is considered to have a crucial role in mediating response vigor [11, 32] and in overcoming internal costs associated with particular behaviors [29]. Individuals with PD, a condition associated with dopamine depletion, had reduced sensitivity to reward on speed measures compared to age-matched controls (Figure 7), yet they maintained similar overall levels of accuracy. In the model, this corresponded to a greater cost of controlling noise. The results are in line with previous evidence that, for a matched speed, PD patients' movements are less accurate [44] and cognitive control errors more frequent [45].

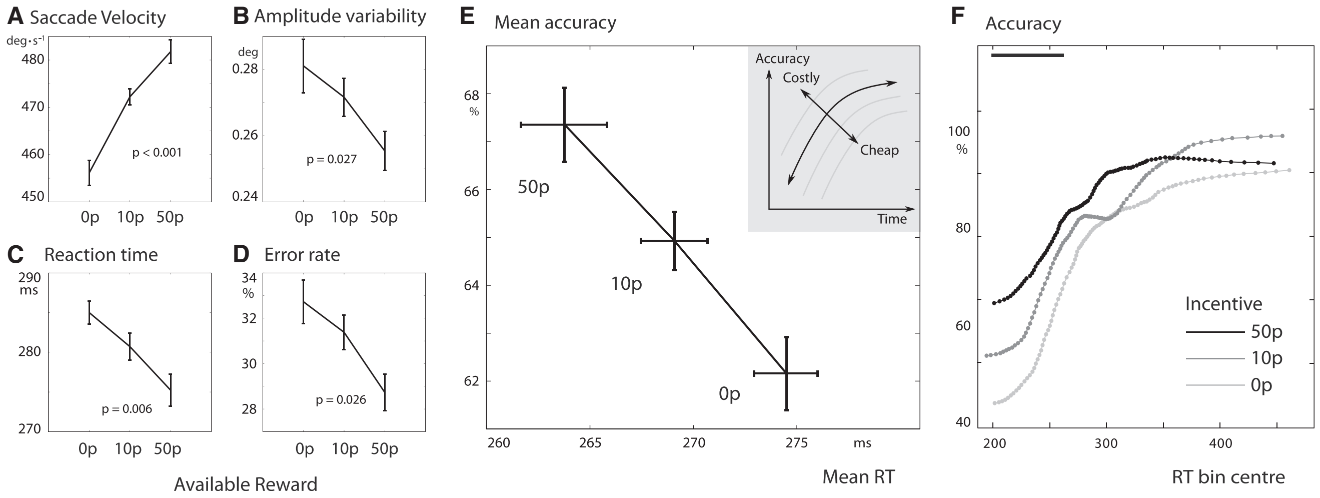

Figure 6. Effects of Reward on Saccades in Healthy Participants

(A) For correct trials, the mean peak velocity of saccades increased with higher incentives, demonstrating invigoration by reward. Error bars indicate within-subject standard error.

(B) Endpoint variability (standard deviation of the saccade amplitudes) in each condition became less variable with increasing incentives, indicating that reward can improve motor precision.

(C) For correct trials, RTs were faster for higher incentives.

(D) The rate of oculomotor capture (proportion of trials on which the first saccade after the onset was directed to the distractor, i.e., error trials) was reduced with increased incentives, indexing improved accuracy. Error timings are shown in Figure S2 and times to correct errors in Figure S3.

(E) Plotting the data from Figure 5 as accuracy versus RT (where accuracy is defined as percentage of responses that were directed to the target and not to the distractor) demonstrates how, with increasing incentives, reaction time decreased and accuracy simultaneously improved. The inset shows how this relates to Figure 1C: the speed-accuracy trade-off is broken.

(F) Conditional accuracy plot shows how, for a fixed reward level, accuracy improved with increasing RT, but this relationship was shifted by incentives, with the greatest differences evident at short RTs. The gradient of each curve is always positive, indicating that for a trials within a single reward level (i.e., constant incentive), the speed-accuracy trade-off held. The plot shows the proportion of saccades that went to the target, in a sliding window along the RT distribution, width 20% quantiles. Patient data and model are shown in Figure S1.

Could the loss of reward sensitivity in PD be explained simply by patients performing at their ceiling? This seems unlikely. First, the PD patients were not entirely unresponsive to reward. Second, at fast RTs PD patients are in fact *more* accurate than controls; however, at slower RTs the accuracy plateaus lower (Figure S1A). This suggests that instead of being uniformly slow, patients maintain a stable accuracy level at the cost of speed [46]. Finally, in PD, reward speeded up slow responses similarly to fast responses (Figure S1C), whereas with ceiling effects, slow saccades might be expected to show greater motivational improvement.

Although dopaminergic reward signals are well characterized, their role in weighing costs against benefits remains obscure. Our results are suggestive, but not conclusive, that dopamine depletion may lead to a higher cost of control. Dopamine might facilitate motivational performance adjustments due to its neuromodulatory effects on synaptic noise or gain [47], potentially reducing the cost of control. However, from this study alone, it is not possible to determine for certain which specific mechanisms mediated the effects we observed. Although our patients had mild to moderate PD without dementia, we cannot rule out pathology in non-dopaminergic systems.

What Is the Real Cost of Reducing Noise?

If control signals *can* truly attenuate noise, then why are we not built to exercise maximal control at all times? There are at least three possible reasons why control should be expensive: opportunity costs, neural resources, and entropy.

First, the "noise" that needs to be attenuated in the brain might in fact be constituted by *potentially* relevant but currently irrelevant signals. Distraction confers ecological advantages, and ignoring distractors could be costly or dangerous. For the motor system, analogously, producing precise movements entails isolating the motor system from competing affordances. Selective attention and precision thus carry danger or opportunity costs. Second, controlling noise might require allocation of more "neural resources," for example more neurons in population codes [23], higher firing rates (Figure S6), or the reduction of motor error by co-contraction of antagonistic muscles, which increases effector stiffness but incurs an energetic cost. Finally, any feedback-control signal that maintains stability in the face of thermal noise will inherently increase the entropy of a system [48], which must be dissipated as heat [49]. Consequently, minimizing control signals may be a central principle of brain design.

Whatever the real cost of control, its estimation and optimization by the brain can be summarized by the equations presented here. Cost-benefit optimization then directly predicts the observed effects of reward on speed and accuracy. We suggest that the mathematical formulations of optimal control theory, complemented by our costly noise-reduction signal, would be broadly applicable to any domain in which behavioral performance is limited by neuronal noise or resources. If combined with an appropriate model of how noise degrades performance, our formulation might also predict motivation's effects on more

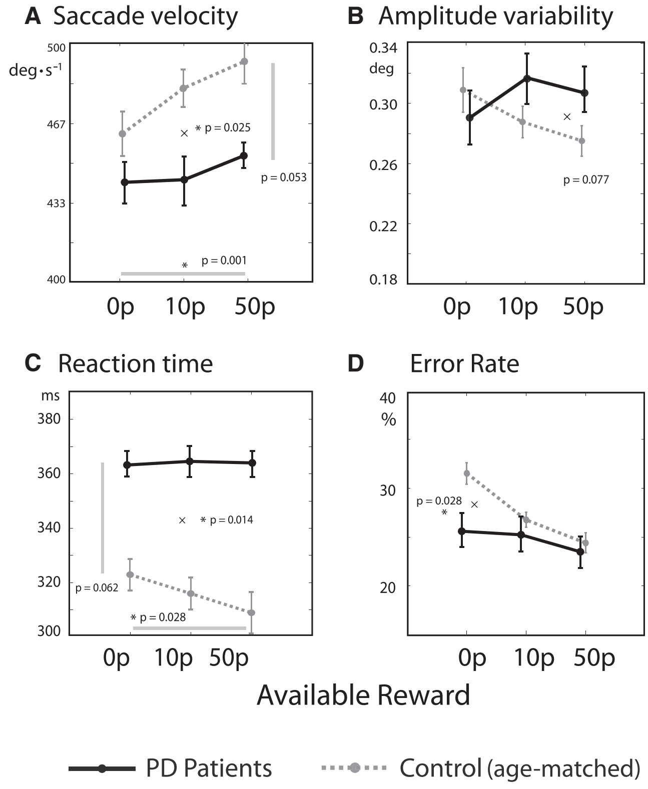

Figure 7. Reduced Reward Sensitivity in Patients with Parkinson's Disease

(A) PD patients had decreased reward sensitivity, as demonstrated by a shallower slope. This is consistent with impaired invigoration by reward. Overall velocities were also marginally slower.

(C) Saccadic amplitude variability was not significantly abnormal in PD.

(B) Reaction times were slower in PD and showed reduced reward sensitivity.

(D) Patients showed weaker effects of reward on improving distractibility, as measured by oculomotor capture (i.e., they did not reduce their error rate in response to incentive), compared to controls. Between-subject correlations are shown in Figure S4.

complex aspects of behavior, such as attentional selection, working memory, and inhibitory control.

EXPERIMENTAL PROCEDURES

Application of Precision Cost to Motor Commands

Equation 1 indicates the considerations in evaluating an action. To express P_{win} and temporal discounting D as a function of the control command $\mathbf{u}$, we first assume hyperbolic temporal discounting, $D(\mathbf{u}) = 1/(1 + kT(\mathbf{u}))$ [12]. Second, accuracy $P_{win}(\mathbf{u})$ depends on the amount of motor noise, which is usually assumed to be Gaussian, and proportional to $|\mathbf{u}|$ [26]. Reward will be missed if noise exceeds some threshold. The probability of landing within a unit radius is given by the cumulative normal error function (denoted Φ). This gives an equation for the "orthodox view" (Figure 1B),

$$EV(\mathbf{u}) \propto \frac{R}{1 + k \cdot T(\mathbf{u})} 2\Phi\left(\frac{1}{\sigma \cdot |\mathbf{u}|}\right) - |\mathbf{u}|^2,$$

where the parameter k indicates a subject's temporal discount rate and σ denotes their motor noise.

For a simple one-dimensional movement, we find the optimal force/precision pair $\mathbf{u} = [u_F, u_P]$. We assume that noise is scaled down by precision, and for our specific motor task, we write the noise as $\sigma u_F / \sqrt{u_P}$ (see Supplemental Experimental Procedures). Furthermore, movement time depends on the force component of the command, with $T(\mathbf{u}) \propto 1/\sqrt{u_F}$. Finally, since we treat u_P as a control signal, it contributes to the cost $|\mathbf{u}|^2$, alongside the force. This gives the expected value (EV) of a command (Figure 1D):

$$EV(u_F, u_P) \propto \frac{R}{1 + k/\sqrt{u_F}} 2\Phi\left(\frac{\sqrt{u_P}}{\sigma \cdot u_F}\right) - |u_F|^2 - |u_P|^2.$$

Application of Noise-Reduction Cost to Cognitive Control: Drift-Diffusion Model

The drift-diffusion model allows us to predict the RT distribution and error rate of a two-alternative choice. The outcome of the decision depends on the average rate of accumulating information μ, the threshold θ at which enough information is available to make a decision, and σ, the amount of noise in the accumulator (Figure 4A). We suggest that an organism can control not only the threshold, but also decision noise, to optimize EV. Noise can be reduced by a precision signal to give an effective noise level $\sigma/\sqrt{u_P}$. This precision entails a cost $|\mathbf{u}|^2 = u^2_P$. In an alternative race model framework, the rate of rise might be increased (Figures S2 and S3).

The time taken (T = RT) and accuracy P_{win} are calculated by simulating the diffusion process. We assume hyperbolic temporal discounting of reward, with $D(\theta, u_P) = 1/(1 + kT)$. These values are substituted into Equation 1. The optimum threshold and precision $[\theta, u_P]$ can then be found by simulation, which in turn determine speed and accuracy (Figures 4B–4E). Performance therefore depends on the reward on offer, the individual's baseline signal-to-noise ratio σ, and their temporal discount rate. High reward emphasizes time pressure but also encourages investment in precision—enabling the classical speed-accuracy trade-off to be broken by motivation.

SUPPLEMENTAL INFORMATION

Supplemental Information includes six figures, three tables, and Supplemental Experimental Procedures and can be found with this article online at http://dx.doi.org/10.1016/j.cub.2015.05.038.

AUTHOR CONTRIBUTIONS

S.G.M. designed, ran, and analyzed the model and experiments. T.T.-J.C., A.B., M.S., K.P.B., P.R.J., and M.H. recruited patients for the study. S.G.M, M.H., and M.A.J.A. wrote the manuscript.

ACKNOWLEDGMENTS

This research was funded by a Wellcome Trust Principal Fellowship to M.H. (WT098282) and a Wellcome Trust Research Training Fellowship to S.G.M. (WT090201MA). We are grateful to Rafal Bogacz and Sean Fallon for helpful suggestions and discussions.

Received: February 9, 2015
Revised: April 7, 2015
Accepted: May 19, 2015
Published: June 18, 2015

REFERENCES

1. Fitts, P.M. (1966). Cognitive aspects of information processing. 3. Set for speed versus accuracy. J. Exp. Psychol. *71*, 849–857.
2. Heitz, R.P. (2014). The speed-accuracy tradeoff: history, physiology, methodology, and behavior. Front. Neurosci. *8*, 150.
3. Shmuelof, L., Krakauer, J.W., and Mazzoni, P. (2012). How is a motor skill learned? Change and invariance at the levels of task success and trajectory control. J. Neurophysiol. *108*, 578–594.
4. Takikawa, Y., Kawagoe, R., Itoh, H., Nakahara, H., and Hikosaka, O. (2002). Modulation of saccadic eye movements by predicted reward outcome. Exp. Brain Res. *142*, 284–291.
5. Hübner, R., and Schlösser, J. (2010). Monetary reward increases attentional effort in the flanker task. Psychon. Bull. Rev. *17*, 821–826.

6. Krebs, R.M., Boehler, C.N., Egner, T., and Woldorff, M.G. (2011). The neural underpinnings of how reward associations can both guide and misguide attention. J. Neurosci. *31*, 9752–9759.

7. Harris, C.M., and Wolpert, D.M. (1998). Signal-dependent noise determines motor planning. Nature *394*, 780–784.

8. Bays, P.M., and Wolpert, D.M. (2007). Computational principles of sensorimotor control that minimize uncertainty and variability. J. Physiol. *578*, 387–396.

9. Bogacz, R., Wagenmakers, E.-J., Forstmann, B.U., and Nieuwenhuis, S. (2010). The neural basis of the speed-accuracy tradeoff. Trends Neurosci. *33*, 10–16.

10. Shadmehr, R., Orban de Xivry, J.J., Xu-Wilson, M., and Shih, T.-Y. (2010). Temporal discounting of reward and the cost of time in motor control. J. Neurosci. *30*, 10507–10516.

11. Niv, Y., Daw, N.D., Joel, D., and Dayan, P. (2007). Tonic dopamine: opportunity costs and the control of response vigor. Psychopharmacology (Berl.) *191*, 507–520.

12. Shadmehr, R. (2010). Control of movements and temporal discounting of reward. Curr. Opin. Neurobiol. *20*, 726–730.

13. Haith, A.M., Reppert, T.R., and Shadmehr, R. (2012). Evidence for hyperbolic temporal discounting of reward in control of movements. J. Neurosci. *32*, 11727–11736.

14. Chen, L.L., Hung, L.Y., Quinet, J., and Kosek, K. (2013). Cognitive regulation of saccadic velocity by reward prospect. Eur. J. Neurosci. *38*, 2434–2444.

15. Mazzoni, P., Hristova, A., and Krakauer, J.W. (2007). Why don't we move faster? Parkinson's disease, movement vigor, and implicit motivation. J. Neurosci. *27*, 7105–7116.

16. Xu-Wilson, M., Zee, D.S., and Shadmehr, R. (2009). The intrinsic value of visual information affects saccade velocities. Exp. Brain Res. *196*, 475–481.

17. Bijleveld, E., Custers, R., and Aarts, H. (2010). Unconscious reward cues increase invested effort, but do not change speed-accuracy tradeoffs. Cognition *115*, 330–335.

18. Dixon, M.L., and Christoff, K. (2012). The decision to engage cognitive control is driven by expected reward-value: neural and behavioral evidence. PLoS ONE *7*, e51637.

19. Holmes, P., and Cohen, J.D. (2014). Optimality and some of its discontents: successes and shortcomings of existing models for binary decisions. Top. Cogn. Sci. *6*, 258–278.

20. Shenhav, A., Botvinick, M.M., and Cohen, J.D. (2013). The expected value of control: an integrative theory of anterior cingulate cortex function. Neuron *79*, 217–240.

21. Todorov, E. (2005). Stochastic optimal control and estimation methods adapted to the noise characteristics of the sensorimotor system. Neural Comput. *17*, 1084–1108.

22. Seung, H.S., and Sompolinsky, H. (1993). Simple models for reading neuronal population codes. Proc. Natl. Acad. Sci. USA *90*, 10749–10753.

23. Faisal, A.A., Selen, L.P.J., and Wolpert, D.M. (2008). Noise in the nervous system. Nat. Rev. Neurosci. *9*, 292–303.

24. Kurzban, R., Duckworth, A., Kable, J.W., and Myers, J. (2013). An opportunity cost model of subjective effort and task performance. Behav. Brain Sci. *36*, 661–679.

25. Sarter, M., Gehring, W.J., and Kozak, R. (2006). More attention must be paid: the neurobiology of attentional effort. Brain Res. Brain Res. Rev. *51*, 145–160.

26. Harris, C.M., and Wolpert, D.M. (2006). The main sequence of saccades optimizes speed-accuracy trade-off. Biol. Cybern. *95*, 21–29.

27. van Beers, R.J. (2007). The sources of variability in saccadic eye movements. J. Neurosci. *27*, 8757–8770.

28. van Beers, R.J. (2008). Saccadic eye movements minimize the consequences of motor noise. PLoS ONE *3*, e2070.

29. Salamone, J.D., and Correa, M. (2002). Motivational views of reinforcement: implications for understanding the behavioral functions of nucleus accumbens dopamine. Behav. Brain Res. *137*, 3–25.

30. Wardle, M.C., Treadway, M.T., Mayo, L.M., Zald, D.H., and de Wit, H. (2011). Amping up effort: effects of d-amphetamine on human effort-based decision-making. J. Neurosci. *31*, 16597–16602.

31. Winkel, J., van Maanen, L., Ratcliff, R., van der Schaaf, M.E., van Schouwenburg, M.R., Cools, R., and Forstmann, B.U. (2012). Bromocriptine does not alter speed-accuracy tradeoff. Front. Neurosci. *6*, 126.

32. Beierholm, U., Guitart-Masip, M., Economides, M., Chowdhury, R., Düzel, E., Dolan, R., and Dayan, P. (2013). Dopamine modulates reward-related vigor. Neuropsychopharmacology *38*, 1495–1503.

33. Mazzoni, P., Shabbott, B., and Cortés, J.C. (2012). Motor control abnormalities in Parkinson's disease. Cold Spring Harb. Perspect. Med. *2*, a009282.

34. Baraduc, P., Thobois, S., Gan, J., Broussolle, E., and Desmurget, M. (2013). A common optimization principle for motor execution in healthy subjects and parkinsonian patients. J. Neurosci. *33*, 665–677.

35. Kacelnik, A. (1997). Normative and descriptive models of decision making: time discounting and risk sensitivity. Ciba Found. Symp. *208*, 51–67, discussion 67–70.

36. Rigoux, L., and Guigon, E. (2012). A model of reward- and effort-based optimal decision making and motor control. PLoS Comput. Biol. *8*, e1002716.

37. Shadmehr, R., and Krakauer, J.W. (2008). A computational neuroanatomy for motor control. Exp. Brain Res. *185*, 359–381.

38. Simen, P., Cohen, J.D., and Holmes, P. (2006). Rapid decision threshold modulation by reward rate in a neural network. Neural Netw. *19*, 1013–1026.

39. Milosavljevic, M., Malmaud, J., Huth, A., Koch, C., and Rangel, A. (2010). The Drift Diffusion Model can account for the accuracy and reaction time of value-based choices under high and low time pressure. Judgm. Decis. Mak. *5*, 437–449.

40. Camalier, C.R., Gotler, A., Murthy, A., Thompson, K.G., Logan, G.D., Palmeri, T.J., and Schall, J.D. (2007). Dynamics of saccade target selection: race model analysis of double step and search step saccade production in human and macaque. Vision Res. *47*, 2187–2211.

41. Theeuwes, J., Kramer, A.F., Hahn, S., and Irwin, D.E. (1998). Our eyes do not always go where we want them to go: capture of the eyes by new objects. Psychol. Sci. *9*, 379–385.

42. Bogacz, R., Brown, E., Moehlis, J., Holmes, P., and Cohen, J.D. (2006). The physics of optimal decision making: a formal analysis of models of performance in two-alternative forced-choice tasks. Psychol. Rev. *113*, 700–765.

43. Heitz, R.P., and Schall, J.D. (2012). Neural mechanisms of speed-accuracy tradeoff. Neuron *76*, 616–628.

44. Rand, M.K., Stelmach, G.E., and Bloedel, J.R. (2000). Movement accuracy constraints in Parkinson's disease patients. Neuropsychologia *38*, 203–212.

45. Joti, P., Kulashekhar, S., Behari, M., and Murthy, A. (2007). Impaired inhibitory oculomotor control in patients with Parkinson's disease. Exp. Brain Res. *177*, 447–457.

46. Wylie, S.A., van den Wildenberg, W.P.M., Ridderinkhof, K.R., Bashore, T.R., Powell, V.D., Manning, C.A., and Wooten, G.F. (2009). The effect of speed-accuracy strategy on response interference control in Parkinson's disease. Neuropsychologia *47*, 1844–1853.

47. Kroener, S., Chandler, L.J., Phillips, P.E.M., and Seamans, J.K. (2009). Dopamine modulates persistent synaptic activity and enhances the signal-to-noise ratio in the prefrontal cortex. PLoS ONE *4*, e6507.

48. Lan, G., Sartori, P., Neumann, S., Sourjik, V., and Tu, Y. (2012). The energy-speed-accuracy tradeoff in sensory adaptation. Nat. Phys. *8*, 422–428.

49. Tomé, T. (2006). Entropy production in nonequilibrium systems described by a Fokker-Planck equation. Braz. J. Phys. *36*, 1285–1289.

Current Biology

Supplemental Information

Reward Pays the Cost of Noise Reduction in Motor and Cognitive Control

Sanjay G. Manohar, Trevor T.-J. Chong, Matthew A.J. Apps, Amit Batla, Maria Stamelou, Paul R. Jarman, Kailash P. Bhatia, and Masud Husain,

Supplemental Figures and Tables

Figure S1. The time course of reward's effects (related to Fig.6F)
Figure S2. Race model interpretation of data (related to Fig.6D)
Figure S3. Analysis of time to correct an error (related to Fig.6C)
Figure S4. Correlation between reward sensitivity measures in healthy participants (related to Fig.6A)
Figure S5. Effects of internal noise and temporal discount rate on velocity and motor precision (related to Fig.3C)
Figure S6. How much negative feedback signal is required to attenuate noise? (related to Fig.1D)

Table S1. Fitted model parameters for healthy participants and patients
Table S2. Demographics of participants
Table S3. Model comparison for the drift-diffusion model fit

Supplemental Experimental Procedures

Modeling the cost of control

1 .Previous approaches predict invigoration by reward
2. Feedback control signals could attenuate internal noise
3. Optimisation with a novel precision signal
4. How much does noise reduction cost?
5. Applying the cost of control to saccades
6. Drift diffusion simulation

Empirical quantification of reward's effect on speed and accuracy

1. Task Instructions
2. Task
3. Materials
4. Saccade analysis
5. PD Patients
6. Statistics
7. Velocity effects were not attributable to amplitude or curvature
8. Conditional accuracy functions and Delta plots
9. Fatigue could not explain reduced reward sensitivity in PD
10. Analysis of distractor-target delay and error trials

Model fit of reward's effects on motor control

1. Model comparison
2. Patient vs. control comparison

Supplemental References

Figure S1

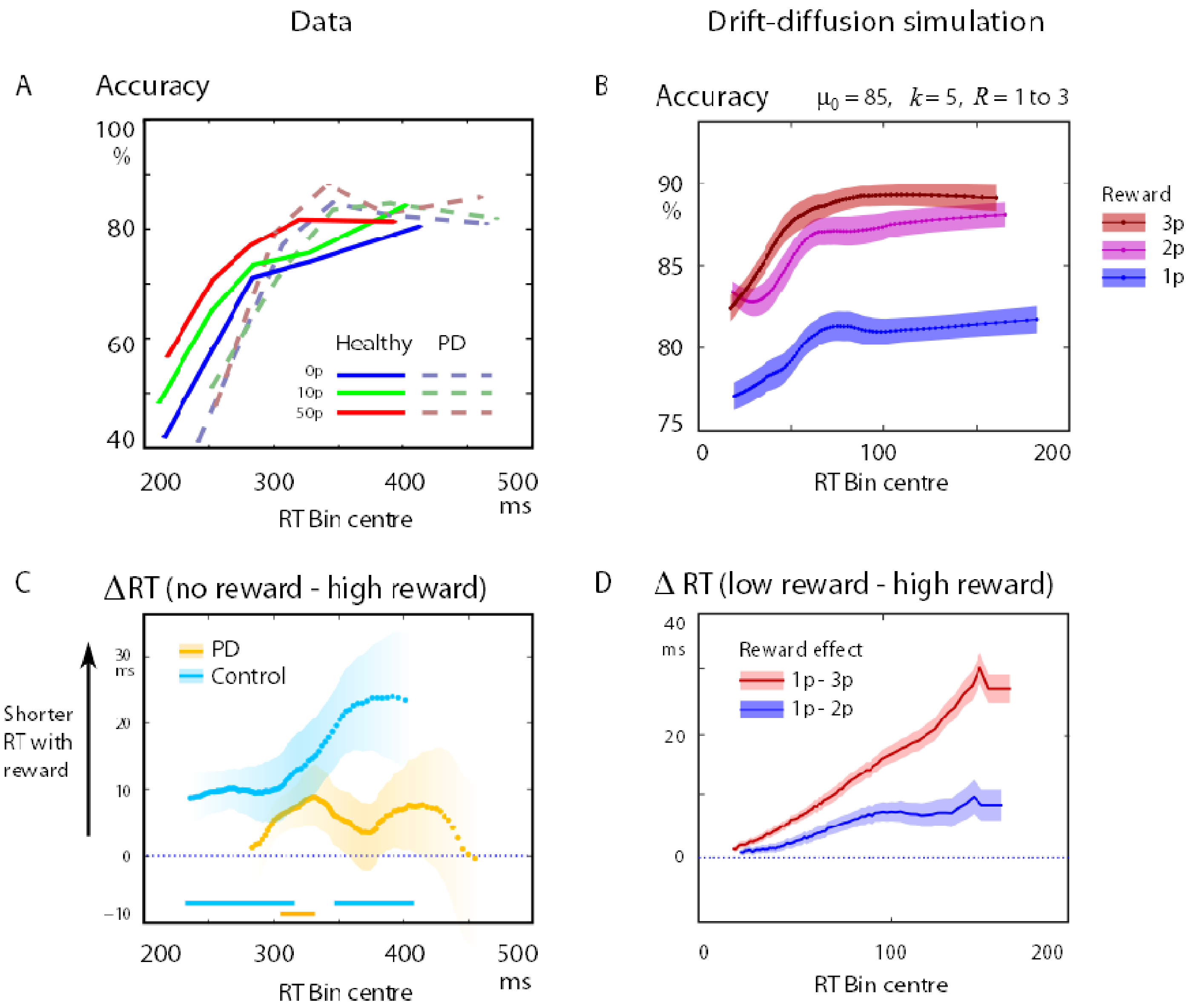

Figure S1: The timecourse of reward's effects (related to Fig.6F)

A) Conditional accuracy functions were constructed by binning reaction time into quintiles, and calculating the proportion of non-captured saccades in that bin, for each participant, and averaging across individuals. PD patients show a reduced reward effect, compared to age-matched controls, despite being more accurate overall. Shaded areas represent the standard error across individuals.

B) A drift-diffusion model was simulated to generate RT distributions for erroneous and correct responses. Those RTs can be plotted in the same way as data in panel **A**. Incorporating a cost for controlling noise in the accumulator allows the effect of reward to be predicted. Higher incentives lead to higher thresholds, but also higher signal-to-noise ratios. This results in a conditional accuracy function that shows an upward-and-leftward shift with incentive, similar to the data. Simulated subject parameters: signal = 85, baseline noise = 0.1, starting value variability 0.3, timestep 1 ms, discount rate 5 s^{-1}, reward 1 to 3 units, 500 trials. Threshold and precision chosen to maximise value, according to optimal control theory as described in **Supplementary methods**. Shaded area indicates standard deviation across 20 runs.

C) The effect of reward on RT was analysed for different RT bins, using a "delta plot". The plot was constructed by comparing each RT quantile in the high and low rewarded conditions, using a sliding window (See **Supplementary methods**). The curves shown are the average of (50p minus 10p) and (10p minus 0p), for each group. Healthy participants (blue) had a consistent effect of reward throughout the RT distribution, indicating that reward shortened reaction times by shifting the whole RT distribution. The effect of reward is weakest for early movements, and strongest for late movements. PD patients showed minimal effect of reward over the whole RT distribution. Bars below indicate $p<0.05$ by permutation test.

D) Simulated reaction times can be plotted to show the effect of reward as a function of RT, in the same way as data in panel **C**. The plots show that the effect of reward on RT is predicted to be largest on later responses, similar to the data.

Figure S2

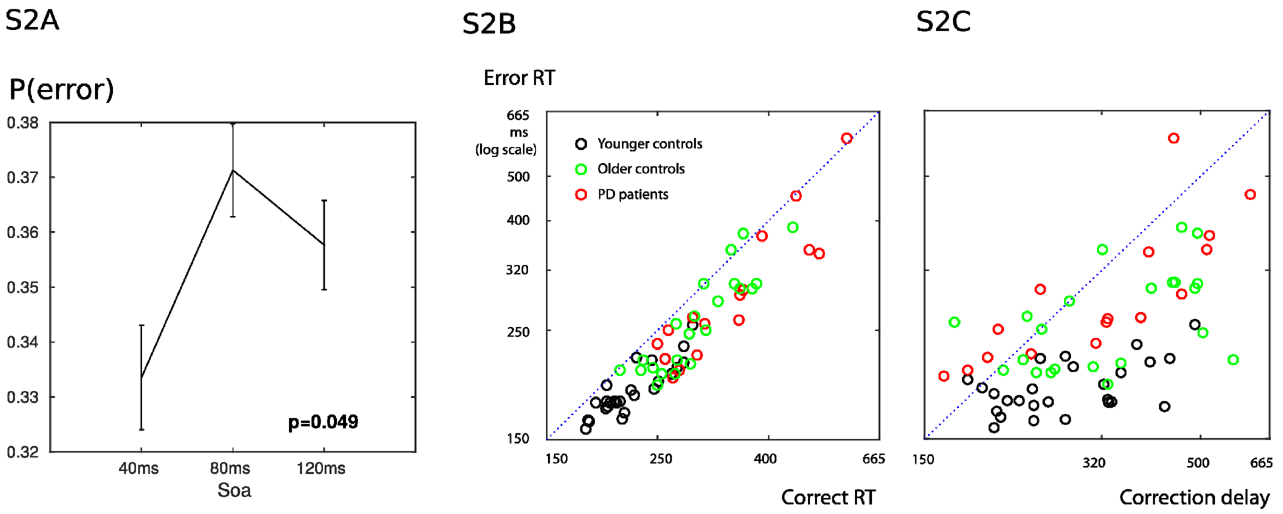

Figure S2: Race model interpretation of data (related to Fig.6D)

A) The interval between the distractor onset and the target onset (the SOA) was 40, 80 or 120 ms. The chance of a saccade to the distractor varied with SOA, compared to 40 ms (repeated measures 1-way ANOVA, main effect of SOA, $F(2,52)=3.20$, $p=0.049$; pairwise comparison 40 ms *vs*. 80 ms, $t=2.34$, $p=0.027$)

B) Error saccades to the distractor had a shorter latency than saccades that went directly to the target. For each participant we plotted the mean RT on error trials, against the mean RT on correct trials. On average, error RTs were shorter in all three groups (all $p<0.001$).

C) Error corrections occurred after each error saccade, as participants were required to look at the target before progressing to the next trial. The "correction delay" was taken to be the interval between the initiation of the error and the initiation of the first subsequent corrective saccade which landed on the target. Participants with longer error RTs tended to take longer to correct their errors (younger: $r^2=0.29$, $p=0.004$; older $r^2=0.64$, $p<0.001$; PD $r^2=0.22$, $p=0.024$). Although error RTs were much faster for younger controls compared to older controls and patients, there were no differences between groups in the correction delay.

Figure S3

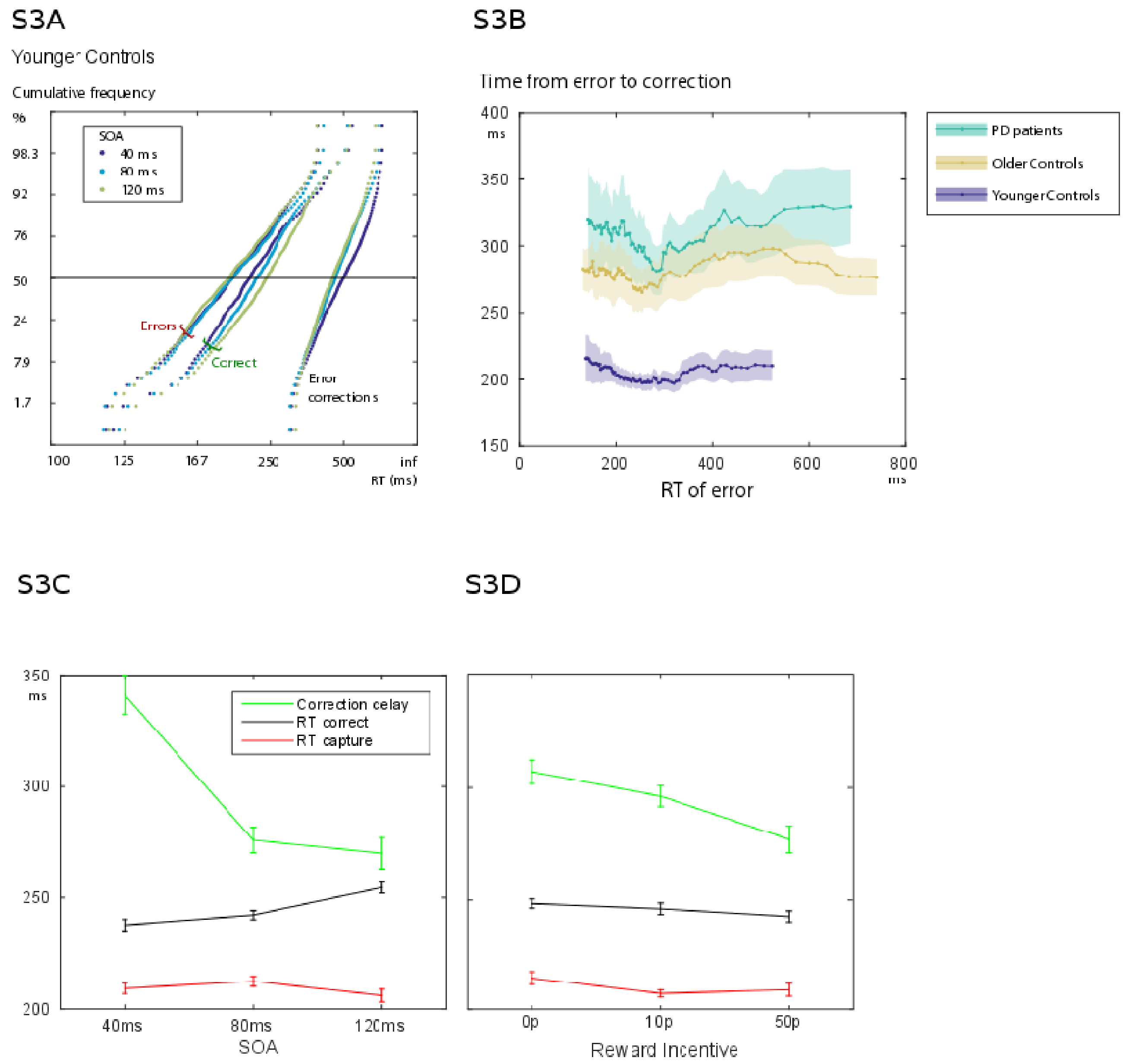

Figure S3: Analysis of time to correct an error (related to Fig.6C)

A) Cumulative histogram of reaction times for the younger 17 control participants. All times are relative to the onset of the distractor. For error trials, both the error RTs (far left) and times of the corrective saccade (far right) are plotted. For each participant, the RTs were quantiled, and proportion of saccades exceeding each percentile was plotted. The grand average across participants is plotted with one point per percentile bin; the axes are "reciprobit transformed" (Carpenter and Williams 1995) such that if 1/RT is normally distributed, the cumulative distribution appears as a straight line.

B) Relationship between time of error and time to correct the error. Previous studies have found that faster errors take longer to correct, and this has been taken to be evidence for a "race" between competing motor plans toward the distractor and target. To test this, for each subject, the joint distribution of error responses and corresponding error-corrections was examined using a sliding window. For errors that fell within each 20% quantile window, the mean of the corresponding error-correction delays were plotted. The error correction delay is the time interval from the initiation of the first saccade that landed on the distractor, to initiation of the first saccade that landed on the correct target. Within each group, there was no significant

effect of error RT on correction time. Error bars indicate standard error of the mean correction delay across individuals. N=25 for the younger controls, N=22 for older controls, and N=16 for PD patients

C) There was no effect of the distractor-target interval (SOA) on capture RTs (red). However, longer SOAs resulted in later correct responses (black), but faster error corrections (green). Error bars indicate standard error of the main effect of SOA.

D) Reward shortened RTs on correct trials, and also speeded up error correction RTs. Error bars indicate standard error of the main effect of reward.

Figure S4

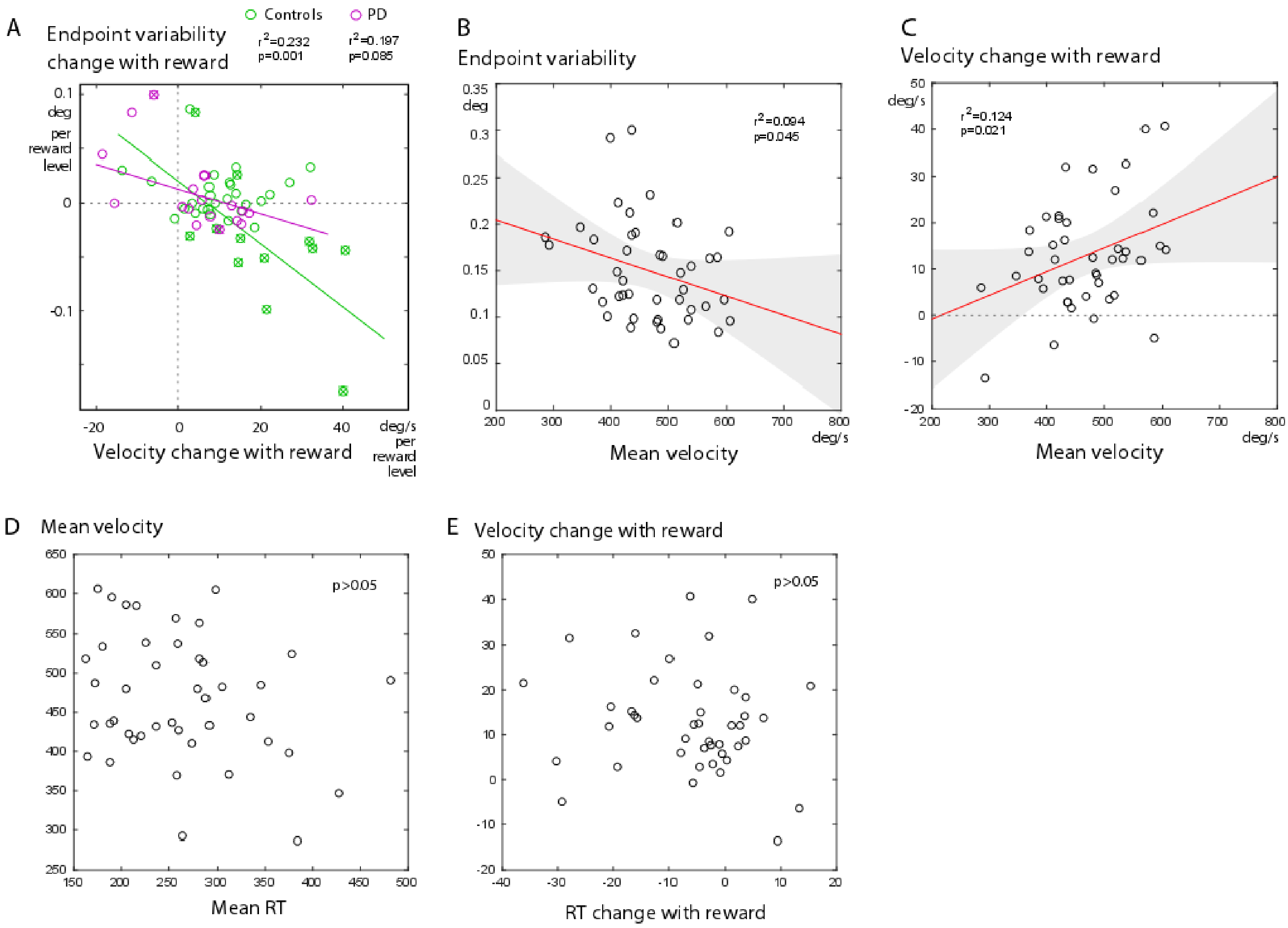

Figure S4: Correlation between reward sensitivity measures in healthy participants (related to Fig.6A)

In these plots, each subject is represented by a single point.

A) For each participant, sensitivity to reward was measured firstly for peak velocity, and secondly for amplitude variability, as a function of reward incentive. The values plotted on each axis correspond to the slope per unit reward, i.e. the degree of modulation by incentive. There was a significant negative correlation both in 39 healthy controls and in the PD patients, indicating that individuals who *increased their velocity* for high incentives also *decreased their endpoint variability*.

Our model predicts that endpoint variability may either increase or decrease with reward. Since there was no significant interaction between groups in the amount they modulated endpoint variability with varying reward, we asked whether individual participants had significant reward effects (as described in **Supplementary Methods**, section titled Statistics). Symbols filled with an 'x' indicate individuals who showed reward effects.

B) The overall endpoint variability for each individual was plotted against their overall mean velocity. There was a significant correlation indicating that participants who had faster saccade velocities also had more precise endpoint distributions. This effect is predicted from the model (Fig. **2C** left panels), where a participant with lower noise would be faster and less variable.

C) The size of the velocity change induced by reward was plotted against overall velocity for each participant. There was a significant positive correlation, indicating that faster individuals were able to increase their speed more. This is also predicted from the model (**Fig. 2C** lower panels), in which either a lower noise level or steeper temporal discounting would both lead to higher speeds but also stronger effects of reward on speed.

D) There was no correlation between a participant's mean RT and their mean peak saccade velocity ($r^2<0.1$, $p>0.05$).

E) There was also no correlation between the degree to which reward's influence on RT, and its influence on saccade velocity ($p>0.05$). Taken within the correlations between velocity and variability, and the correlation between reward sensitivity of velocity and variability (panels **A-C**), the lack of correlation between decision speed and movement speed suggests that decision and movement parameters may be optimised independently.

Figure S5

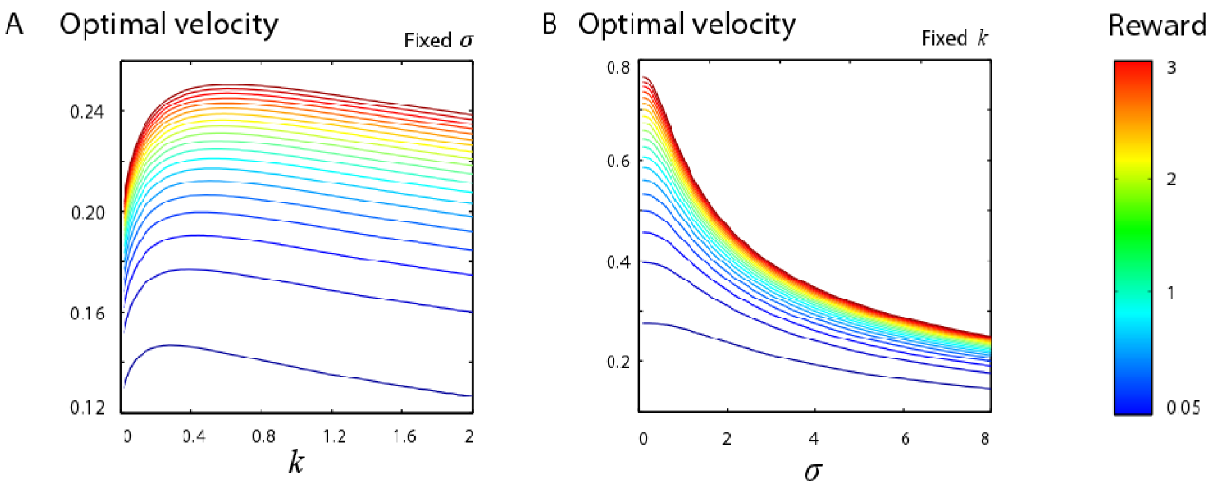

Figure S5: Effects of internal noise and temporal discount rate on velocity and motor precision (related to Fig.3C)

These figures show the same effects as in **Fig. 2C**, but with the model parameters on the x-axis. The values k and σ are fixed for an individual subject, but these plots allow us to examine how they influence behaviour. This provides a better visualisation of how temporal discount rate k and the difficulty of a task for an individual would alter their optimal movement velocity, and endpoint variability.

A) The optimal velocity of a movement initially increases with steeper temporal discounting. This is because if the value of a reward increases a lot with a small reduction in movement speed (i.e. very negative $\frac{d(EV)}{dT}$, for a given movement time T), then it pays to invest in speed. If the temporal discount rate becomes very high, then normal movement times fall in a flatter portion of the temporal discount curve. In other words, since reward has been discounted very heavily by time, a small change in speed will not affect the value much. Note that reward always increases the optimal velocity.

B) As the noise parameter σ increases, speeds are reduced. The level of noise may be due to individual differences, as well as task difficulty.

Figure S6

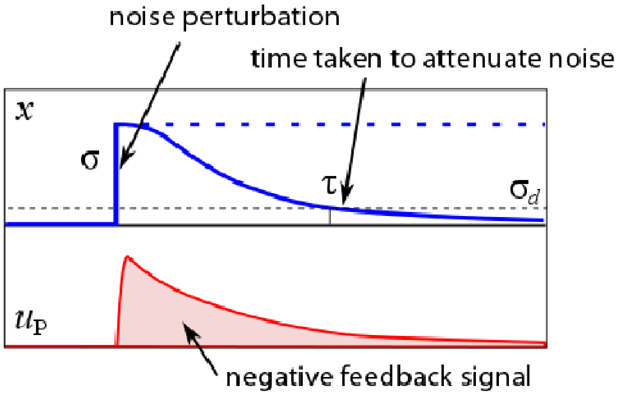

Input noise amplitude	σ
Time scale	τ
Desired noise amplitude	σ_d
Feedback required for critical damping	$u_P = 2\omega\dot{x} + \omega^2 x$
Required damping	$\omega \gtrsim \frac{1}{\tau}\log\left(\frac{\sigma}{\sigma_d}\right)$
Total cost of precision signal	$\int u_P^2 = \frac{1}{4}\sigma^2 \log\left(\frac{\sigma}{\sigma_d}\right)^3$
Observed noise depends on total precision signal	$\sigma_d = \sigma e^{-\frac{\lvert u_P \rvert \tau}{2\sigma}}$

Figure S6: How much negative feedback signal is required to attenuate noise? (related to Fig.1D)

Here we illustrate that it is possible to find a mathematical relationship between the size of feedback signals, and the reduction of noise.

We consider a second-order dynamic system in which control signals can be used to restore a system to its baseline state. The fastest way to accomplish this is to use critical damping. The "restoring force" depends on the size of the signal and its current rate of change, according to the equation $\ddot{x} + 2\omega\dot{x} + \omega^2 x = 0$. If the system deviates from its stable state, for example by an amount σ, then the restoring force will gradually return it to that state. After a time τ, the disturbance will have been attenuated to a lower amplitude. Let the desired level be σ_d after time τ. We calculate the total amount of negative feedback signal that will need to be expended, in such a system, to reduce the disturbance to a desired level. The final equation shows with a higher size of feedback signal u_P , we obtain a more quickly responsive system, and for a given time scale, the resultant noise σ_d is reduced. The equation also shows that if $\sigma \ll |u_P|\tau$ the observed noise is close to zero, but when $\sigma \gg |u_P|\tau$, there is a constant subtractive attenuation of observed noise, with $\sigma_d \approx \sigma - \frac{1}{1}|u_P|\,\tau$. This final result is reported in the **Supplementary experimental procedures** section, "The cost of noise reduction".

The purpose of this derivation is to demonstrate that if signals are costly, for example in terms of neuronal firing rates, then we can put a price on cancelling out noise.

Table S1

	**Temporal discount rate** ***k***	**Motor noise** **σ**	**Baseline variability** **σ_0**
**Healthy controls**	0.22 (0.03)	0.68 (0.08)	0.058 (0.028)
**Parkinson's disease**	0.17 (0.29)	0.94 (0.11) *	0.102 (0.032)

Table S1: Fitted model parameters for healthy participants and patients

Estimates for the three fitted parameters shown as medians across individuals (with standard error). PD patients had significantly higher motor noise σ compared to age-matched healthy control participants. Other parameters did not differ significantly. This is consistent, in our model, with an increased cost for controlling motor noise in PD.

Table S2

Healthy participants (n=39)		
	Mean	SD
Age	46.2	19.7
Sex	28 M	12 F
**PD Patients (n=19)**		
Age	65.3	9.0
UPDRS	23.1	10.1
Hoehn and Yahr Stage	1.8	0.86
Schwab and England daily living scale	86%	14%
Levodopa equivalent dose	507	240
HADS Depression score	3.6	3.0
Sex	9 M	10 F
**Matched Controls (subset of healthy participants, n=22)**		
Age	62.5	8.9
Sex	10 M	12 F

Table S2: Demographics of participants

Forty healthy volunteers were recruited from a public advert and UCL psychology subject pool. All participants had normal or corrected-to-normal vision. Data from one participant was corrupted on disk and was not analysable.

Nineteen patients with mild-to-moderate Parkinson's disease were recruited from the neurology clinic at the National Hospital for Neurology and Neurosurgery at Queen Square, UCL London. Ten patients were taking dopamine agonists (6 ropinirole, 3 pramipexole, 1 rotigotine), and 15 were taking levodopa. The levodopa equivalent dose was calculated from standard conversions [S1]. One patient was taking amitriptyline at night for sleep problems. There was no significant difference in age between patients and controls ($t(38)=1.01$, $p>0.05$).

Table S3

Model	1: Full model	2: Speed-accuracy trade-off only	3: Motivation only
**Parameters**	u_F and u_P vary with reward.<br>k, σ, σ_0 fixed for each subject	u_F varies with reward.<br>k, σ, σ_0 and u_P fixed for each subject	u_P varies with reward.<br>k, σ, σ_0 and u_F fixed for each subject
**AIC**	-206.6	-200.1	-154.6

Table S3: Model comparison for the drift-diffusion model fit

Three models were fitted to the data. The full model optimises both the force and precision for each reward level, to optimise expected value, predicting the velocity and endpoint variability. Model 2 keeps precision fixed and allows force to vary with reward, as in the standard speed-accuracy trade-off model—and thus predicts greater variability with reward. Model 3 keeps force fixed whereas precision can vary with reward. Model 1 fits significantly better than both of the simpler models.

Supplemental Experimental Procedures

Modelling the cost of control

1. Previous approaches predict invigoration by reward

Two previous approaches both predict that reward increases urgency. On one account, the organism expects an average ongoing reward rate. Movement duration and reaction time (RT) therefore count as a costly wasted time. If the ongoing expected reward rate is denoted by $\bar{R}^*$, then waiting a time T wastes reward $\bar{R}^* \cdot T$. Niv et al. (2005) showed that, if fast actions carry a cost proportional to $1/T$, then expected value (EV) of an action is given by

$$EV = \text{constant} - \bar{R}^* \cdot T - \frac{\epsilon}{T} \quad (3)$$

where ϵ is an effort cost. High rewards therefore lead to faster optimal movement times, given by

$$T^* \propto \frac{1}{\sqrt{\bar{R}^*}} \quad (4)$$

Optimal movement time = 1 / √(expected optimal ongoing reward rate)

An alternative formulation of time pressure is that delayed rewards are worth less. *Temporal discounting* may be modelled as an exponential or hyperbolic decay, such that the subjective value of a reward is higher if it is obtained earlier. A highly simplified form of the cost functions used by Rigoux and Guigon [S2] and Shadmehr et al. [S3] gives the value of an action as

$$EV = \frac{R}{1 + kT} - \frac{\epsilon}{T} \quad (5)$$

where k denotes temporal discount rate. This results in an optimal movement time T^* (**Fig.2A**):

$$T^* \propto \frac{\sqrt{R} + \sqrt{\epsilon k}}{R + \epsilon k} \quad (6)$$

These two formulations are in fact equivalent if $\epsilon k \ll R$, both predicting invigoration by incentives, with movement speed approximately proportional to $1/\sqrt{R}$.

On the other hand, there may be two reasons to favour *slower* movements. To see this, we denote the motor command by **u.** The vector **u** is a set of instructions varying over time, that represents, for example, the neural output to different muscles. Faster movements require larger motor commands **u**. First, speed carries an energetic cost, often taken to depend on the squared magnitude of **u**, that is, the sum of squares of all individual control commands. Second, motor commands are subject to signal-dependent noise, so faster movements suffer from greater variability. So to weigh up speed advantages with speed costs, we include the probability of landing on the target P_{win}, which falls with increasing motor noise. We obtain:

$$EV(\mathbf{u}) = R \times D(\mathbf{u}) \times P_{win}(\mathbf{u}) - |\mathbf{u}|^2 \quad (7)$$

Expected value of action = Reward × temporal discount × probability of reward given a motor command – (size of motor command)^2

where D is the temporal discount factor. There is an optimal value of **u** that balances the need to go

fast (maximise *D*) and accurately (maximise P) at low expense (minimise $|\mathbf{u}|^2$).

2. Feedback control signals could attenuate internal noise

Optimal motor control describes how control signals $\mathbf{u}(t)$ should be chosen in order to maximise a value function. The control signals alter the state of the body/world $\mathbf{x}$. However, the system's actual output is stochastic[S4], so $\mathbf{x}$ does not always track its desired trajectory[S5, 6]. Control signals can use sensory feedback to correct for perturbations in $\mathbf{x}$ due to noise. The optimal way of doing this is by computing an *internal estimate* of the world's state, $\hat{\mathbf{x}}$, and applying a Kalman gain to the difference between the observed and expected states of the system. [S7]. In effect, motor noise can be counteracted by feedback signals [S6].

Interestingly, this error-correcting feedback can be applied not only to motor noise, but also to *internal* noise [S8, 9]. The command $\mathbf{u}$ may include "internal" signals, which influence the internal state of the organism, rather than immediate motor output [S10, 11, 6, 12]. In this case $\hat{\mathbf{x}}$ contains not only representations of the world's state, but also internal state. The internal state estimates, corrupted by noise, can be steered back towards their desired states using the internal components of control signals. In general, the transformations at one hierarchical level of the motor system can generate error signals for a higher level [S13]. By including higher-level, non-motor commands or "predictions" in $\mathbf{u}$, in addition to muscle-level output commands[S14], internal noise-compensation could account for precision improvements in ballistic movements, when no external feedback is available.

To make this transparent, we allow the command to have two components—one for the standard motor command, $\mathbf{u}_F$, and another that represents the *precision* signals, i.e. the degree to which motor noise is attenuated, $\mathbf{u}_P$. The notion of a precision signal allows us to remain agnostic about the actual signals that attenuate noise, but asserts that they are costly. The larger the precision command $\mathbf{u}_P$, the lower the effective amount of noise in the force generated by $\mathbf{u}_F$, but the higher the cost. A particular choice of control command may therefore encapsulate not only the force of the movement, but also the precision.

3. Optimisation with a novel precision signal

We consider a simplified, single-command model where both force u_F and the novel precision control signal u_P, are just single scalar numbers. Optimal choice of action involves selecting a precision/force pair, $\mathbf{u}=[u_P, u_F]$, which minimises $|\mathbf{u}|^2$ and maximises expected reward value:

$$EV(u_P, u_F) = R \times D(u_F) \times P_{win}(u_F, u_P) - u_P^2 - u_F^2 \tag{8}$$

where the discount rate D is a decelerating positive monotonic increasing function of the force u_F, and the chance of success P_{win} decreases with force but increases with precision. The general optimal solution for $\mathbf{u}$ is given by a pair of differential equations:

$$u_F^* = \tfrac{1}{2} R \tfrac{\partial}{\partial u_F} (P_{win}(u_F, u_P) D(u_f)) \tag{9}$$

$$u_P^* = \tfrac{1}{2} R \cdot D(u_F) \tfrac{\partial P_{win}(u_F, u_P)}{\partial u_P}. \tag{10}$$

Given expressions for $P_{win}(u_F, u_P)$ and $D(u_F)$, equations (9) and (10) can be numerically solved for the optimal motor command.

4. How much does noise reduction cost?

It may be possible to relate the size of a precision command to reductions in noise amplitude. We

consider two possible mechanisms: firstly, a *negative feedback* signal to attenuate noise, and secondly, duplication of a signal over *many independent channels*, to average out the noise.

In the first case, consider a signal that is perturbed by noise (or deflected by an irrelevant input) of a given size. We can ask: how large a control signal is required to annul this perturbation? For a second-order control system with critical damping, we can calculate the total amount of control signal (the "restoring force") required to return the system to its desired state. After the perturbation, a control signal of amplitude u_P can lead to a disturbance σ being reduced to $\sigma e^{-\frac{u_P \tau}{2\sigma}} \approx \sigma - \frac{1}{2} u_P \tau$ over a given timescale τ (**Fig. S6**). If control signals u_P are costly in virtue of their amplitude, then we have a relationship between the size of the noise perturbation σ, and the cost for attenuating it within a given time.

In the second case, consider a signal that is duplicated in a number of channels, and that each channel is independently corrupted by noise. Averaging the noisy channels yields an estimate of the original signal. If u_P independent channels are used, noise is reduced by a factor of $\sqrt{u_P}$[S15]. There are two costs in duplicating the signal: first there is a higher total amount of signal, proportional to u_P (e.g. more neuronal action potentials are used), and second, there is an opportunity cost (e.g. from representing a signal more widely in the brain, at the expense of other signals)[S16].

For further calculations we use the second formulation. However both accounts provide an *estimate of the cost for reducing noise*, in terms of the energy expended in the neural precision signal. In principle, any relationship in which cost increases with increasing precision, could be incorporated into our framework.

5. Applying the cost of control to saccades

Here we derive more formally the equation in **Fig.1**. To estimate the EV for a saccadic task, we must find expressions for delay discounting *D*, and the chance of success P_{win}, in equation (8). We employ a hyperbolic temporal discount function of the action duration[S17], $1/(1+kT)$. For P_{win}, we must estimate the probability of obtaining the reward. We assume that movements end in a Gaussian distribution around an intended target, and are only rewarded if they end within a fixed radius of a target[S2]. For instance, the distribution of endpoints may have a width proportional to the movement force signal, but inversely proportional to the precision signal. The value function has two free parameters: temporal discount rate *k*, and intrinsic motor noise level σ, and is proportional to:

$$EV\left(\mathbf{u}_F, \mathbf{u}_P\right) \propto \frac{R}{1 + k \cdot T(\mathbf{u}_F)} 2\Phi\left(\frac{\text{precision}(\mathbf{u}_P)}{\sigma \cdot \text{force}(\mathbf{u}_F)}\right) - \left|\mathbf{u}_F\right|^2 - \left|\mathbf{u}_P\right|^2 \tag{11}$$

Expected value of action = Reward / (1+ discount rate × movement time) × probability of landing on target – cost of control signal

where Φ denotes the cumulative Gaussian error function.

To compute the time for a movement **u**, we make the simplifying assumption that **u**(*t*) respects a constant profile scaled over time and amplitude. For constant movement amplitude, the movement time *T* is inversely proportional to $\sqrt{u_F}$, since the distance travelled is proportional to $\iint_0^T \mathbf{u_F}\, dt\, dt$. We assume a specific movement amplitude is desired—consideration of variable amplitudes has been explored by other authors [S18–20]. Next, we take the noise to be signal-dependent, i.e. proportional to force u_F[S21], but reduced by our new precision signal by a factor $\sqrt{u_P}$ (see previous section). This gives an effective noise amplitude = $\sigma\, u_F / \sqrt{u_P}$. Substituting these into the EV equation gives:

$$EV(\mathbf{u}) \propto \frac{R}{1+k/\sqrt{u_F}} 2\Phi\left(\frac{\sqrt{u_P}}{\sigma u_F}\right) - u_F^2 - u_P^2 \tag{12}$$

where σ is a subject's baseline noise, and k their temporal discount rate. The cost of a command $|\mathbf{u}|^2 = u_F^2 + u_P^2$ represents the integral over the duration of the movement, and is scaled to match the reward units. This total $\int |\mathbf{u}|^2$ may represent the *overall effort* invested in a movement, including both physical energy, and the cost of precision control signals, i.e. the effort of overcoming noise. This quantifies the intuition that effort is not only physical, but also incorporates being precise in the face of noise.

Maximising EV predicts how the optimal force u_F and precision u_P should vary as a function of the available reward R. Numerical solutions for optimal u_F and u_P are portrayed in **Fig.3B**, as a function of reward and subject parameters k and σ. Velocity increases with reward, similar to previous formulations of vigour [S3, 22]. But critically, reward can *simultaneously* decrease movement variability. Under specific conditions, reward may also induce classical speed-accuracy trade-offs. In particular, when the signal-to-noise ratio is low, reward leads to slow and accurate choices (**Figure 4E**)[S23], and when the temporal discount rate is high, reward will lead to fast and erroneous movements (**Figure 3C**).

What does the noise parameter σ represent? Since endpoint variability is $\frac{\sigma u_F}{\sqrt{u_P}}$, σ is simply the endpoint variability of a fixed-speed movement, under fixed motivational conditions, relative to the target size—i.e. an individual's task-relevant motor noise. However, to account for the possibility that not all noise may be controllable by a system (e.g. noise in the effector itself), an additive baseline noise term σ_0 can be included. This latter term therefore represents error that would be present even in the highest motivational state. In this case, σ expresses the amount of investment required to reduce noise to a given level. It is thus the relative *cost of a precision signal*, compared to energetic (force) cost. The value 1/σ therefore signifies the signal-to-noise gain per unit of investment of reward.

An alternative way of parameterising equation (12) emphasises the cost of control. If control signals are independently scaled, with coefficients to determine their costs, equation (8) becomes:

$$EV(u_P, u_F) = R \times D(u_F) \times P_{win}(u_F, u_P) - w_F u_F^2 - w_P u_P^2 \tag{13}$$

where the ratio of the weights w_F and w_P determines the relative costs of precision and physical energy. These re-arrangement shows explicitly that σ (in Eqn.12) indicates the cost of precision. It also includes an additional degree of freedom, in which the scaling of reward is also free to vary.

6. Drift diffusion simulation

The drift diffusion model allows us to predict the reaction time distribution and error rate of a two-alternative choice decision[S24, 25]. The outcome of a decision depends primarily upon three parameters, the average rate of accumulation of information μ, the threshold θ at which enough information is available to make a decision, and σ, the amount of noise in the accumulator (**Fig. 4A**).

Improvements in decision-making are often considered to be "top-down" or "attentional" effects. We suggest that in a hierarchical control system, the inputs from a higher level can be construed as reducing noise or unwanted perturbations in the lower level (see "The cost of noise reduction", above). To implement this, we take the *signal-to-noise ratio* of an accumulator to be increased by a single scalar precision signal, which carries a cost. An organism might therefore select not only the threshold, but also the noise in the decision, in order to optimise EV. For example a precision u_P might result in an effective noise level $\sigma/\sqrt{u_P}$, at a cost $|\mathbf{u}|^2 = u_P^2$. The time taken is now the RT,

and P_{win} is the error rate, both calculated by simulating the diffusion process. These values are substituted into equation (7), and the optimum threshold and precision can then be found, which in turn determine speed and accuracy (**Fig. 4B-E**). Performance depends on the reward on offer, the individual's baseline signal-to-noise ratio μ/σ, and their temporal discount rate. High reward adds time pressure but also encourages *investment in precision*—enabling the classical speed-accuracy trade-off to be broken by motivation.

Simulations of decisions were run, finding the optimal θ and u_P to maximise EV. Each run simulated 2000 trials, and results were averaged over 10 runs. Parameters that were varied included 12 levels of reward ranging (0.25–3), 8 levels of the signal μ (1–8), and 3 levels of accumulator noise σ = 1, 2 and 3. Diffusion proceeded according to

$$\delta A = \left(\mu + \frac{\sigma}{\sqrt{u_P}}\mathcal{N}\right)\delta t; A(0) = 0 \tag{14}$$

such that the accumulator A increases or decreases with mean rate μ, perturbed by a Gaussian random variable N which is scaled by the internal noise σ, divided by the precision signal u_P. A time step of 1 ms was used, with noise amplitude σ/10 per timestep, and temporal discount rate of 0.1 s^{-1}.The RT for each trial was the first time step at which either A > θ or A < $-\theta$. For each condition, the EV was calculated (using Eqn. 7), assuming that reward that falls off over time as e^{-T^2} (similar results were obtained with e^{-kT} and $1/(1+kT)$). The precision u_P and threshold θ maximising EV was found for each condition (combinations of R and μ/σ) using a pattern search with 10 random starting points for each condition. This method takes into account the shape of the RT distribution. The cost of one unit of precision was scaled to 70 reward units, to produce baseline RTs of the order 200 ms—times which are typical for the saccadic system. Trials on which the RT was greater than 1 second were considered as errors (i.e. zero reward). Finally, the mean optimum RT and accuracy was calculated for each condition. Results are shown in **Fig. 4.**

These simulations showed that reward increased accuracy (**Fig. 4B** and **4D**). When signal-to-noise μ/σ was *high*, reward also shortened the optimal RT. In contrast, with very low signal-to-noise ratios (**Fig. 4E,** blue lines), increasing reward actually prolonged RT, leading to a classical speed accuracy trade-off. This reflected a greater investment in precision with reward, despite a lower threshold, in situations where a task was difficult. This task-dependence might explain why reward's effects on speed and accuracy vary from study to study [S26, 27].

Some drift diffusion models have also included stochastic starting points of the accumulator, such that $A(0)$ is chosen from a uniform distribution in a range ±α [S28–30]. This allows higher error rates for early responses, as seen in our data. Qualitatively similar results were obtained using a starting-point variability of α=0.1 and 0.6, rather than zero.

We examined the predicted conditional accuracy function and delta plot (effect of reward on shortening RT as a function of RT bin). For this distributional analysis, starting point variability was fixed at ±0.3, and temporal discount rate was 5 s^{-1}, to generate fast errors. Predictions were generated for three levels of reward, and 500 responses were simulated 20 times. The effect of reward was plotted using the same techniques as for the experimental data, using a moving window over RT quantiles to calculate reward effect on accuracy and RT (**Fig. S1B** and **D**). These plots illustrate the qualitative match between the empirical effects of reward, and those predicted by the model.

The methods above allow motivational effects to be predicted. Such effects have previously been summarised in terms of attention, alertness or arousal, but have not been quantified before in terms

of cost-benefit analysis. In its basic form, the ability to attenuate noise has the effect of increasing the signal-to-noise ratio when weighing up uncertain evidence. The cost could equally be applied to race models of decisions[S31], for example by amplifying the difference in build-up rate of competing processes.

Empirical quantification of reward's effect on speed and accuracy

1. Task Instructions

Forty healthy volunteers participated, mean age 46 (Supplemental Data **Table S2**). Participants were informed that they had to keep their eyes on the illuminated disc, while they listened to how much money was available on the upcoming trial. When the display changed, the remaining two discs would light up, one slightly later than the other. The aim was to look towards the disc that illuminated second. They were told that they had to move as quickly as possible, and that the time they took to reach the target would be used to calculate the *proportion of the stake* they actually won. The total winnings would be added up and paid in cash after the experiment.

2. Task

The task is a variant of the double step paradigm [S32, 33] and aimed to maximise oculomotor capture by the salient distractor [S34]. Three screen locations were indicated by dim grey discs, each 4° diameter, arranged in an equilateral triangle 11.4° apart (**Fig. 5A**). One disc was illuminated in yellow colour at the start of the trial, and participants were required to fixate this for 500 ms to start the trial. Participants heard a recording of a voice speaking "0p maximum", "10p maximum" or "50p maximum" lasting 1200 ms, followed by a variable foreperiod of 400-600 ms. Then the target was dimmed and simultaneously one of the two remaining discs brightened (the distractor). After a delay, the third disc brightened also (the target). The delay was one of 40 ms, 80 ms or 120 ms for the younger participants (n=18), and was fixed at 80 ms for the older control participants (n=22) and PD patients. The target display remained visible until a saccade terminated at the target disc.

The time taken to reach the target (from distractor onset until gaze arrived at the target) was used to calculate reward (**Fig. 5B**) as follows:

$$R(t) = R_{\max} \cdot \min\left(1 - e^{\frac{t-\tau_2}{\tau_1}}\right) \quad (15)$$

where R is reward for the current trial, t is the time taken to reach the target, $R_{\max}$ is the maximum reward that could be won on a given trial, and τ_1 and τ_2 are adaptive reward criteria (see below).

Reward was displayed as a red integer in the target disc as soon as the target was reached, for 800 ms. This was accompanied by a bell sound when the reward was 10p or greater, or a 'cash register' sound when 30p or greater was won. The target location was then used as the starting point for the next trial.

Unknown to participants, the RT criteria τ_1 and τ_2 were adaptively adjusted using the last 20 trials. The criteria tracked quantiles of the RT distribution, keeping 10% of trials faster than τ_1 and 30% of trials slower than τ_2. This ensured that participants experienced the full range of outcomes irrespective of their baseline reaction speed.

Participants performed 4 blocks of 54 trials each, with a 2 minute break between blocks, with drift-correction before each block. There were three reward cues of 0p, 10p, 50p, three possible starting

locations, and two possible target locations relative to this starting location. Trials were intermixed and balanced between blocks.

3. Materials

Stimuli were presented on a CRT resolution 1280 x 1024 pixels at 100 Hz, at a distance of 60 cm from the eye, controlled by MATLAB and PsychToolbox. Eye movements and pupil size were recorded by a desktop-mounted Eyelink 1000 Hz infra-red eye tracker with head rest, with online parsing of saccade endpoints for reward determination. Velocity and endpoints were calculated offline (described below). Auditory reward cues were presented through a loudspeaker beneath the desk.

4. Saccade analysis

Saccades were parsed using criteria on velocity of $30°s^{-1}$, acceleration > $8000°s^{-2}$ and amplitude > 1°. Saccadic RTs were calculated as the time from distractor onset until this threshold was exceeded. Responses were classified according to the endpoint of the first saccade (**Fig. 5C**).

The trial was classified according to the first saccade made after the onset of the distractor. Correct trials were those in which the saccade's amplitude was greater than 5°, and its endpoint was closer to the target than the distractor. The trial was classed as an oculomotor capture error if the first saccade's amplitude was greater than 5°, and its endpoint was closer to the distractor than to the target. Other trials (average 4.8%) were discarded. The lenient accuracy criteria, and large diameter of target discs, aimed to minimise the need for endpoint precision in this task.

For correct trials, the peak velocity of the first saccade was calculated using 4 ms windows from saccade onset to termination, discarding any speeds greater than $3000°s^{-1}$, and any saccades during which tracking was lost. Saccade amplitude was defined as the distance from fixation to the first saccade's endpoint. Amplitude variability was calculated as the standard deviation of saccade amplitudes for each reward condition for each subject.

5. PD Patients

Nineteen patients with mild or moderate PD who fulfilled the criteria for the Queen Square Brain Bank for PD (Gibb and Lees 1988) were recruited from the neurology clinic at the National Hospital for Neurology and Neurosurgery. The mean UPDRS was 23.1 (s.d. 10.1). All patients were on medication; 15 were taking levodopa, and 10 were taking a dopamine agonist. The mean levodopa equivalent dose was 507 mg (**Table S2**).

The mean age of the patients was 65.3 yrs (s.d. 9.0) compared to 62.5 (s.d. 8.9) for the older control participants. Cognitive impairment was screened for using either Montreal cognitive assessment (MoCA) ≥ 26 or mini-mental state examination (MMSE) score ≥ 26; two patients had mild cognitive impairment with MoCA of 25. Two patients did not have cognitive tests but were still in full-time work. Depression was excluded using the Hospital Anxiety and Depression scale [S35]. All patients had normal or corrected-to-normal colour vision. Symptomatically, 2 patients had significant functional impairment as determined by Schwab and England ADL score (one patient 50%, one patient 60%, all other patients 80% or above).

Sixteen patients were tested at 9 am, and three at 2 pm. Eight patients had previously performed the task 2 weeks earlier. Before undertaking the rewarded oculomotor capture task, patients also completed 96 trials of a simple prosaccade task and 96 trials of a simple antisaccade task, as a baseline measure (not reported here). Then the oculomotor task was performed as described above. One patient completed only 192 trials out of 216, and the analysis was conducted on the reduced

number of trials.

6. Statistics

The effect of reward in healthy participants was assessed by calculating the mean RT, mean peak saccade velocity, proportion of oculomotor capture (errors), and variability in saccade amplitude for each condition (**Fig. 6**). The proportion of capture was arcsine transformed. A repeated measures general linear model yielded the effect of reward on each of the four measures. To compare patients with PD with controls (**Fig.7**), a mixed-effects model was used with Group as a between-subjects factor. This yielded main effects of reward and group, plus an interaction term for group x reward.

The cost-of-control model predicted that with certain combinations of noise and temporal discount (low σ and high k), motor variability might *increase* with reward rather than decrease (**Fig. 3C**, upper graphs). Since the group-level effects of reward and PD on variability were not significant (trend to interaction p=0.077), we examined whether individuals had effects of reward in either direction. A significant effect of reward (for one participant) was defined as the variability difference between the lowest reward and the highest reward level lying outside the confidence intervals for this difference when the trials were permuted over the reward levels (5000 permutations, threshold p=0.05). Of the 39 control participants, 12 had significant effects of reward: 10 negative (reduction of variability with reward) and 2 positive in direction, after correction for 39 multiple comparisons. Of the 19 PD patients, 2 had significant effects, one positive and one negative. A scatter plot of the effects (**Fig. S4A**) shows the significant individuals with crosses. The reward effects on variability were found to correlate strongly with effects on velocity in the control group (r^2=0.232, p<0.001), with a similar trend in the PD group (r^2=0.20, p=0.085).

7. Velocity effects were not attributable to amplitude or curvature

Saccade velocity is known to be relatively rigidly determined by saccade amplitude [S36, 37], according to a law known as the "main sequence"[S38]. Can the invigorating effect of reward be attributed simply to increased saccade amplitude when higher incentives were available? Saccade amplitudes did increase with larger rewards, although the absolute effect was small: mean hypometria of 0.04° with high reward compared to 0.26° for low reward (F(2,76)=18.4, p<0.001). Using stepwise regression to subtract out the effect of amplitude, velocity remained significantly increased by incentives over and above what was predictable by amplitude changes (F(2,76)=9.38, p=0.002). This indicates that rewards invigorate movement speed *independently* of movement size, transgressing the "main sequence" relationship between velocity and amplitude[S38].

Saccades were sometimes curved, either towards or away from the distractor (**Fig. 5C**). Could the slower velocities observed with low incentives simply be due to increased trajectory curvature? To factor out curvature, the maximal deviation of the saccade trajectory away from a straight line was used as a covariate in stepwise regression against saccade velocity, to obtain residuals that did not depend on curvature. There was no effect of curvature on velocity (t=0.45; p>0.05), and reward significantly increased velocity even when curvature was regressed out (t(38)=6.64, p<0.001).

8. Conditional accuracy functions and Delta plots

Reward may have differential effects on slower and faster responses. The distribution of RTs was analysed by novel sliding-window analyses (**Figs. 6F** and **S1**), which improve upon previous binned (Vincentised) methods [S39, 40]. Although quintile binning improves significantly on a simple mean or median [S41], firstly it assumes that 5 bins is the appropriate number, and secondly, the bin edges are chosen are essentially arbitrary [S42]. Furthermore, parametric statistical approaches rely heavily on selecting appropriate hypotheses about the different RT bins. To overcome these limitations, we devised continuous sliding window versions of the conditional accuracy function,

examining accuracy as a function of RT, and a continuous "delta plot", which examines the effect of reward on RT as a function of RT.

Firstly, we asked how reward influenced *accuracy* at different reaction times. To do this, we constructed a conditional accuracy function [S43] (**Figs. S1A** and **S1B**), in which a sliding bin of width 20% quantile was moved smoothly, in one-percentile steps, along the RT distribution. The proportion of correct saccades was calculated in each window. To test for significant effects of reward, correcting for multiple comparisons over multiple windows, a permutation test was performed by randomly re-ordering the reward conditions within each subject's data, and computing across the whole time series the maximum value of the t-statistic [S44]. The resulting null distribution of maximum *t* over all the permutations can be thresholded at a given alpha-level to control the family-wise error rate (the probability that at any of the many t-tests across the timepoints will be significant, over all permutations). Comparing the t-statistic of the actual data to the null distribution yielded a corrected p-value for time window.
Early responses were more likely to be oculomotor capture errors, as shown in the conditional accuracy plot (i.e. accuracy in each RT time bin, **Fig. 6F**). For a given reward level, a speed-accuracy trade-off holds. When reward is increased, the conditional accuracy function undergoes a parallel shift, contravening this trade-off. Healthy controls showed clear effects of reward ($p<0.05$ for RT bins < 284 ms), but crucially, the reward effect was absent in PD patients ($p>0.05$, **Fig. S1A**). Individuals with PD were generally slower than controls, and more accurate at later time-bins, yet *less* accurate at early time bins.

Secondly, we examined the effect of reward on RT at different time-points during the RT distribution (**Fig. S1C**). A similar analysis was conducted to obtain "delta plots" which compared the RT distributions of different reward levels[S45]. A 20% quantile bin was moved smoothly, in one-percentile steps, over the two RT distributions, and the means are subtracted, to give a point-wise estimate of the effect of reward. The analysis was performed for both 10p–0p and 50p–10p, and the average of these two was plotted. Significance of reward effects was calculated using a permutation test. The effect of reward on accuracy at a given bin was compared to the null distribution (i.e. reward levels permuted) of the maximum *t* statistic over all bins.

Reward significantly shortened RT in controls (blue) through most of the RT distribution ($p<0.05$), unlike in PD. Later responses showed the greatest speeding by reward. This effect of RT was also predicted by the simulation (**Fig. S1D**). Thus the moment-to-moment effects of incentive are consistent with the control cost model.

9. Fatigue could not explain reduced reward sensitivity in PD

Fatigue is known to be more prevalent in PD, and might impact reward sensitivity. To exclude the possibility that motivational effects were related to fatigue, trends over time-on-task for velocity, RT, variability and accuracy were measured. There were no significant effects on velocity, endpoint variability, or oculomotor capture. RT did decrease with time on task ($F(1,37)=10.4$, $p=0.003$), so we compared the effects of reward on early (first half) and late (second half) trials, and found no interaction with reward, and no interaction with group ($F(1,120)=2.3$, $p=0.13$). Thus none of the reported effects were attributable to time-on-task.

10. Analysis of distractor-target delay and error trials

In order to look for evidence that race processes generated the observed behaviour, the probability of oculomotor capture errors was broken down by the distractor-target delay in the young healthy control group (n=17). Longer delays led to more errors (**Fig. S2A**, repeated-measures 1-way ANOVA $F(2,32)=3.77$; $p=0.034$).

The reaction time on error trials was measured as the time from the distractor onset until the first saccade greater than 5 degrees that ended closer to the distractor than the target. The error RTs were consistently shorter than correct RTs (**Figure S2B**), consistent with a race model interpretation, or a drift-diffusion model with variable initial state. The time to correct each error was determined as the time from the error RT until the eyes landed on the correct target. Across subjects, these times correlated strongly with the error RT (**Figure S2C**, r^2=0.52, p<0.001). These times were significantly longer than both correct and error RTs (mean 305 ms, s.e.m. 18). The mean cumulative distributions of error RT, correct RT and correction RT were plotted on reciprocal-x probit-y axes, on which a normally distributed rate (1/RT) would appear as a straight line [S31]. Quantiles of the cumulative RT distribution were averaged across participants (**Fig. S3A**), showing that correct responses were later than errors, with a narrower variability, and stronger effects of delay.

To examine whether, on a trial-by-trial basis, the time of an error determined the time of its corresponding correction, the mean time to correct an error was plotted as a function of the error RT bin (**Fig. S3B**). A sliding window of width 20 percentiles was moved along the error RTs in 1 percentile intervals. Fast errors took relatively longer to correct than later errors, consistent with previous reports[S32, 46]. However for the slowest error responses the reverse effect was seen. We suggest that those trials may represent "lapses", on which both the error and the correction were slow. The correction time was much shorter when the distractor-target delay was longer ($F(2,32)=20$, p<0.001), suggesting parallel preparation of movements to the distractor and target (**Fig. S3C**). Reward markedly reduced the RT of error-corrections (**Fig. S3D**).

Model fit of reward's effects on motor control

We fitted the cost of control model to velocity and endpoint error data from each participant. First, the optimal command $[u_F, u_P]$ was found (**Fig. 3B, C**) for each point in a 50 x 50 x 50 volume (logarithmic ranges, reward = 0.1 to 10, σ = 0.1 to 100 and k = 0.01 to 10). This predicts, for each combination of subject-level parameters σ, k, and σ_0, how a subject's variability and velocity would vary with reward. Each participant's actual mean peak velocity and saccade amplitude variability were measured at each reward level. These data were then fitted to the parameters k, σ and σ_0, by minimising the squared error of the model predictions to the data (across velocity and variability for each of the 3 reward levels). Thus 3 parameters were obtained for each participant. Since the units in the model were arbitrary, four more constants were fitted *across all* participants. These were: the unit of variability= 0.61°, unit of velocity =103°/s, reward unit = 7 pence, minimum reward level = 19% of maximum reward.

1 .Model comparison

We compared our precision-and-force model ("Model 1") with two simpler models. Model 2 allowed each participant to 'choose' only the force u_F for each reward level, with the precision u_P fixed across reward levels. This model therefore only allows for a standard speed-accuracy trade-off, and is equivalent to the "orthodox" model. Model 3 held the force constant, but allowed individuals to 'choose' the precision for each reward level. This model thus includes *only* effects that would be traditionally regarded as "attentional" or motivational. All models had three parameters that characterised each subject: k, σ and σ_0.

To determine if both of these dimensions of variability are required to explain the data, the Akaike information (AIC) was calculated for each of the 3 models, across all participants, using least squares log likelihood[S47]. AIC measures a model's fit, accounting for its complexity. The model with the lowest AIC is strongly supported if the AIC difference is larger than 4 (corresponding to a probability ratio of 0.02). According to AIC, our new force-and-precision model fits the data best (**Table S3**).

2. Patient vs control comparison

Which model parameter best accounted for the difference between patients with PD and controls? To answer this, the best fitting parameters (σ, σ_0, k) for each participant were found, using the best model, i.e. the model that allowed both u_F and u_P to vary. Across participants, both σ and σ_0 followed a Gaussian distribution (according to the Kolmogorov-Smirnoff test), but k was skewed, and was therefore log-transformed, restoring normality, before comparison. PD patients were compared with an age-matched subset of healthy controls, n=22. Patients had significantly increased noise control costs σ (two-tailed unpaired t-test, $t(36)=2.21$, $p=0.034$, **Table S1**). Neither their temporal discount rate nor their baseline variability were significantly different from healthy people ($p>0.05$). One interpretation of these data is that PD patients go slower in order to reduce their motor variability in the face of an increased cost for controlling internal noise.

Our results are suggestive, but not conclusive, that dopamine depletion may lead to a higher cost of control. Although our patients had mild-to-moderate PD without dementia, we cannot rule out pathology in non-dopaminergic systems, e.g. cholinergic and serotonergic dysfunction[S48]. On physiological grounds, however, it has been suggested that dopamine itself may have direct effects of increasing synaptic gain[S49], or suppressing membrane noise in sensory or motor neurones to improve signal-to-noise ratios[S50]. Thus it could be suggested that dopamine, by enhancing signal-to-noise, may reduce the cost of control. Future work could unite the role of dopamine in reward and effort by considering how it generates performance improvements at the neural level.

Supplementary References

S1. Tomlinson, C. L., Stowe, R., Patel, S., Rick, C., Gray, R., and Clarke, C. E. (2010). Systematic review of levodopa dose equivalency reporting in Parkinson's disease. Mov. Disord. *25*, 2649–2653.
S2. Rigoux, L., and Guigon, E. (2012). A Model of Reward- and Effort-Based Optimal Decision Making and Motor Control. PLoS Comput Biol *8*, e1002716.
S3. Shadmehr, R., Xivry, J. J. O. de, Xu-Wilson, M., and Shih, T.-Y. (2010). Temporal Discounting of Reward and the Cost of Time in Motor Control. J. Neurosci. *30*, 10507–10516.
S4. Shadmehr, R., and Krakauer, J. W. (2008). A computational neuroanatomy for motor control. Exp. Brain Res. *185*, 359–381.
S5. Davis, M. H. A., and Vinter, R. B. (1985). Stochastic Modelling and Control (Dordrecht: Springer Netherlands) Available at: http://cds.cern.ch/record/1619764 [Accessed August 11, 2014].
S6. Todorov, E. (2005). Stochastic Optimal Control and Estimation Methods Adapted to the Noise Characteristics of the Sensorimotor System. Neural Comput. *17*, 1084–1108.
S7. Qian, N., Jiang, Y., Jiang, Z.-P., and Mazzoni, P. (2012). Movement Duration, Fitts's Law, and an Infinite-Horizon Optimal Feedback Control Model for Biological Motor Systems. Neural Comput. *25*, 697–724.
S8. Chen-Harris, H., Joiner, W. M., Ethier, V., Zee, D. S., and Shadmehr, R. (2008). Adaptive Control of Saccades via Internal Feedback. J. Neurosci. *28*, 2804–2813.
S9. Joiner, W. M., FitzGibbon, E. J., and Wurtz, R. H. (2010). Amplitudes and directions of individual saccades can be adjusted by corollary discharge. J. Vis. *10*, 22.
S10. Haruno, M., Wolpert, D., and Kawato, M. (2001). MOSAIC Model for Sensorimotor Learning and Control. Neural Comput. *13*, 2201–2220.
S11. Todorov, E. (2004). Optimality principles in sensorimotor control. Nat. Neurosci. *7*, 907–915.
S12. Adams, R. A., Shipp, S., and Friston, K. J. (2013). Predictions not commands: active inference in the motor system. Brain Struct. Funct. *218*, 611–643.
S13. Krakauer, J. W., and Mazzoni, P. (2011). Human sensorimotor learning: adaptation, skill, and beyond. Curr. Opin. Neurobiol. *21*, 636–644.
S14. Jiang, J., Heller, K., and Egner, T. (2014). Bayesian modeling of flexible cognitive control. Neurosci. Biobehav. Rev. Available at: http://www.sciencedirect.com/science/article/pii/S0149763414001390.
S15. Seung, H. S., and Sompolinsky, H. (1993). Simple models for reading neuronal population codes. Proc. Natl. Acad. Sci. *90*, 10749–10753.
S16. Johnston, W. A., and Strayer, D. L. (2001). 15 A dynamic, evolutionary perspective on attention capture. In Advances in Psychology, Charles L. Folk and Bradley S. Gibson, ed. (North-Holland), pp. 375–397. Available at: http://www.sciencedirect.com/science/article/pii/S0166411501800170.
S17. Haith, A. M., Reppert, T. R., and Shadmehr, R. (2012). Evidence for Hyperbolic Temporal Discounting of Reward in Control of Movements. J. Neurosci. *32*, 11727–11736.
S18. Trommershäuser, J., Maloney, L. T., and Landy, M. S. (2003). Statistical decision theory and trade-offs in the control of motor response. Spat. Vis. *16*, 255–275.
S19. Trommershäuser, J., Gepshtein, S., Maloney, L. T., Landy, M. S., and Banks, M. S. (2005). Optimal Compensation for Changes in Task-Relevant Movement Variability. J. Neurosci. *25*, 7169–7178.
S20. Schütz, A. C., Trommershäuser, J., and Gegenfurtner, K. R. (2012). Dynamic integration of information about salience and value for saccadic eye movements. Proc. Natl. Acad. Sci. *109*, 7547–7552.
S21. Harris, C. M., and Wolpert, D. M. (1998). Signal-dependent noise determines motor planning.

Nature *394*, 780–784.
S22. Niv, Y., Daw, N. D., Joel, D., and Dayan, P. (2007). Tonic dopamine: opportunity costs and the control of response vigor. Psychopharmacology (Berl.) *191*, 507–520.
S23. Bijleveld, E., Custers, R., and Aarts, H. (2009). The Unconscious Eye Opener Pupil Dilation Reveals Strategic Recruitment of Resources Upon Presentation of Subliminal Reward Cues. Psychol. Sci. *20*, 1313–1315.
S24. Ratcliff, R. (1979). Group reaction time distributions and an analysis of distribution statistics. Psychol. Bull. *86*, 446–461.
S25. Ratcliff, R., and Frank, M. J. (2012). Reinforcement-Based Decision Making in Corticostriatal Circuits: Mutual Constraints by Neurocomputational and Diffusion Models. Neural Comput. *24*, 1186–1229.
S26. Rorie, A. E., Gao, J., McClelland, J. L., and Newsome, W. T. (2010). Integration of Sensory and Reward Information during Perceptual Decision-Making in Lateral Intraparietal Cortex (LIP) of the Macaque Monkey. PLoS ONE *5*, e9308.
S27. Wang, L., Yu, H., and Zhou, X. (2013). Interaction between value and perceptual salience in value-driven attentional capture. J. Vis. *13*. Available at: http://www.journalofvision.org/content/13/3/5.
S28. Ratcliff, R. (1981). A theory of order relations in perceptual matching. Psychol. Rev. *88*, 552–572.
S29. Ratcliff, R., and Rouder, J. N. (1998). Modeling Response Times for Two-Choice Decisions. Psychol. Sci. *9*, 347–356.
S30. Ratcliff, R., and McKoon, G. (2007). The Diffusion Decision Model: Theory and Data for Two-Choice Decision Tasks. Neural Comput. *20*, 873–922.
S31. Carpenter, R. H. S., and Williams, M. L. L. (1995). Neural computation of log likelihood in control of saccadic eye movements. Nature *377*, 59–62.
S32. Camalier, C. R., Gotler, A., Murthy, A., Thompson, K. G., Logan, G. D., Palmeri, T. J., and Schall, J. D. (2007). Dynamics of saccade target selection: Race model analysis of double step and search step saccade production in human and macaque. Vision Res. *47*, 2187–2211.
S33. Ramakrishnan, A., Sureshbabu, R., and Murthy, A. (2012). Understanding How the Brain Changes Its Mind: Microstimulation in the Macaque Frontal Eye Field Reveals How Saccade Plans Are Changed. J. Neurosci. *32*, 4457–4472.
S34. Theeuwes, J., Kramer, A. F., Hahn, S., and Irwin, D. E. (1998). Our Eyes do Not Always Go Where we Want Them to Go: Capture of the Eyes by New Objects. Psychol. Sci. *9*, 379–385.
S35. Zigmond, A. S., and Snaith, R. P. (1983). The Hospital Anxiety and Depression Scale. Acta Psychiatr. Scand. *67*, 361–370.
S36. Bahill, A. T., Clark, M. R., and Stark, L. (1975). The main sequence, a tool for studying human eye movements. Math. Biosci. *24*, 191–204.
S37. Harris, C. M., and Wolpert, D. M. (2006). The Main Sequence of Saccades Optimizes Speed-accuracy Trade-off. Biol. Cybern. *95*, 21–29.
S38. Chen, L. L., Hung, L. Y., Quinet, J., and Kosek, K. (2013). Cognitive regulation of saccadic velocity by reward prospect. Eur. J. Neurosci. *38*, 2434–2444.
S39. Vincent, S. B. (1912). The functions of the vibrissae in the behavior of the white rat ... (University of Chicago).
S40. Ratcliff, R. (1979). Group reaction time distributions and an analysis of distribution statistics. Psychol. Bull. *86*, 446–461.
S41. Dawson, M. R. W. (1988). Fitting the ex-Gaussian equation to reaction time distributions. Behav. Res. Methods Instrum. Comput. *20*, 54–57.
S42. Rouder, J. N., and Speckman, P. L. (2004). An evaluation of the Vincentizing method of forming group-level response time distributions. Psychon. Bull. Rev. *11*, 419–427.
S43. Wood, C. C., and Jennings, J. R. (1976). Speed-accuracy tradeoff functions in choice reaction time: Experimental designs and computational procedures. Percept. Psychophys. *19*, 92–102.

S44. Nichols, T. E., and Holmes, A. P. (2002). Nonparametric permutation tests for functional neuroimaging: A primer with examples. Hum. Brain Mapp. *15*, 1–25.
S45. Ridderinkhof, K. R., W.P.M. van den Wildenberg, J. Wijnen, and B. Burle (2004). Response Inhibition in Conflict Tasks Is Revealed in Delta Plots. In Cognitive neuroscience of attention (Guilford Press), pp. 369–377. Available at: http://dare.uva.nl/record/158289 [Accessed April 2, 2013].
S46. Becker, W., and Jürgens, R. (1979). An analysis of the saccadic system by means of double step stimuli. Vision Res. *19*, 967–983.
S47. Akaike, H. (1980). Likelihood and the Bayes procedure. Trab. Estad. Investig. Oper. *31*, 143–166.
S48. Francis, P. T., and Perry, E. K. (2007). Cholinergic and other neurotransmitter mechanisms in Parkinson's disease, Parkinson's disease dementia, and dementia with Lewy bodies. Mov. Disord. *22*, S351–S357.
S49. Ashby, F. G., and Casale, M. B. (2003). A model of dopamine modulated cortical activation. Neural Netw. *16*, 973–984.
S50. Kroener, S., Chandler, L. J., Phillips, P. E. M., and Seamans, J. K. (2009). Dopamine Modulates Persistent Synaptic Activity and Enhances the Signal-to-Noise Ratio in the Prefrontal Cortex. PLoS ONE *4*, e6507.
